# A Systematic Review and Meta-Analysis of Physical Activity Interventions in Colorectal Cancer Survivors: An Evidence Evaluation Attempt Across Racial/Ethnic Groups

**DOI:** 10.3390/healthcare13243198

**Published:** 2025-12-05

**Authors:** Yves Paul Vincent Mbous, Rowida Mohamed, George A. Kelley, Kimberly Michelle Kelly

**Affiliations:** 1 School of Pharmacy, Department of Pharmaceutical Systems and Policy, West Virginia University, Morgantown, WV 26506, USA; 2 Pritzker School of Medicine, the University of Chicago, Chicago, IL 60637, USA; 3 School of Public and Population Health and School of Kinesiology, Boise State University, Boise, ID 83725, USA; georgekelley@boisestate.edu; 4 Preventive Medicine, the University of Tennessee Health Science Center, Memphis, TN 38163, USA; kkelly44@uthsc.edu

**Keywords:** colorectal cancer, minority, meta-analysis, physical activity, race, survivor

## Abstract

**Aims:** Recommendations for cancer survivors concur regarding physical activity (PA), and elucidating factors governing PA uptake among colorectal cancer (CRC) survivors is needed. This study examined the impact of PA interventions and investigated the variation in PA across several characteristics, including race/ethnicity. **Design:** We performed a systematic review and aggregate data meta-analysis of randomized controlled trials (RCTs) of PA interventions. Data Sources: We used studies from CENTRAL, PubMed (NCBI), PsycINFO (EBSCOhost), CINAHL (EBSCOhost) with full text, Scopus (ELSEVIER), and the Web of Science (CLARIVATE) (1 May 1993–1 September 2023). **Methods:** For the meta-analysis, the inverse variance heterogeneity (IVhet) model was used to pool standardized mean difference effect sizes (Hedge’s g) for our primary outcome, changes in PA. **Results:** Sixteen studies representing 1668 participants were included in the meta-analysis. A moderate, statistically significant increase in PA was observed (g = 0.44, 95% CI 0.12–0.76; *p* = 0.01). However, a large amount of inconsistency was observed (*I*^2^ = 80.8%, 95% CI, 36.1% to 90.9%), as well as major asymmetry suggestive of small-study effects (publication bias, LFK = 3.04). Only 28% of trials reported race/ethnicity, limiting equity analyses. Subgroups comparing atheoretical vs. theory-based interventions did not differ statistically. Meta-regression results suggested associations with specific behavior change theories and delivery features. Based on the Grading of Recommendations Assessment, Development and Evaluation (GRADE) assessment, the overall certainty of evidence was considered low. **Conclusions:** There is low-certainty evidence that PA interventions may improve PA among CRC survivors. Future trials should (i) recruit and report diverse samples in a clear and transparent manner, (ii) explicitly map theory constructs to techniques and test mechanisms, and (iii) report fidelity and clinically meaningful thresholds alongside behavioral outcomes.

## 1. Background

Globally, colorectal cancer (CRC) remains one of the most common and lethal malignancies, ranking third in incidence and second in mortality [1]. According to the most recent Global Cancer Statistics (GLOBOCAN 2024) update, there were approximately 1.93 million new CRC cases and 930,000 deaths worldwide, representing a modest increase since 2020 [1]. The global distribution of CRC reflects marked regional disparities—age-standardized incidence rates are highest in Europe, North America, and Oceania, where screening and early detection are widespread, while the mortality-to-incidence ratio (MIR) remains elevated in low- and middle-Human Development Index (HDI) countries, indicating limited access to timely diagnosis and treatment. CRC is the third leading cause of cancer-related deaths among men and women in the United States [2]. Moreover, CRC is higher among minorities, especially Blacks and American Indians, compared to other major racial and ethnic groups in the U.S [3]. Among Black men and American Indian women, CRC incidence is highest, whereas mortality rates are highest among Black men and Black women [3].

Research has demonstrated that physical activity (PA) reduces CRC incidence, mortality, and recurrence [4,5,6,7] and improves health-related quality of life in cancer survivors [8,9]. The American Cancer Society (ACS) and National Comprehensive Cancer Network (NCCN) recommend ≥150 min per week of moderate-intensity aerobic PA, ≥75 min per week of vigorous-intensity aerobic PA, or an equivalent combination of both [10,11,12,13]. However, adherence to PA is poor among cancer survivors, with approximately 44% not meeting current recommendations [14]. Notably, CRC survivors have some of the lowest PA adherence rates (~35%) compared to other cancer survivors [15]. Additionally, racial and ethnic disparities in PA adherence exist among CRC survivors, with Blacks being less physically active than Whites [16].

Several systematic reviews have examined the effectiveness of interventions in increasing PA among cancer survivors [17,18,19,20]. However, these reviews typically conflate various types of cancer survivors. To the best of our knowledge, only two reviews have summarized the evidence on the impact of interventions for improving PA uptake among CRC survivors [21]. However, these studies were: (1) restricted to works that used theory [21]; (2) only included CRC survivors who had completed primary treatment prior to recruitment and simultaneously assessed multiple outcomes [22]; (3) did not attempt to synthesize results according to race/ethnicity; (4) did not report 95% prediction interval, a valuable statistical measure that accounts for the heterogeneity and uncertainty inherent in combining data from multiple studies as well as providing a better estimate than a 95% confidence interval with respect to what result one might expect if they conducted their own randomized controlled trial (RCT) [23].

There is conflicting evidence about the influence of health behavior theory on interventions. Some affirm its effectiveness [24], whereas others find no significant differences between theoretical and atheoretical works [25]. Thus, the association between theory and intervention effectiveness remains poorly understood [26]. Further, to date, the effect of PA among racial/ethnic minority CRC survivors remains unexplored. Given racial and ethnic disparities in CRC incidence and PA uptake among CRC survivors, it is crucial to identify intervention features that work best among these groups. Additionally, to our knowledge, no study has evaluated the methodologies of these interventions to determine factors associated with increased PA among CRC survivors. Thus, the primary objective of this study was to conduct a systematic review and meta-analysis to determine the overall effects of PA interventions on PA changes among adults with CRC. Secondary aims were to analyze the relationship between changes in PA and reported study characteristics, especially theory use, as well as race/ethnicity.

## 2. Methods

This study was conducted based on the general guidelines of the Cochrane Collaboration [27], the Preferred Reporting Items for Systematic Reviews and Meta-Analyses literature search extension statement (PRISMA-S), and the Preferred Reporting Items for Systematic Reviews and Meta-Analyses (PRISMA) guidelines [28,29]. The protocol for this study was prospectively registered in PROSPERO (#CRD233335) and published in a peer-reviewed journal [30]. Deviations from our protocol, i.e., post hoc changes, occurred after data collection due to limited reporting and are described throughout the methods. The PRISMA checklist is available in Table S1.

### 2.1. Study Eligibility Criteria

Intervention studies with the following characteristics were included: (1) CRC survivors at all stages (≥18 years of age) identified as either inactive or not meeting the ACS/NCCN PA requirements at baseline [10,11], (2) RCT design (parallel-group or cluster, explanatory or pragmatic), (3) control group (passive, usual care, wait-list control, no treatment or attention-control), (4) four-week minimum intervention, (5) studies published in English language, (6) changes in PA and/or PA behavior uptake assessed. Measurement of PA could include objective measures (minutes/week, hours/week, MET-hours/week, or MET-minutes/week) or subjective measures (questionnaire scores). Exclusion criteria included studies with participants already meeting the PA requirements at baseline, unpublished work, studies published in non-English journals, and studies in which participants were physically incapacitated or dealing with illness or injury.

### 2.2. Study Search and Selection

The years for which the search was conducted were amended from our protocol to include evidence from 1 May 1993, up to 1 September 2023, collected using (1) Cochrane Central Register of Controlled Trials (CENTRAL), (2) PubMed (NCBI), (3) PsycINFO (EBSCOhost), (4) CINAHL with full text (EBSCOhost), (5) Scopus (ELSEVIER) and (6) Web of Science (CLARIVATE). Cross-referencing of the bibliographies from retrieved studies was also conducted. Individual database search strategies are shown in Table S2. Each search was downloaded as a separate file to Mendeley reference management software (version 1.19.4, Elsevier, London, UK). Duplicates were removed both electronically and manually. The precision of the searches was calculated as the number of included studies divided by the total number of studies screened [31]. The number needed to read (NNR) was then calculated as the inverse of the precision [31]. All database searches were conducted by the first and second authors. To minimize selection bias, two researchers (the first two authors) independently reviewed studies for eligibility by screening titles and abstracts against the eligibility criteria. If the inclusion/exclusion decision could not be made based on the title and abstract, the full texts were retrieved for further examination. The reasons for exclusion were recorded and categorized according to the PICOS framework. Disagreements were resolved by the senior authors. Using Gwet’s AC1 statistic [32], the overall agreement rate prior to correcting discrepancies was 0.95.

### 2.3. Data Abstraction

The first author developed an electronic codebook using an Excel spreadsheet (version 16.37) based on elements derived from previous research [33,34]. The developed codebook was pilot-tested and reviewed by the research team. Detailed information regarding the elements extracted is described in our protocol [30]. Post hoc, rurality was added as a covariate.

The inclusion criteria were not restricted by country, and each country may use its own methods to collect data on race and ethnicity. Due to the anticipated preponderance of US-based studies (and consistent with our PA standards), the U.S. Office of Management and Budget (OMB), the unit of the U.S. government responsible for the decennial national Census, guided our collection of racial and ethnic information [35], which has recently been updated [36]. Groups included White, Native Hawaiian or Other Pacific Islander, African American or Black, Asian, American Indian or Alaska Native, Hispanic or Latino, Middle Eastern or North African, and Some Other Race. Individuals were also allowed to indicate more than one race [37]. Other information about race/ethnicity was recorded, if available.

The primary outcome was the change in PA between the exercise and control groups. Secondary outcomes included changes in physical function, body mass index (BMI; kg·m^−2^), aerobic fitness, and upper- and lower-body strength. The primary outcome could be measured objectively (e.g., accelerometers and pedometers) or subjectively (e.g., questionnaires). If both measures were provided in a study, objective measures were prioritized; most studies used accelerometer-based assessments. For physical function, precedence was given to tests of walking performance, given their widespread use (treadmill test, 6-min walk test, etc.) [38], followed by reach testing (sit-and-reach tests) [39], and finally, by questionnaires or scales assessing physical/functional well-being. Aerobic fitness measures included commonplace assessments such as the maximum VO_2_ uptake and peak tests [40]. Upper body strength tests included standard validated tests in older populations, such as grip strength assessments [41], whereas lower body strength tests included assessments such as the 30-s chair stand [42] and knee extension tests [43].

Outcome data abstracted included baseline, intervention completion, and post-intervention values. The first two authors independently extracted data from each selected article. Discrepancies in coding and extraction were settled by consensus. Using Gwen’s AC1 statistic, the inter-rater agreement was 0.95. The first author requested missing data, and 43% of authors responded.

### 2.4. Risk of Bias Assessment, Strength of Evidence, Extent of Theory Use, and BCTs

The risk of bias was assessed independently by the first two authors using the revised Cochrane risk-of-bias tool for randomized trials (RoB 2) [44]. The RoB 2 tool evaluates potential bias across five domains: the randomization process, deviations from intended interventions, missing outcome data, outcome measurements, and selection of reported results. Each domain was rated as having a low risk, some concerns, or a high risk of bias. The rating of some concerns, which replaced the rating of unclear in the earlier version of the RoB instrument, implies a potential risk of bias that is not high enough to be rated as high risk, but still a cause for concern. More specifically, it suggests either a plausible risk of bias, for which the currently available evidence is insufficient for a definitive judgment, or a moderate potential for bias. Thus, a rating of some concerns suggests that trial results are unlikely to be reliable without additional study, but not necessarily completely invalid.

The overall strength of evidence was evaluated using the Grading of Recommendations Assessment, Development, and Evaluation (GRADE) instrument [45]. The 12-item Template for Intervention Description and Replication (TIDieR) checklist was used to rate the overall completeness of intervention reporting [46]. The first two authors performed these assessments independently. Any disagreements were discussed and resolved until a 100% agreement was reached. Using Gwet’s AC1 statistic, the overall agreement rate prior to correcting differences was 1.0. The Theory Coding Scheme (TCS) was used to assess the extent of theory use [47], while the Behavior Change Technique (BCT) taxonomy instrument (version 1) was used to identify the behavior techniques employed across each study [48]. Further details on the tools and assessment procedures are available in the published protocol. The first two authors performed these appraisals independently. Any disagreements were discussed until a 100% agreement was reached. Using Gwet’s AC1 statistic, the overall agreement rate prior to correcting discrepancies was 0.98.

### 2.5. Statistical Analysis

#### 2.5.1. Effect Size Calculation and Pooling

As per our protocol, pooled quantitative analysis was reserved for outcomes found in at least six studies. This criterion disqualified three of our secondary outcomes (aerobic fitness and upper- and lower-body strength), leaving changes in our primary outcome (PA) and two secondary outcomes (physical function and BMI) for use in the meta-analysis. The standardized mean difference (SMD) effect size (Hedge’s g), adjusted for small-sample bias, was computed for each study [49]. The SMD was calculated as the difference in change outcomes between the exercise and control groups, divided by the pooled standard deviation of the two groups. For studies in which change outcome values and/or standard deviations were not available, g was calculated from pre- and post-means and standard deviations in the exercise and control groups [50]. The IVhet model was used to pool effect sizes for all outcomes, and 95% confidence intervals (CIs) were generated. Non-overlapping 95% CIs were considered statistically significant. Forest plots were used to display individual and pooled point estimates. We chose the IVhet model over others because it has been shown through simulation studies to provide better error estimation than the commonly used Dersimonian and Laird (DL) random-effects model [51] and other random-effects models [52]. The DL random effects model is based on the exchangeable parameter assumption and was not used because its asymptotic behavior with very large trial numbers failed to show bias approaching zero for any distribution, which questions its validity [53]. Furthermore, the expected heterogeneity across studies (samples) is no longer an appropriate basis for model selection, as many models aim to incorporate it in error estimation (including both the IVhet and the DL random effects models). The first step in model selection was the assumption we were comfortable with, and then we selected a model under that assumption. Finally, a recent article recommended the IVhet model over the many other models currently available [54].

#### 2.5.2. Stability and Validity of Outcomes

Heterogeneity was examined using Q statistics, with *p*  ≤  0.10 considered to be statistically significant [55]. Inconsistency was examined using I-squared (*I*^2^). While somewhat arbitrary, values < 25%, 25–50%, and >50% corresponded to small, medium, and large levels of between-study inconsistency, respectively [56]. Small-study effects (publication bias, etc.) were assessed qualitatively using the Doi plot and quantitatively using the Luis Furuya-Kanamori (LFK) index [51,57,58]. Sensitivity analyses were performed to examine (1) the influence of each study on the overall results, (2) studies with different units of measurement, and (3) the use of different definitions of survivorship. According to the National Cancer Institute, cancer survivorship is defined as having a CRC diagnosis from the time of initial diagnosis and throughout one’s lifetime [59]. Other applicable definitions of survivorship that refer only to those with a prior history of the disease who have completed treatment were also considered acceptable but were not used across any of the selected works [60]. Cumulative meta-analysis, ranked by year, was performed to examine the accumulation of results over time [61]. Outlier analysis was also conducted by excluding results for effect sizes whose 95% confidence intervals (CIs) fell entirely outside the pooled 95% CI. We conducted subgroup analyses for outcomes with at least four effect sizes per group, as recommended by the Cochrane Handbook [62]. These included changes in PA and physical function. We also conducted a subgroup analysis of theoretical and non-theoretical interventions. However, due to limited data and inconsistent reporting, no meta-analysis by race and/or ethnicity was conducted. Rather, results were described qualitatively. Post hoc, 95% prediction intervals (PIs) and z-tests were also calculated. Two-tailed alpha values < 0.05 were used to determine between-group statistical significance. No adjustments were made for multiple testing because we sought to avoid missing potentially important findings that could be tested in original, randomized controlled trials [63].

#### 2.5.3. Meta-Regression

Meta-regression analyses based on the IVhet model were used to examine the relationship between changes in PA and selected covariates. Analyses were limited to variables with at least ten effect sizes for a continuous covariate and at least four effect sizes per group for a categorical covariate [64]. Two-tailed z-values ≤ 0.05 and non-overlapping 95% confidence intervals were considered to represent a statistically significant association. Potential predictor variables, established a priori, are listed in our protocol [30]. The following software was used for analysis: Stata (version 16), including the user-written metan and LFK routines within Stata, Mendeley (version 1.19.4), JabRef (version 5.1), Microsoft Excel (version 16.37), an add-in for Excel, MetaXL (version 5.3), and the Comprehensive Meta-Analysis Prediction Intervals worksheet.

#### 2.5.4. Deviation from the Protocol

Although assessment of racial and ethnic disparities was an a priori aim, the feasibility of this analysis was constrained by the limited and inconsistent reporting of these variables across included studies. Race and ethnicity were often inconsistently reported or omitted entirely. As a result, only a descriptive summary of the available race/ethnicity data could be provided.

## 3. Results

### 3.1. Study Characteristics

**Figure 1:**
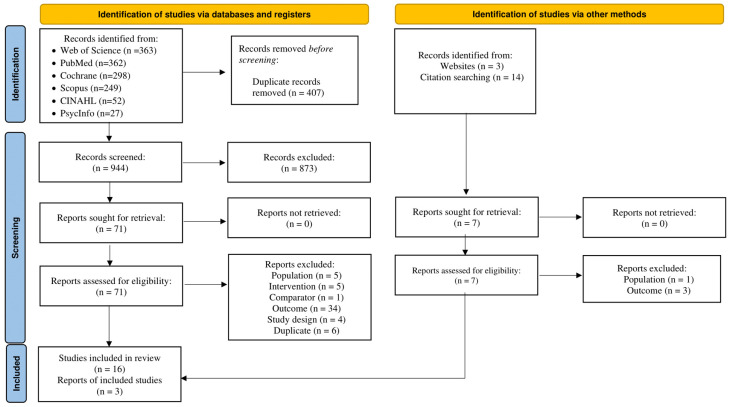
Flowchart of screening and selection process leading to the final study pool.

A flow diagram of the screening process results is shown in Figure 1. List of excluded studies with reasons for exclusion are presented in Table S3. Of the 944 citations reviewed, 19 were selected for systematic review [65,66,67,68,69,70,71,72,73,74,75,76,77,78,79,80,81,82], and 16 for meta-analysis [65,66,67,68,69,71,72,73,74,75,76,77,78,79,82,83]. Reasons for exclusion at the full-text screening stage included: mixed cancer survivor population (no CRC specific PA outcome) [84,85,86,87,88,89,90], no baseline PA data [91], baseline PA ≥ 150 min/week [92,93,94,95], wrong study design [96,97,98], no comparator group [99], no primary PA outcome [81,100,101,102,103,104,105,106,107,108,109,110,111,112,113], different interventions [114,115,116,117,118], and duplicates of previously selected or excluded studies [119,120,121,122,123,124,125]. Reasons for exclusion during the abstract/title screening are listed in Table S3. Selected studies were conducted in the U.S (n = 5) [65,69,76,77,78], Canada (n = 3) [73,75,79,83], Republic of Korea (n = 3) [67,68,72], United Kingdom (n = 2) [74,82], Australia (n = 2) [66,75], the Netherlands (n = 3) [70,71,73], Denmark (n = 2) [73,81], Italy (n = 1) [73], Spain (n = 1) [73], France (n = 1) [73], and Sweden (n = 1) [80]. All studies employed a parallel-group RCT design. Control participants were assigned to usual care (n = 11), wait-list control (n = 3), or educational control (n = 5). The NNR was 52 articles per included article.

### 3.2. Participant Characteristics

Table 1 provides a description of participant characteristics. The number of participants per study ranged from 8 to 410 [66,80], while the mean age ranged from 54 to 79 years [73,79,80]. Across 13 studies, stages III and IV were, respectively, the most common CRC stages, with ranges between 26.8% to 87.7% and 2.0% to 8.7%, respectively [66,67,68,69,71,72,75,76,78,79,81,82,83]. Time since CRC diagnosis ranged from 6.2 to 104 months (n = 5) [65,66,69,75,77], and time since treatment completion ranged from 2.4 to 16.6 months (n = 8) [67,68,72,74,78,81,83,95]. Race/ethnicity data were provided for five of the 19 (27.8%) studies [65,69,76,77,78], all of which were conducted in the U.S. Three of the studies that provided race/ethnicity data asked a single question on race, as evidenced by their provision of only racial information, whereas the remaining trials provided information that combined race and ethnicity [65,77], with unclear methods for ascertainment. Across studies that reported racial demographics, one reported four categories [69], one reported three categories (White, Black, and Other) [78], and three reported two categories (White vs. Other [65,76] and White vs. Black [77]). The most represented race was White, accounting for 73.0% to 97.8% of the samples. The percentage of Black participants was small, ranging from 2.0% to 15% of the samples. Only one study included American Indians/Alaskan Natives and Native Hawaiians or Pacific Islanders [69]. Asians, as categorized by the OMB, were included in the same study, comprising 12% of the sample. Four of the five studies saw improvement in PA in the intervention arm, but perhaps because sample sizes were small (n = 101 minority participants across five studies), individual studies did not include subgroup analyses for PA. Along with studies from the US, three studies from South Korea that presumably included only Asians reported increased PA in the intervention arm. Given the former, the lack of appropriate demographic data limited the assessment of racial and ethnic disparities. Rurality, a post hoc variable not initially included in our protocol [30], was not assessed in any of the studies.

### 3.3. Intervention Characteristics

Intervention characteristics, such as delivery settings, methods, and PA types are provided in Table 2. Most interventions lasted 12–24 weeks, were delivered twice weekly, and focused on moderate-intensity aerobic exercise. Intervention length ranged from 4 to 57 weeks [75,79], with a median of 14 weeks. Two sessions per week was the most common frequency (n = 5) [66,70,73,74,75,76]. Intervention intensity (n = 7) ranged from 55% to 85% of the age-predicted maximum heart rate [70,71,73,74,77,78,83]. The duration of interventions ranged from 10 to 90 min per session (n = 13) [65,67,68,69,70,72,73,74,77,79,80,82,83]. PA was measured using objective measures (n = 6) [69,73,78,80,81,82], subjective measures (n = 13) [65,66,67,68,70,71,72,73,74,75,76,79,83], or both objective and subjective methods (n = 1) [77]. Participants who adhered (range, 38–94%) and completed the study (range, 74–97.5%) were reported in nine [66,70,74,75,77,78,79,80,82] and two cases [80,82], respectively. Training and knowledge of the intervention personnel were provided in 13 studies [66,67,68,70,71,72,73,74,75,76,78,79,82]. Intervention personnel included exercise physiologists (n = 2) [74,78], certified exercise specialists (n = 7) [67,68,72,73,75,76,82], trained health coaches (n = 1) [66], physiotherapists (n = 1) [70], trained nurses (n = 1) [71], and trained kinesiologists (n = 1) [79].

The Theory of Planned Behavior (n = 3) [69,75,82], Social Cognitive Theory (n = 2) [70,77], and Transtheoretical Model (n = 2) [71,77] were commonly applied. The extent of theory use is provided in Table S4. Information on relevant theoretical construct targets, recipient selection based on theory, the link between techniques and theory, measurement of theoretical constructs, mediation effects, and theory refinement was either poorly defined or absent. As elements of the TCS, predictors of targeted behavior (constructs) were rarely mentioned (n = 2) [69,82], and evidence of the use of theory in selecting recipients more likely to benefit from intervention or in tailoring the intervention to the needs of a particular individual was entirely missing. Thus, the extent to which the intervention targeted a particular construct was seldom clarified (n = 1) [82]. No attempts were made to measure constructs or predictors of behavior or to test and/or refine the theory ultimately.

As shown in Table S5, the commonly applied BCTs included goal-setting behavior (n = 18) [65,66,67,68,69,70,71,72,74,75,76,77,78,79,80,82,83], goal-setting outcomes (n = 15), action planning (n = 14), instruction on how to perform a behavior (n = 13), self-monitoring of behavior (n = 12), information about health consequences (n = 10), persuasive argument (n = 8), and problem-solving (n = 8).

Dropout rates ranged from 5.6% to 23.0% of the original sample size [66,67,68,69,70,71,72,74,75,76,77,78,79,80,81,82,83]. The length of follow-up ranged from 4 weeks [79] to 2 years [75]. All RCTs reported using intention-to-treat analysis. Major reasons for dropout included: (1) treatment-related side effects; (2) unrelated medical problems; (3) unable to contact; (4) deceased; (5) refusal and consent withdrawal.

### 3.4. Risk of Bias, GRADE, and TIDiER Assessment

Across all studies, the risk of bias assessment (Figure S1) indicated “some concerns,” mainly related to the measurement of physical activity outcomes, missing outcome data, deviations from intended interventions, and selection of the reported results. These issues were consistent with the GRADE findings shown in Table S6. Overall, a high risk of bias was observed in the measurement of outcomes, in unreported deviations from intended interventions, and in small-study effects. The TIDiER checklist results (Table S7) showed that informational material (n = 7), post hoc modifications, and adherence and fidelity assessment were not well described. Accompanying discrete item evaluation for risk of bias is shown in Figure S2.

### 3.5. Outcome Assessment

#### 3.5.1. Changes in PA (Primary Outcome)

**Figure 2:**
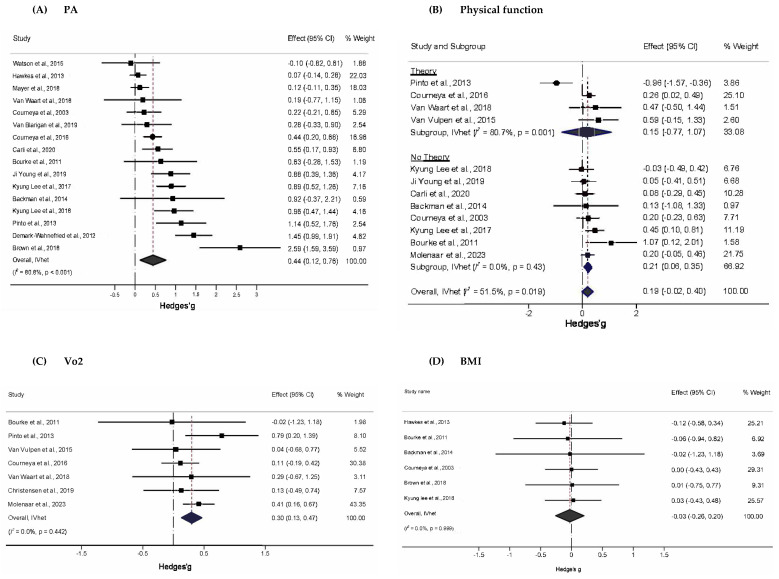
Forest plot for standardized effect size changes in (**A**) PA, (**B**) Physical function, (**C**) VO_2_, and (**D**) BMI (Hedge’s g). The black-filled squares, sized according to the weight contributing to the overall effect, represent changes in PA from each study, while the left and right extremes of the squares represent the lower and upper 95% confidence intervals for changes in PA from each study. The black diamond represents the pooled effect size change in PA, while the left and right extremes of the diamonds represent the pooled lower and upper 95% confidence intervals for changes in PA. The red dashed vertical line through the middle of the diamond represents the pooled mean effect, while the black dashed vertical line represents the zero (0) point. % Weight: Percentage weight of a particular study [65,66,67,68,69,70,71,72,73,74,75,76,77,78,79,80,81,82,83].

Only ten studies led to PA adoption per the ACS and NCCN recommendations [65,66,67,68,72,75,77,78,81,83]; uptake among the remainder, albeit significant, did not meet the recommended level. Figure 2A depicts the forest plot for the standardized mean difference changes in PA. There was a statistically significant, moderate improvement in PA (g = 0.44, 95% CI, 0.12 to 0.76, *p* = 0.01), statistically significant heterogeneity (Q = 78.2, *p* < 0.001), and a large amount of inconsistency (*I*^2^ = 80.8%, 95% CI, 36.1% to 90.9%). This corresponded to a 5.5% greater between-group improvement in physical activity in the exercise group compared to the control group. The absolute between-study variance of the true effect size, tau-squared ($\tau^{2}$), which was 0.18, while the 95% PI for what might be expected if a new trial were conducted in similar populations included zero (−0.54 to 1.42). Major asymmetry suggestive of small-study effects (e.g., publication bias, etc.) was observed (LFK = 3.04, Figure S3A). Two outliers (atheoretical interventions) were detected, and their deletion from the model did not have a major effect on the overall findings (g = 0.37, 95% CI, 0.13 to 0.61, Q = 40.1, *p* < 0.001, *I*^2^ = 67.6%, 95% CI, 43.4% to 81.5%) [65,78]. Influence analysis, with each study deleted from the model once, showed that results remained statistically significant across all deletions (Figure S4A). A cumulative meta-analysis revealed that results have been statistically significant since 2017 (Figure S5A).

#### 3.5.2. Physical Function (Secondary Outcome)

Figure 2B shows a small, statistically non-significant improvement in physical function (g = 0.19, 95% CI: −0.02 to 0.40). Statistically significant heterogeneity (Q = 22.7, *p* = 0.02) and a moderate amount of inconsistency (*I*^2^ = 52%, 95% CI, 6.4% to 74.9%) were found, while $\tau^{2}$ was 0.05 and the 95% PI included zero (−0.36 to 0.74). No small-study effects were observed (LFK = 0.27, Figure S3B). Outlier and influence analysis revealed a single outlier whose removal resulted in statistically significant improvements in physical function (g = 0.23, 95% CI, 0.11 to 0.36, Q = 8.3, *p* = 0.6, *I*^2^ = 0%, 95% CI, 0% to 51.9%, Figure S4B.) [77]. With two exceptions, a cumulative meta-analysis showed that studies prior to 2014 yielded non-significant results, whereas those from 2015 onwards yielded significant results (Figure S5B).

#### 3.5.3. VO_2_ (Secondary Outcome)

As shown in Figure 2C, PA interventions produced a statistically non-significant increase in peak exercise VO2 (g = 0.30, 95% CI, 0.13 to 0.47). Overall inconsistency was null (*I*^2^ = 0%, 95% CI, 0% to 57.3%), and heterogeneity was non-significant (Q = 5.83, *p* = 0.44). $\tau^{2}$ was 0, and there were two outliers [73,76]. The exclusion of the outliers produced a non-significant decrease in the overall findings (g = 0.11, 95% CI, −0.13 to 0.36, Q = 0.221, *p* = 0.99, *I*^2^ = 0%, 95% CI, 0% to 0%). Minor asymmetry suggestive of small-study effects was observed (LFK = −1.50, Figure S3C). Influence analysis showed no statistically significant results upon each deletion (Figure S4C). Cumulative analysis showed that results were consistently significant, with but one exception in 2011 (Figure S4C).

#### 3.5.4. BMI (Secondary Outcome)

As shown in Figure 2D, PA interventions produced a statistically non-significant overall change in BMI (g = –0.03, 95% CI –0.26 to 0.20), with a general tendency toward slight reductions in most interventions. However, the 95% CI was wide, reflecting uncertainty in the estimate. No statistically significant heterogeneity (Q = 0.24, *p* = 1.00) was observed, and overall inconsistency was null (*I*^2^ = 0%, 95% CI, 0% to 0%). Absolute between-study heterogeneity ($\tau^{2}$) was 0, and no outliers were found. Minor asymmetry suggestive of small-study effects was observed (LFK = −1.26, Figure S3D). Influence analysis showed no statistically significant results upon each deletion (Figure S4D). Cumulative analysis showed that results were consistently non-significant (Figure S5D).

#### 3.5.5. Subgroup Analyses

**Figure 3:**
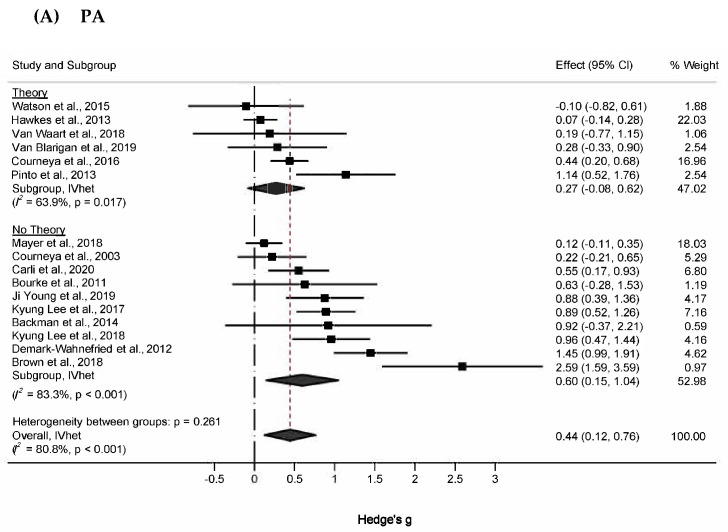

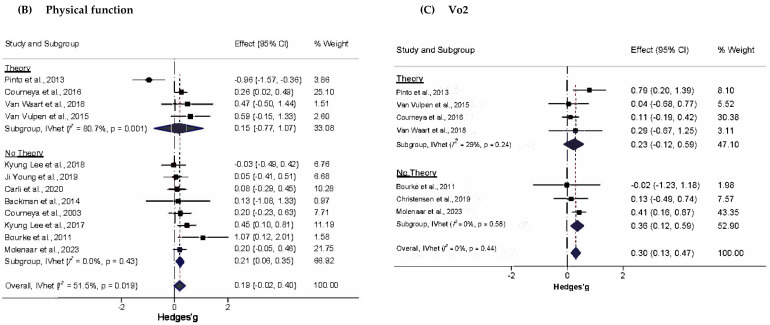
Forest plot for subgroup analyses (**A**) PA, (**B**) Physical function, and (**C**) VO_2_. The black-filled squares, sized according to the weight contributing to the overall effect, represent changes in physical function from each study, and the left and right extremes of each square represent the lower and upper 95% confidence intervals for those changes. The black diamond represents the pooled effect size change in physical function, and the left and right extremes of the diamond represent the pooled lower and upper 95% confidence intervals for these changes. The red dashed vertical line through the middle of the diamond represents the pooled mean effect, while the black dashed vertical line represents the zero (0) point. % Weight: Percentage weight of a particular study [65,66,67,68,69,70,71,72,73,74,75,76,77,78,79,80,81,82,83].

Subgroup analyses of changes in PA are shown in Figure 3A. For atheoretical and theoretical-based trials, effect sizes were 0.60 (95% CI, 0.15 to 1.04, Q = 53.9, *p* < 0.001, *I*^2^ = 83.3%, 95% CI, 70.7% to 90.5%), and 0.27 (95% CI, −0.08 to 0.62, Q = 13.8, *p* = 0.017, *I*^2^ = 63.9%, 95% CI, 12.7% to 85.1%), respectively. Z-tests indicated that changes in PA did not differ significantly between these subgroups (Zdiff = 1.43, *p* = 0.23). The magnitude of this difference (Diff = 0.33, 95% CI −0.12 to 0.78) supplemented these findings by suggesting that the true difference in changes in PA between atheoretical and theoretical works probably fell between −0.12 and 0.78, a range that includes the null value. (95% PI in Tables S8 and S9). This pattern was also similar to physical function, in which the difference in effect sizes between atheoretical (g = 0.21, 95% CI, 0.01 to 0.40, Q = 7.0, *p* = 0.32, *I*^2^ = 13.8, 95% CI, 0% to 74.8%) and theoretically based studies (g = 0.15, 95% CI, −0.77 to 1.07, Q = 15.6, *p* = 0.001, *I*^2^ = 15.5, 95% CI, 49.3% to 92.7%) was not statistically significant (Zdiff = 0.33, *p* = 0.23; Diff = 0.06, 95%CI −0.29 to 0.41) (Figure 3B). The difference in peak exercise VO2 for theoretical (g = 0.23, 95% CI, −0.12 to 0.59, Q = 4.23, *p* = 0.23, *I*^2^ = 29%, 95% CI, 0% to 77.5%) and atheoretical works (g = 0.36, 95% CI, 0.12 to 0.59, Q = 1.10, *p* = 0.58, *I*^2^ = 0%, 95% CI, 0% to 56.0%) was non-significant (Figure 3C).

#### 3.5.6. Meta-Regression

For meta-regression analyses (Table S10), positive significant associations with increases in PA were observed for higher risk of bias (“some concerns” and “high”), the reproducibility of control conditions, percentage of colon cancer survivors, risky behaviors, use of aerobic exercise, application of strength exercise, hospital or healthcare settings, intervention frequency and duration, and goal-setting (behavior) BCT. Negative significant associations with smaller increases in PA were observed for the use of theory, sample size, percentage of males, number of participants with stage T2 CRC, instructions on how to perform a behavior BCT, and Prompts/cues BCT.

## 4. Discussion

Physical activity interventions produced a statistically significant, moderate improvement in PA behavior among CRC survivors, with the exercise group showing greater improvement than the control group. While these results are encouraging, they need to be considered with respect to the statistically significant heterogeneity, the large amount of inconsistency, as well as small-study effects observed, all of which contributed to the overall low certainty of evidence based on our GRADE assessment. In contrast, no statistically significant improvements were observed for physical function or BMI, suggesting that increases in PA may not consistently translate into physiological improvements among CRC survivors.

When considering the overall risk of bias, the “some concerns” judgment arose strictly from insufficient details regarding the concealment of the allocation sequence, the awareness by intervention personnel and/or participants of their assigned intervention, and the lack of a pre-specified analysis plan. High-risk judgments were primarily driven by heavy reliance on subjective PA measures and inconsistent handling of missing data, both of which may have inflated or underestimated true intervention effects. A previous systematic review and meta-analysis showed that the validity of PA questionnaires was poor to moderate (regardless of the number of items). In contrast, reliability was moderate to good, and the quality of the examined studies was mostly fair to good, reinforcing the challenges of capturing accurate PA data [126].

Meta-regression analyses included several factors associated with changes in physical activity. These included study characteristics (sample size, risk of bias, use of theory), population characteristics (percentage of male participants, cancer site and stage), intervention characteristics (reproducibility, targeted risky behaviors, type and setting of prescribed exercise, exercise frequency and duration) and intervention components measured by specific behavior change techniques (BCTs) (goal setting, prompts/cues). Meta-regression suggested that changes in PA were associated with risk of bias, use of theory, intervention reproducibility, sample size, percentage of male participants, percentage of colon cancer survivors, CRC stage, risky behaviors, type and settings for prescribed exercises, frequency, and duration of prescribed exercises, and BCTs. The association between sample size and variation in effect size is well discussed in the literature [127]. However, the association between changes in PA and the percentage of male participants was novel. This suggests that certain types of interventions may not be particularly effective for male CRC survivors. Interestingly, the dual effect of BCTs on goal-setting (behavior) was associated with increases in PA. In contrast, instructions on how to perform a behavior and prompts/cues from BCTs were associated with an inverse effect. Previous research has also concluded that the goal-setting behavior BCT and instruction on how to perform a BCT behavior were, respectively, positively and negatively associated with changes in PA [19,128]. However, these associations are observational and should be interpreted as exploratory rather than causal findings.

Meta-regression results also suggested that the use of theory during interventions may be associated with a decrease in PA behavior. This finding was at odds with our subgroup analysis, which showed no statistically significant difference between theoretical and atheoretical trials. Based on our GRADE assessment, the certainty of evidence for both atheoretical and theory-based studies was low, reflecting methodological limitations, inconsistency across studies, and potential risk of bias rather than an absence of an effect. Although non-theoretical interventions appeared more effective in our synthesis, this finding should not be interpreted as evidence that theory-based approaches are inherently less effective. This difference most likely resulted from the various statistical approaches used [129]. Second, this may reflect limitations in how the theory was operationalized and implemented across the included studies. Many theoretical interventions applied theory superficially or did not translate theoretical principles into concrete components or strategies. Such partial or inconsistent use of theory has been shown to weaken intervention effectiveness and may explain the observed patterns more than the underlying theories themselves. The TCS evaluation indicated that most theoretical trials were merely informed by theory rather than attempting to test, apply comprehensively, or refine it; several key aspects of theory implementation were limited or absent. Future research should prioritize the comprehensive and transparent application of behavioral frameworks, including explicit mapping of constructs to intervention techniques and assessment of fidelity, to more accurately evaluate the added value of theory-driven design. Finally, meta-regression analyses are considered observational in nature [130] and thus should be tested in original randomized controlled trials.

### 4.1. Evaluation of Results Compared to Previous Systematic Reviews and Meta-Analyses

The observed improvements in PA were similar to those reported in previous reviews on this topic. For example, our previous systematic review with meta-analysis evaluating RCTs that used theory to design their interventions yielded a mean pooled estimate (Cohen’s d) of 0.26, similar to our current findings [21]. Another study examining mixed (atheoretical and theoretical) trials reported a standardized mean difference (SMD) of 0.80 (95% CI, 0.28 to 1.32; *I*^2^ = 79%) with inconsistency similar to our findings [22]. Regarding BCTs, a previous study reported that BCT prompts/cues were associated with changes in PA, albeit to a greater extent than in our study [19].

### 4.2. Implications for Research

Our findings suggest several areas of improvement for the reporting and conduct of future research on PA and CRC. First, there was a paucity of information regarding the ratio of colon to rectal cancer survivors, staging, regional or metastatic involvement, time since diagnosis, time since treatment completion, and comorbidities since diagnosis. Second, the stratification of certain variables did not abide by any standardization. The tiers used for categorization were determined by the authors, making it difficult to summarize data on variables such as income, education, and marital status. Third, a major goal of this study was to examine the association between changes in PA among CRC survivors by race and ethnicity. However, information on race/ethnicity was lacking and, when available, not well-described [37]. In the US, where efforts are ongoing to reduce racial health disparities, only one study included four race categories [69]. Notably, a lack of race data was reported for studies conducted in Canada despite its multicultural and multiethnic diversity [75,79,83]. There was also low representation of minorities (in studies where they were included), including Blacks. Although studies conducted in the Republic of Korea may have enrolled only Asian patients (as participants were recruited from a Seoul-based hospital), this was not explicitly stated. The former notwithstanding, it was encouraging that data, albeit in various forms, were available for race in the five trials conducted in the US [65,69,76,77,78]. However, only 43% of all U.S. RCTs reported race/ethnicity data [131]. A longitudinal study of all clinical trials conducted in the U.S. between 2000 and 2020 showed that the most represented races were Whites and Blacks, similar to our findings. However, minority underrepresentation compared to the U.S. population was significantly consistent and most acute for Hispanics and Asians [131]. The inadequate reporting of ethnic and racial information is commonplace, as is the underrepresentation of minorities, despite the almost 30-year-old National Institutes of Health (NIH) Revitalization Act of 1993, which established a federal mandate requiring all NIH-funded research to include minorities appropriately [132]. However, between 2013 and 2018, the proportion of racial minorities across all NIH-funded trials ranged from 19.8% in 2016 to 38% in 2017, and for ethnic minorities, from 8.1% in 2014 to 10.8% in 2016 [133]. Among NIH-funded cancer clinical trials, fewer than 2% focused primarily on minorities [132]. Certain barriers are often cited to explain minorities’ underrepresentation, including (a) the willingness to participate [133], (b) mistrust of the healthcare system [134], (c) social and economic factors [135], and (d) different race/ethnicity composition across the U.S. However, other research has shown that these factors are not associated with lower research participation [136,137]. Salient issues, however, seem to arise at the conceptual level of individual studies, including (1) the development of the research question, (2) patient and community engagement, (3) bias of the research team in its composition, training, and attitudes, (4) site selection and participant selection (the ability to recruit a representative sample of selected racial/ethnic groups based on the recruitment area), and (5) study protocols (consent processes and remuneration) [133].

Minority underrepresentation sustains health disparities, with cost estimates of additional life expectancy and working years to be gained from eliminating these disparities reported to be $11 trillion in the U.S. [133]. A recent report suggested several strategies to improve minority enrollment, including (1) having intention and agency to achieve representativeness, (2) establishing a foundation of trust with participants and community, (3) adopting a flexible approach to recruitment and data collection, (4) building a robust network with all stakeholders, (5) optimizing the study team to align with research goals, (6) increasing funding support and resources to communities, and (7) increasing representativeness at the scientific, professional and social level [133].

Inadequate reporting on location limited our ability to examine evidence across urban and rural environments, where the largest disparity is observed across the CRC continuum [138]. Studies have shown that CRC survivors living in lower-income zip codes have a greater incidence of CRC and are generally offered less aggressive treatment [139]. Along with having greater distances for care, rural cancer patients often incur higher treatment costs, are more likely to be uninsured, and hold jobs that offer less flexible leave policies compared to their urban counterparts [140]. This financial burden makes it more likely for rural cancer survivors to forgo additional medical care following treatment [140]. This may include adopting or maintaining newly prescribed behaviors. This particular burden and its ramifications may, as discussed earlier, affect their recruitment into intervention trials, thereby making it difficult to synthesize evidence across the important rural-urban divide. Importantly, a recent study identified significant rural-urban differences in cancer survivors’ PA, with urban survivors 2.6 times more likely to meet PA recommendations [141].

Inadequate reporting was found regarding the intervention’s intensity, compliance, and certain intervention characteristics. For example, while the trials mentioned the type of exercises conducted (aerobic, strength) [65,67,68,69,70,71,72,74,75,77,79,80,81,82,83], they did not provide sufficient details on the procedures or equipment employed [76,77,78,79,80]. Along those lines, while some studies mentioned the theory, it was not fully reported. From the lack of construct measurement to the lack of details on implementation procedures, the extent of theory use was generally weak, including, but not limited to, a rationale for the BCTs chosen. Future RCTs should adopt standardized reporting frameworks to enhance transparency, comparability, and reproducibility.

### 4.3. Clinical Implications

Our findings suggest that interventions to improve PA uptake may be effective and should be considered in survivorship care. However, limited minority representation and uncertain clinical significance underscore the need for clinicians to individualize exercise recommendations and to consider the barriers faced by underserved populations. The studies included in the analysis did not discuss their findings in terms of minimal clinically important differences (MCID). The absence of an established CRC-specific MCID for PA outcomes remains a major limitation. To illustrate, the MCID for deterioration in the 6-Minute Walk Distance (6MWD) among lung cancer patients has been estimated to range from 22 to 42 m, corresponding to approximately a 9.5% change [142]. To bridge these gaps, future investigations should deeply explore the potential consequences of a mere 5% increase in physical activity, focusing on pivotal cancer-related outcomes such as survival rates, quality of life, and recurrence rates. For instance, Jones et al. demonstrated that with every 50-m increase in 6MWD, there is a 13% reduction in the risk of death for patients with metastatic lung cancer [143]. Hence, endeavors to mitigate declines in functional capacity, such as exercise prescriptions, hold significance and could influence survival outcomes. By exploring these factors and qualifying the MID among colorectal cancer patients, a more comprehensive understanding of the impact of such incremental improvements in physical activity on colorectal cancer could be elucidated.

### 4.4. Study Limitations

The findings of the current study need to be interpreted with respect to the following potential limitations: (1) High heterogeneity and unexplained inconsistency substantially weaken confidence in pooled estimates (2) missing outcome data (quantitative data was requested for seven screened studies, yet only 43% of contacted authors responded to our requests); (3) Missing race/ethnicity data prevented our a priori subgroup analyses and limited equity-focused interpretations, (4) Limited and inconsistent theory implementation restricts conclusions about theory-driven effects, and (5) the potential for ecological fallacy, specifically Simpson’s paradox, since this was an aggregate data meta-analysis [144]. In addition, while new consensus guidelines now recommend 90 min of moderate PA per week for cancer survivors [145], we focused on the 150 min per week threshold because this is the threshold that selected studies have typically used. Finally, given the large number of meta-regression analyses, some statistically significant findings could have been mere chance.

## 5. Conclusions

Currently, low-certainty evidence suggests that RCT-based physical activity interventions may improve activity levels among CRC survivors. However, the certainty of this evidence remains low due to small sample sizes, high heterogeneity, and underrepresentation of racial and ethnic minorities. Improving reporting standards, ensuring diverse recruitment, and strengthening theory-based intervention designs are essential next steps to advance the effectiveness, equity, and generalizability of PA interventions in CRC survivorship.

## Figures and Tables

**Table 1:** Participants’ characteristics.

Author, Year	Country	Sex (% Males)	Race	Education	Income	BMI	Marital Status	Cancer: **1-** Site **2-** Stage	Comorbid Conditions	Treatment Received
Backman et al., 2014 [80]	Sweden	NI	NI	NI	NI	NI	NI	NI	Cardiovascular (39%)	CT: 52%
Bourke et al., 2011 [74]	UK	67%	NI	NI	NI	26.5	NI	Colon: 100% NI	NI	NI
Courneya et al., 2003 [83]	Canada	58.1%	NI	Completed university: 38.8%	Annual income > $40,000: 61.5%	NI	Married: 76.3%	Colon: 100% T3/4: 80.8%; N0: 59.5%; M: 4.6%	NI	Surgery: 33.7%, CT: 65.2%, RT: 20.7%; Surgery + RT: 1.1%; Surgery + CT: 45.6%; Surgery + RT + CT: 19.6%
Courneya et al., 2016 [75]	Canada & Australia	46%	NI	NI	NI	≤27.5: 42.6%; >27.5: 57.4%	NI	Colon: 100% T2: 12.3%; T3: 87.7%; N0: 12.8; N1+: 87.2%	NI	CT: 100%
Demark-Wahnefried et al., 2012 [65]	U.S.	44.7%	90% (NHW)	Any college education: 62%	NI	NI	NI	NI	NI	NI
Hawkes et al., 2013 [66]	Australia	54%	NI	Completed at least HS: 90.7%	Annual income; ≤$25,000: 24.1%; $25,001–$40,000: 24.9%; $40,001–$65,000: 12.9%; $65,000–$100,000: 13.9%; >$100,000: 15.1%	NI	Married: 77.1%	Colon: 67.3%; rectal: 32.7% Duke staging A: 18.3%; B: 28.9%; C: 22.7%; Unknown: 30.2%	Number of comorbidities; 0: 11.5%; 1 to 3: 62.7%; ≥4: 25.9%	Surgery: 96.1%; CT: 40.5%; RT: 13%
Ji Young et al., 2019 [67]	Republic of Korea	49.3%	All Asians	Completed College: 42.3%	>$3000/month: 46.5%	23.5	Married: 77.5%; Widowed: 8.5%; Divorced: 5.6%; Never married: 8.5%	Colon: 64.8%; Rectal: 35.2% T2: 46.5; T3: 53.5	NI	NI
Kyung Lee et al., 2017 [68]	Republic of Korea	48%	All Asians	Completed College: 48.8%	>$3000/month: 44.7%	23.5 (3.3)	Married: 81.3%; Widowed: 6.5%; Divorced: 4.1%; Single: 8.1%	Colon: 68.9%; Rectal: 31.1% T2: 46.7%; T3: 53.3%	NI	CT: 80.3%; CT + RT: 10.7%
Mayer et al., 2018 [76]	U.S.	48.2%	11% (NHB)	No HS: 4.9%; HS: 15.8%; College or higher: 79.3%	NI	(<18.5): 1.4%; 18.5–24.9: 19.7%; 25–29.9: 22.5%; >30: 25.7%	NI	NI T1: 24.3%; T2: 51.1%; T3: 24.6%	NI	Surgery: 51.7%; Surgery + CT: 48.2%
Pinto et al., 2013 [77]	U.S.	43.5%	2.2% (NHB)	Secondary education only: 24%; College and above: 76%	Annual income: <$39,999: 15.2%; $40,000–$59,999: 24%; >$60,000: 54.3%	28.7	Married: 67.4%; Divorced: 13%; Single: 15.2%	Colon: 56.5%; Rectal: 43.5% NI	NI	Surgery: 100%; RT: 43.5%; CT: 82.6%
Van Blarigan et al., 2019 [69]	U.S.	41%	2% (NHB), 12% (NH. Asian), 12% (AIAN)	College degree: 93%	NI	28.4 (5.9)	Married: 49%	Colon: 56%; Rectum: 44% T1: 20%; T2: 20%; T3: 59%; T4: 2%	NI	NI
Van Vulpen et al., 2016 * [70]	The Netherlands	64%	NI	Low: 12%; Medium: 33%; High: 52%; Unknown: 3%	NI	26.3	Couple: 70%; Single: 27.3%; Unknown: 2.7%	Colon: 100% NI	NI	Surgery: 100%; CT: 100%; RT: 91%
Van Waart et al., 2018 [71]	The Netherlands	39.1%	NI	HS and less: 52.2%; College: 47.8%	NI	24.1	Married: 69.6%; Not married: 30.4%	Colon: 100% T2: 13%; T3: 78.3%; T4: 8.7%	Unspecified: 65.2%	Surgery: 52.2%; RT: 4.4%; CT: 100%
Brown et al., 2018 [78]	USA	38%	12% (NHB), 5% (NH. Other)	High School or Less: 18%; Some College: 20%; College Degree & more: 62%;	NI	30.3	NI	Colon: 100% T1: 13%; T2: 36%; T3:51%;	HTN: 33%; HLD: 15%; Diabetes (type II): 13%; CVD: 10%	NI
Carli et al., 2020 [79]	Canada	47.3%	NI	NI	NI	26	NI	NI ≤1: 30%; 2: 30%; 3: 32%; 4: 6.4%	Diabetes (II): 36.4%; HTN: 64.5%; CVD: 32%; Atrial fibrillation: 12.7%; OSA: 9%; COPD: 10%; Arthritis: 34.5%; Dyslipidemia 49.1%; hypothyroidism: 20.9%; Asthma: 7.3%	Surgery: 100%; CT: 11.8%
Christensen et al., 2019 * [81]	Denmark	46%	NI	NI	NI	27.9 (4.2)	NI	Colon: 79%; Rectal: 21% 1: 26%; 2: 28%; 3: 46%	NI	CT: 54%
Kyung Lee et al., 2018 [72]	Republic of Korea	48.6%	All Asians	Completed College: 43.5%	NI	23.5 (3.2)	Married: 77.5%	Colon: 63.9%; Rectal: 36.1% T2: 45.8%; T3: 54.2%	NI	CT: 80.6%; CT + RT: 12.5%
Watson et al., 2015 [82]	UK	65.9%	NI	NI	NI	NI	NI	Colon: 39%; Rectum: 61% T0: 24.5; T1: 7.3%; T2: 29.3%; T3: 26.8%; T4: 12.2%; N0: 53.7%; N1: 14.6%; M0: 12.2%	NI	Surgery: 100%; RT: 12.2%; CT: 17.1%
Molenaar et al. 2023 * [73]	Canada, The Netherlands, Denmark, France, Italy, Spain	55%	NI	NI	NI	NI	NI	Colon 82.1%; Rectal: 17.9% T0: 5.6%; T1: 29%; T2: 30.3%; T3: 24.7%; T4: 3.2%	Diabetes: 18.3%; cardiovascular diseases: 51.4%; pulmonary diseases: 21.5%;	NI

*: not included in the meta-analysis due to insufficient information on PA; HS: high school; NHB: Non-Hispanic Blacks; NH: Non-Hispanics; AIAN: American Indian/Alaskan Natives; NHW: Non-Hispanic Whites; CT: Chemotherapy; RT: Radiation; NI: no information.

**Table 2:** Intervention characteristics and measured outcomes.

Author, Year	Exercise Intervention (L, F, I, D) **1-** Adherence **2-** Compliance **3-** Modality	Pre- and Post-PA by Intervention Arm, M (SD) **1-** PrMC **2-** PoMC **3-** PrMI **4-** PoMI	Other Outcomes
Backman et al., 2014 [80]	L:10, F: 1, I: moderate, D: 60 91% 74% In-person	15.6 (2.6) 12.5 (2.4) 14.3 (3.2) 14 (1.4)	NA
Bourke et al., 2011 [74]	L: 12, F: 1 in the first 6 weeks, then 2 in the second 6 weeks, I: 55–85% of age predicted max heart rate, D: 30 90% 94% In-person and exercise prescriptions	13 (6) 20 (10) 18 (10) 33 (17)	ΔPA, BMI
Courneya et al., 2003 [83]	L: 16, F: 3–5, I: 65–75% of age-predicted max heart rate, D: 20–30 NI NI Personalized exercise program	96.6 (126.4) 123.6 (182.7) 91.5 (148.4) 150.2 (109.7)	ΔPA
Courneya et al., 2016 [75]	L: 56, F: 2, I: NI, D: NI 68.2–82.8% NI Behavioral support sessions and Supervised exercise sessions	16.6 (19.2) 21.7 (20.2) 16.5 (22.4) 32.1 (30.7)	ΔPA, Aerobic fitness
Demark-Wahnefried et al., 2012 [65]	L: 48, F: 5–7, I: NI, D: 15–45 NI NI Telephone counseling, tailored workbook, quarterly newsletters, automated prompts	37.5 (3.2) 69 (7.8) 33.3 (2.9) 101.1 (101.1)	ΔPA
Hawkes et al., 2013 [66]	L: 24, F: 2, I: NI, D: NI 81.4 NI Telephone delivered, motivational postcards, quarterly newsletters	52.0 (112.5) 66.7 (139.2) 58.9 (132.9) 85.1 (197.9)	ΔPA, BMI
Ji Young et al., 2019 [67]	L: 12, F: 7, I: NI, D: 30 NI NI Telephone counseling, small group training sessions, and Home-based exercise program	117.5 (218.5) 133.8 (227.8) 97.0 (188.5) 332.6 (306.1)	ΔPA
Kyung Lee et al., 2017 [68]	L: 12, F: 7, I: NI, D: 30 NI NI Telephone counseling, small group training sessions, and Home-based exercise program	VPA 12.4 (74.4) 14.5 (75.7) 6.2 (33.1) 43.9 (102.4)	ΔPA, Upper body strength
		MPA 110.1 (197.4) 126.0 (195.6) 126.7 (215.8) 332.8 (307.9)	
Mayer et al., 2018 [76]	L: 24, F: 2, I: NI, D: NI NI NI Motivational message	13.2 (15.5) 25.5 (40.3) 14.3 (19.4) 31.4 (50)	ΔPA
Pinto et al., 2013 [77]	L: 12, F: 2, I: 76% of maximum heart rate, D: 10 C:52%; I:76% NI Telephone counseling, educational session	28.7(31.5) 97 (91) 37.6(72.5) 214 (125.5)	ΔPA, Aerobic fitness
Van Blarigan et al., 2019 [69]	L: 12, F: 2–3, I: NI, D: 30 NI NI Motivational text messages, educational materials	50.8 (20.7) 54.5 (24.9) 32.9 (17.9) 46.6 (48.4)	ΔPA
Van Vulpen et al., 2016 * [70]	L: 18, F: 2 supervised and two unsupervised, I: 45–75% maximum heart rate, D: 60 for supervised and 30 for unsupervised 89% NI Prescribed personalized exercises	540 (IQR:390–990) 56% of the participants reported more than 210 min/week 560 (IQR:240–990) 88% of the participants in the intervention group reported at least 210 min/week	ΔPA
Van Waart et al., 2018 [71]	L: 24, F: 5, I: 30, D: NI NI NI Tailored educational materials; general instructions	72.3 (53.4) 88.7 (35.5) 71.7 (75.2) 100.9 (80.5)	ΔPA, Upper and lower body strength
Brown et al., 2018 [78]	L: 24, F: NI, I: 50–70% maximum heart rate, D: NI LD:93%; HD: 89% NI Exercise prescription	85.4 (56.7) 114.8 (51.1)	ΔPA, BMI
		Low dose 131.6 (67.2) 291.2 (16.7)	
		High dose 109.9 (51.1) 413.7 (18)	
Carli et al., 2020 [79]	L: 4, F: 1, I: NI, D: 60 Prehab: 68%; Rehab: 38% NI Supervised training sessions	8.6 (16) 20.4 (17.5) 7.4 (2.2) 14.9 (2.7)	ΔPA
Christensen et al., 2019 * [81]	L: 12, F: NI, I: NI, D: NI NI NI Exercise prescriptions	The intervention group reported 1546 (23) minutes/week, and tracking data from the InterWalk application, on average, registered 119 (15) minutes/week	ΔPA
Kyung Lee et al., 2018 [72]	L: 6, F: 7, I: NI, D: 30 NI NI Telephone counseling, small group training sessions, and Home-based exercise program	VPA 0 6.6 (24.2) 0 20.1 (78.8)	ΔPA, BMI, Upper and lower body strength
		MPA 128.9 (187.0) 143.7 (174.0) 108.8 (196.6) 378.2 (297.6)	
Watson et al., 2015 [82]	L: 8–12, F: 1–2, I: NI, D: 60–90 62% 56%; 34% Educational counseling, Telephone counseling	29 (35.9) 36.7 (39) 21.2 (11.68) 25.8 (17.57)	ΔPA
Molenaar et al. 2023 * [73]	L:4; F:3; I: Aerobic (85–90%), Strength (65–70%); D:60. NI 77.20% In person	NI	ΔPhysical function, VO_2_

*: not included in the meta-analysis due to insufficient information on PA; L: length in weeks; F: frequency per week; I: intensity; D: duration in minutes; ΔPA: mean change in Physical activity uptake; M (SD) = mean (standard deviation); PrMC: pre-intervention physical activity (in mins)—control group; PoMC: post-intervention physical activity (in mins)—control group; PrMI: pre-intervention physical activity (in mins)—intervention group; PoMI: post-intervention physical activity (in mins)—intervention group; VPA: vigorous physical activity; MPA: moderate physical activity; NI: no information.

## Data Availability

No new data were created or analyzed in this study. Data sharing is not applicable to this article.

## Supplementary Materials

The following supporting information can be downloaded at: https://www.mdpi.com/article/10.3390/healthcare13243198/s1, Table S1: PRISMA 2020 Checklist; Table S2: Database search strategies (PubMed, Cochrane CENTRAL, CINAHL, PsycINFO, and Web of Science); Table S3: List of excluded studies with reasons for exclusion; Table S4: Extent of theory use across relevant articles; Table S5: Overview of the behavior change techniques (BCTs) used in included studies; Figure S1: Pooled risk of bias results using the Cochrane Risk of Bias Assessment v2.0 Instrument; Figure S2: Study level risk of bias; Table S6: GRADE results; Table S7: The 12-item Template for Intervention Description and Replication; Figure S3: Doi plot for changes in (A) PA, (B) Physical function, (C) VO2, (D) and BMI; Figure S4: Influence meta-analysis for changes in (A) PA, (B) Physical function, (C) VO2, (D) and BMI; Figure S5: Cumulative meta-analysis for changes in (A) PA, (B) Physical function, (C) VO2, (D) and BMI; Table S8: Magnitude of difference between atheoretical and theoretical subgroups, PIs (changes in PA); Table S9: Magnitude of difference between atheoretical and theoretical subgroups, PIs (changes in physical function); Table S10: Simple meta-regression results for changes in PA.

## References

[1] Cao W., Qin K., Li F., Chen W. (2024). Comparative Study of Cancer Profiles between 2020 and 2022 Using Global Cancer Statistics (GLOBOCAN). J. Natl. Cancer Cent..

[2] Sawicki T., Ruszkowska M., Danielewicz A., Niedźwiedzka E., Arłukowicz T., Przybyłowicz K.E. (2021). A Review of Colorectal Cancer in Terms of Epidemiology, Risk Factors, Development, Symptoms and Diagnosis. Cancers.

[3] Carethers J.M., Berger F.G., Boland C.R. (2021). Chapter Six—Racial and Ethnic Disparities in Colorectal Cancer Incidence and Mortality. Novel Approaches to Colorectal Cancer.

[4] Cormie P., Zopf E.M., Zhang X., Schmitz K.H. (2017). The Impact of Exercise on Cancer Mortality, Recurrence, and Treatment-Related Adverse Effects. Epidemiol. Rev..

[5] Qiu S., Jiang C., Zhou L. (2020). Physical Activity and Mortality in Patients with Colorectal Cancer: A Meta-Analysis of Prospective Cohort Studies. Eur. J. Cancer Prev..

[6] Hong J., Park J. (2021). Systematic Review: Recommendations of Levels of Physical Activity among Colorectal Cancer Patients (2010–2019). Int. J. Environ. Res. Public Health.

[7] Morishita S., Hamaue Y., Fukushima T., Tanaka T., Fu J.B., Nakano J. (2020). Effect of Exercise on Mortality and Recurrence in Patients with Cancer: A Systematic Review and Meta-Analysis. Integr. Cancer Ther..

[8] McGettigan M., Cardwell C.R., Cantwell M.M., Tully M.A. (2020). Physical Activity Interventions for Disease-Related Physical and Mental Health during and Following Treatment in People with Non-Advanced Colorectal Cancer. Cochrane Database Syst. Rev..

[9] Gao R., Yu T., Liu L., Bi J., Zhao H., Tao Y., Li F., Guo L. (2020). Exercise Intervention for Post-Treatment Colorectal Cancer Survivors: A Systematic Review and Meta-Analysis. J. Cancer Surviv..

[10] Rock C.L., Thomson C., Gansler T., Gapstur S.M., McCullough M.L., Patel A.V., Andrews K.S., Bandera E.V., Spees C.K., Robien K. (2020). American Cancer Society Guideline for Diet and Physical Activity for Cancer Prevention. CA Cancer J. Clin..

[11] Ligibel J.A., Denlinger C.S. (2013). New NCCN Guidelines^®^ for Survivorship Care. J. Natl. Compr. Cancer Netw..

[12] Meyerhardt J.A., Mangu P.B., Flynn P.J., Korde L., Loprinzi C.L., Minsky B.D., Petrelli N.J., Ryan K., Schrag D.H., Wong S.L. (2013). Follow-up Care, Surveillance Protocol, and Secondary Prevention Measures for Survivors of Colorectal Cancer: American Society of Clinical Oncology Clinical Practice Guideline Endorsement. J. Clin. Oncol..

[13] Troeschel A.N., Leach C.R., Shuval K., Stein K.D., Patel A.V. (2018). Physical Activity in Cancer Survivors during “Re-Entry” Following Cancer Treatment. Prev. Chronic Dis..

[14] Bøhn S.K.H., Lie H.C., Reinertsen K.V., Fosså S.D., Haugnes H.S., Kiserud C.E., Loge J.H., Wisløff T., Thorsen L. (2021). Lifestyle among Long-Term Survivors of Cancers in Young Adulthood. Support. Care Cancer.

[15] Blanchard C.M., Courneya K.S., Stein K., American Cancer Society’s SCS-II (2008). Cancer Survivors’ Adherence to Lifestyle Behavior Recommendations and Associations with Health-Related Quality of Life: Results from the American Cancer Society’s SCS-II. J. Clin. Oncol..

[16] Ray A.D., Masucci Twarozek A., Williams B.T., Erwin D.O., Underwood W., Mahoney M.C. (2018). Exercise in African American and White Colorectal Cancer Survivors: A Mixed-Methods Approach. Rehabil. Oncol..

[17] Stacey F.G., James E.L., Chapman K., Courneya K.S., Lubans D.R. (2015). A Systematic Review and Meta-Analysis of Social Cognitive Theory-Based Physical Activity and/or Nutrition Behavior Change Interventions for Cancer Survivors. J. Cancer Surviv..

[18] Goode A.D., Lawler S.P., Brakenridge C.L., Reeves M.M., Eakin E.G. (2015). Telephone, Print, and Web-Based Interventions for Physical Activity, Diet, and Weight Control among Cancer Survivors: A Systematic Review. J. Cancer Surviv..

[19] Finne E., Glausch M., Exner A.K., Sauzet O., Stölzel F., Seidel N. (2018). Behavior Change Techniques for Increasing Physical Activity in Cancer Survivors: A Systematic Review and Meta-Analysis of Randomized Controlled Trials. Cancer Manag. Res..

[20] Coughlin S.S., Caplan L.S., Stone R. (2020). Use of Consumer Wearable Devices to Promote Physical Activity among Breast, Prostate, and Colorectal Cancer Survivors: A Review of Health Intervention Studies. J. Cancer Surviv..

[21] Mbous Y.P., Patel J., Kelly K.M. (2020). A Systematic Review and Meta-Analysis of Physical Activity Interventions among Colorectal Cancer Survivors. Transl. Behav. Med..

[22] Jung Y., Chung J., Son H. (2021). Physical Activity Interventions for Colorectal Cancer Survivors. Cancer Nurs..

[23] Spineli L.M., Pandis N. (2020). Prediction Interval in Random-Effects Meta-Analysis. Am. J. Orthod. Dentofac. Orthop..

[24] Painter J.E., Borba C.P.C., Hynes M., Mays D., Glanz K. (2008). The Use of Theory in Health Behavior Research from 2000 to 2005: A Systematic Review. Ann. Behav. Med..

[25] Prestwich A., Sniehotta F.F., Whittington C., Dombrowski S.U., Rogers L., Michie S. (2014). Does Theory Influence the Effectiveness of Health Behavior Interventions? Meta-Analysis. Health Psychol..

[26] Prestwich A., Webb T.L., Conner M. (2015). Using Theory to Develop and Test Interventions to Promote Changes in Health Behaviour: Evidence, Issues, and Recommendations. Curr. Opin. Psychol..

[27] Higgins J.P.T., Thomas J., Chandler J., Cumpston M., Li T., Page M.J., Welch V.A. (2020). Cochrane Handbook for Systematic Reviews of Interventions.

[28] Page M.J., Moher D., Bossuyt P.M., Boutron I., Hoffmann T.C., Mulrow C.D., Shamseer L., Tetzlaff J.M., Akl E.A., Brennan S.E. (2021). PRISMA 2020 Explanation and Elaboration: Updated Guidance and Exemplars for Reporting Systematic Reviews. BMJ.

[29] Rethlefsen M.L., Kirtley S., Waffenschmidt S., Ayala A.P., Moher D., Page M.J., Koffel J.B., Blunt H., Brigham T., Chang S. (2021). PRISMA-S: An Extension to the PRISMA Statement for Reporting Literature Searches in Systematic Reviews. Syst. Rev..

[30] Mbous Y.P.V., Mohamed R., Kelley G.A., Kelly K.M. (2021). Interventions to Improve Physical Activity in Colorectal Cancer Survivors: Protocol for a Systematic Review and Meta-Analysis of Randomized Controlled Trials. J. Adv. Nurs..

[31] Lee E., Dobbins M., DeCorby K., McRae L., Tirilis D., Husson H. (2012). An Optimal Search Filter for Retrieving Systematic Reviews and Meta-Analyses. BMC Med. Res. Methodol..

[32] Gwet K.L. (2008). Computing Inter-Rater Reliability and Its Variance in the Presence of High Agreement. Br. J. Math. Stat. Psychol..

[33] Kelley G.A., Kelley K.S., Pate R.R. (2017). Exercise and Adiposity in Overweight and Obese Children and Adolescents: Protocol for a Systematic Review and Network Meta-Analysis of Randomised Trials. BMJ Open.

[34] Kelley G.A., Kelley K.S., Pate R.R. (2017). Exercise and BMI Z-Score in Overweight and Obese Children and Adolescents: A Systematic Review and Network Meta-Analysis of Randomized Trials. J. Evid. Based Med..

[35] Nerenz D.R., McFadden B., Ulmer C. (2009). Race, Ethnicity, and Language Data: Standardization for Health Care Quality Improvement.

[36] US Census Bureau Race. https://www.census.gov/topics/population/race.html.

[37] Office of Management and Budget (1997). Revisions to the Standards for the Classification of Federal Data on Race and Ethnicity. Fed. Regist..

[38] Herring L.Y., Stevinson C., Davies M.J., Biddle S.J.H., Sutton C., Bowrey D., Carter P. (2016). Changes in Physical Activity Behaviour and Physical Function after Bariatric Surgery: A Systematic Review and Meta-Analysis. Obes. Rev..

[39] Flaubert J.L., Spicer C.M., Volberding P.A., National Academies of Sciences, Engineering, and Medicine (2019). Selected Instruments for Assessment of Physical Functional Abilities Relevant to Work Requirements. Functional Assessment for Adults with Disabilities.

[40] Maginador G., Lixandrão M.E., Bortolozo H.I., Vechin F.C., Sarian L.O., Derchain S., Telles G.D., Zopf E., Ugrinowitsch C., Conceição M.S. (2020). Aerobic Exercise-Induced Changes in Cardiorespiratory Fitness in Breast Cancer Patients Receiving Chemotherapy: A Systematic Review and Meta-Analysis. Cancers.

[41] Legg H.S., Spindor J., Dziendzielowski R., Sharkey S., Lanovaz J.L., Farthing J.P., Arnold C.M. (2020). The Reliability and Validity of Novel Clinical Strength Measures of the Upper Body in Older Adults. Hand Ther..

[42] Jones C.J., Rikli R.E., Beam W.C. (1999). A 30-s Chair-Stand Test as a Measure of Lower Body Strength in Community-Residing Older Adults. Res. Q. Exerc. Sport.

[43] Schaubert K.L., Bohannon R.W. (2005). Reliability and Validity of Three Strength Measures Obtained From Community Dwelling Elderly Persons. J. Strength Cond. Res..

[44] Sterne J.A.C., Savović J., Page M.J., Elbers R.G., Blencowe N.S., Boutron I., Cates C.J., Cheng H.Y., Corbett M.S., Eldridge S.M. (2019). RoB 2: A Revised Tool for Assessing Risk of Bias in Randomised Trials. BMJ.

[45] Schünemann H., Brożek J., Guyatt G., Oxman A. (2013). GRADE Handbook for Grading Quality of Evidence and Strength of Recommendations.

[46] Hoffmann T.C., Glasziou P.P., Boutron I., Milne R., Perera R., Moher D., Altman D.G., Barbour V., MacDonald H., Johnston M. (2016). Better Reporting of Interventions: Template for Intervention Description and Replication (TIDieR) Checklist and Guide. Gesundheitswesen.

[47] Michie S., Prestwich A. (2010). Are Interventions Theory-Based? Development of a Theory Coding Scheme. Health Psychol..

[48] Michie S., Richardson M., Johnston M., Abraham C., Francis J., Hardeman W., Eccles M.P., Cane J., Wood C.E. (2013). The Behavior Change Technique Taxonomy (v1) of 93 Hierarchically Clustered Techniques: Building an International Consensus for the Reporting of Behavior Change Interventions. Ann. Behav. Med..

[49] Freeman P.R., Hedges L.V., Olkin I. (1986). Statistical Methods for Meta-Analysis. Biometrics.

[50] Follmann D., Elliott P., Suh I., Cutler J. (1992). Variance Imputation for Overviews of Clinical Trials with Continuous Response. J. Clin. Epidemiol..

[51] Doi S.A.R., Barendregt J.J., Khan S., Thalib L., Williams G.M. (2015). Advances in the Meta-Analysis of Heterogeneous Clinical Trials I: The Inverse Variance Heterogeneity Model. Contemp. Clin. Trials.

[52] Doi S.A.R. (2022). Examining How Meta-analytic Methods Perform. Res. Synth. Methods.

[53] Johnson K., Hayen A., Lassere M.N.D., Mengersen K. (2020). Bias, Coverage, and Asymptotic Behaviour of Random Effects Meta-Analysis: A Clinically Driven Simulation Study. JBI Evid. Implement..

[54] Kelley G.A., Kelley K.S. (2023). Evolution of Statistical Models for Meta-Analysis and Implications for Best Practice. Curr. Opin. Epidemiol. Public Health.

[55] Cochran W.G. (1954). The Combination of Estimates from Different Experiments. Biometrics.

[56] Higgins J.P.T., Thompson S.G., Deeks J.J., Altman D.G. (2003). Measuring Inconsistency in Meta-Analyses. Br. Med. J..

[57] Doi S.A.R., Furuya-Kanamori L. (2020). Selecting the Best Meta-Analytic Estimator for Evidence-Based Practice: A Simulation Study. Int. J. Evid. Based Healthc..

[58] Doi S.A.R., Furuya-Kanamori L., Thalib L., Barendregt J.J. (2017). Meta-Analysis in Evidence-Based Healthcare: A Paradigm Shift Away from Random Effects Is Overdue. JBI Evid. Implement..

[59] National Cancer Institute Statistics, Graphs and Definitions. https://www.cancer.gov/about-cancer/understanding/statistics.

[60] Kelly K.M., Shah N., Shedlosky-Shoemaker R., Porter K., Agnese D. (2011). Living Post Treatment: Definitions of Those with History and No History of Cancer. J. Cancer Surviv..

[61] Lau J., Schmid C.H., Chalmers T.C. (1995). Cumulative Meta-Analysis of Clinical Trials Builds Evidence for Exemplary Medical Care. J. Clin. Epidemiol..

[62] Deeks J.J., Higgins J.P.T., Altman D.G., Group C.S.M. (2019). Analysing Data and Undertaking Meta-analyses. Cochrane Handbook for Systematic Reviews of Interventions.

[63] Rothman K.J. (1990). No Adjustments Are Needed for Multiple Comparisons. Epidemiology.

[64] Fu R., Gartlehner G., Grant M., Shamliyan T., Sedrakyan A., Wilt T.J., Griffith L., Oremus M., Raina P., Ismaila A. (2011). Conducting Quantitative Synthesis When Comparing Medical Interventions: AHRQ and the Effective Health Care Program. J. Clin. Epidemiol..

[65] Demark-Wahnefried W., Morey M.C., Sloane R., Snyder D.C., Miller P.E., Hartman T.J., Cohen H.J. (2012). Reach out to Enhance Wellness Home-Based Diet-Exercise Intervention Promotes Reproducible and Sustainable Long-Term Improvements in Health Behaviors, Body Weight, and Physical Functioning in Older, Overweight/Obese Cancer Survivors. J. Clin. Oncol..

[66] Hawkes A.L., Chambers S.K., Pakenham K.I., Patrao T.A., Baade P.D., Lynch B.M., Aitken J.F., Meng X., Courneya K.S. (2013). Effects of a Telephone-Delivered Multiple Health Behavior Change Intervention (CanChange) on Health and Behavioral Outcomes in Survivors of Colorectal Cancer: A Randomized Controlled Trial. J. Clin. Oncol..

[67] Kim J.Y., Lee M.K., Lee D.H., Kang D.W., Min J.H., Lee J.W., Chu S.H., Cho M.S., Kim N.K., Jeon J.Y. (2019). Effects of a 12-Week Home-Based Exercise Program on Quality of Life, Psychological Health, and the Level of Physical Activity in Colorectal Cancer Survivors: A Randomized Controlled Trial. Support. Care Cancer.

[68] Lee M.K., Kim J.-Y., Kim D.-I., Kang D.-W., Park J.-H., Ahn K.-Y., In Yang H., Lee D.H., Roh Y.H., Lee J.-W. (2017). Effect of Home-Based Exercise Intervention on Fasting Insulin and Adipocytokines in Colorectal Cancer Survivors: A Randomized Controlled Trial. Metabolism.

[69] Van Blarigan E.L., Chan H., Van Loon K., Kenfield S.A., Chan J.M., Mitchell E., Zhang L., Paciorek A., Joseph G., Laffan A. (2019). Self-Monitoring and Reminder Text Messages to Increase Physical Activity in Colorectal Cancer Survivors (Smart Pace): A Pilot Randomized Controlled Trial. BMC Cancer.

[70] Van Vulpen J.K., Velthuis M.J., Steins Bisschop C.N., Travier N., Van Den Buijs B.J.W., Backx F.J.G., Los M., Erdkamp F.L.G., Bloemendal H.J., Koopman M. (2016). Effects of an Exercise Program in Colon Cancer Patients Undergoing Chemotherapy. Med. Sci. Sports Exerc..

[71] Van70 Waart H., Stuiver M.M., van Harten W.H., Geleijn E., de Maaker-Berkhof M., Schrama J., Geenen M.M., Terwogt J.M.M., van den Heiligenberg S.M., Hellendoorn-van Vreeswijk J.A. (2018). Recruitment to and Pilot Results of the PACES Randomized Trial of Physical Exercise during Adjuvant Chemotherapy for Colon Cancer. Int. J. Color. Dis..

[72] Lee M.K., Kim N.K., Jeon J.Y. (2018). Effect of the 6-Week Home-Based Exercise Program on Physical Activity Level and Physical Fitness in Colorectal Cancer Survivors: A Randomized Controlled Pilot Study. PLoS ONE.

[73] Molenaar C.J.L., Minnella E.M., Coca-Martinez M., Ten Cate D.W.G., Regis M., Awasthi R., Martínez-Palli G., López-Baamonde M., Sebio-Garcia R., Feo C.V. (2023). Effect of Multimodal Prehabilitation on Reducing Postoperative Complications and Enhancing Functional Capacity Following Colorectal Cancer Surgery: The PREHAB Randomized Clinical Trial. JAMA Surg..

[74] Bourke L., Thompson G., Gibson D.J., Daley A., Crank H., Adam I., Shorthouse A., Saxton J. (2011). Pragmatic Lifestyle Intervention in Patients Recovering from Colon Cancer: A Randomized Controlled Pilot Study. Arch. Phys. Med. Rehabil..

[75] Courneya K.S., Vardy J.L., O’Callaghan C.J., Friedenreich C.M., Campbell K.L., Prapavessis H., Crawford J.J., O’Brien P., Dhillon H.M., Jonker D.J. (2016). Effects of a Structured Exercise Program on Physical Activity and Fitness in Colon Cancer Survivors: One Year Feasibility Results from the CHALLENGE Trial. Cancer Epidemiol. Biomark. Prev..

[76] Mayer D.K., Landucci G., Awoyinka L., Atwood A.K., Carmack C.L., Demark-Wahnefried W., McTavish F., Gustafson D.H. (2018). SurvivorCHESS to Increase Physical Activity in Colon Cancer Survivors: Can We Get Them Moving?. J. Cancer Surviv..

[77] Pinto B.M., Papandonatos G.D., Goldstein M.G., Marcus B.H., Farrell N. (2013). Home-Based Physical Activity Intervention for Colorectal Cancer Survivors. Psychooncology.

[78] Brown J.C., Troxel A.B., Ky B., Damjanov N., Zemel B.S., Rickels M.R., Rhim A.D., Rustgi A.K., Courneya K.S., Schmitz K.H. (2018). Dose-Response Effects of Aerobic Exercise Among Colon Cancer Survivors: A Randomized Phase II Trial. Clin. Color. Cancer.

[79] Carli F., Bousquet-Dion G., Awasthi R., Elsherbini N., Liberman S., Boutros M., Stein B., Charlebois P., Ghitulescu G., Morin N. (2020). Effect of Multimodal Prehabilitation vs. Postoperative Rehabilitation on 30-Day Postoperative Complications for Frail Patients Undergoing Resection of Colorectal Cancer: A Randomized Clinical Trial. JAMA Surg..

[80] Backman M., Wengstrom Y., Johansson B., Skoldengen I., Borjesson S., Tarnbro S., Berglund A. (2014). A Randomized Pilot Study with Daily Walking during Adjuvant Chemotherapy for Patients with Breast and Colorectal Cancer. Acta Oncol..

[81] Christensen J.F., Sundberg A., Osterkamp J., Thorsen-Streit S., Nielsen A.B., Olsen C.K., Djurhuus S.S., Simonsen C., Schauer T., Ellingsgaard H. (2019). Interval Walking Improves Glycemic Control and Body Composition After Cancer Treatment: A Randomized Controlled Trial. J. Clin. Endocrinol. Metab..

[82] Watson A.J.M., Hubbard G., Munro J., Adams R. (2015). Is Use of Cardiac Rehabilitation an Acceptable and Feasible Rehabilitation Model for Patients with Colorectal Cancer and Is a Randomised Trial of This Intervention Also Acceptable and Feasible?. Gut.

[83] Courneya K.S., Friedenreich C.M., Quinney H.A., Fields A.L.A., Jones L.W., Fairey A.S. (2003). A Randomized Trial of Exercise and Quality of Life in Colorectal Cancer Survivors. Eur. J. Cancer Care.

[84] Cadmus-Bertram L., Tevaarwerk A.J., Sesto M.E., Gangnon R., Van Remortel B., Date P. (2019). Building a Physical Activity Intervention into Clinical Care for Breast and Colorectal Cancer Survivors in Wisconsin: A Randomized Controlled Pilot Trial. J. Cancer Surviv..

[85] Greenlee H., Lew D.L., Hershman D.L., Newman V.A., Hansen L., Hartman S.J., Korner J., Shi Z., Sardo Molmenti C.L., Sayegh A. (2018). Phase II Feasibility Study of a Weight Loss Intervention in Female Breast and Colorectal Cancer Survivors (SWOG S1008). Obes. Silver Spring.

[86] Maxwell-Smith C., Hince D., Cohen P.A., Bulsara M.K., Boyle T., Platell C., Tan P., Levitt M., Salama P., Tan J. (2019). A Randomized Controlled Trial of WATAAP to Promote Physical Activity in Colorectal and Endometrial Cancer Survivors. Psychooncology.

[87] Park J.-H., Lee J., Oh M., Park H., Chae J., Kim D.-I., Lee M.K., Yoon Y.J., Lee C.W., Park S. (2015). The Effect of Oncologists’ Exercise Recommendations on the Level of Exercise and Quality of Life in Survivors of Breast and Colorectal Cancer: A Randomized Controlled Trial. Cancer.

[88] Ungar N., Sieverding M., Weidner G., Ulrich C.M., Wiskemann J. (2016). A Self-Regulation-Based Intervention to Increase Physical Activity in Cancer Patients. Psychol. Health Med..

[89] Yun Y.H., Kim Y.A., Lee M.K., Sim J.A., Nam B.-H., Kim S., Lee E.S., Noh D.-Y., Lim J.-Y., Kim S. (2017). A Randomized Controlled Trial of Physical Activity, Dietary Habit, and Distress Management with the Leadership and Coaching for Health (LEACH) Program for Disease-Free Cancer Survivors. BMC Cancer.

[90] Rees-Punia E., Leach C.R., Westmaas J.L., Dempsey L.F., Roberts A.M., Nocera J.R., Patel A.V. (2022). Pilot Randomized Controlled Trial of Feasibility, Acceptability, and Preliminary Efficacy of a Web-Based Physical Activity and Sedentary Time Intervention for Survivors of Physical Inactivity-Related Cancers. Int. J. Behav. Med..

[91] Demmelmaier I., Brooke H.L., Henriksson A., Mazzoni A.S., Bjørke A.C.H., Igelström H., Ax A.K., Sjövall K., Hellbom M., Pingel R. (2021). Does Exercise Intensity Matter for Fatigue during (Neo-)Adjuvant Cancer Treatment? The Phys-Can Randomized Clinical Trial. Scand. J. Med. Sci. Sports.

[92] Bousquet-Dion G., Awasthi R., Loiselle S.-E., Minnella E.M., Agnihotram R.V., Bergdahl A., Carli F., Scheede-Bergdahl C. (2018). Evaluation of Supervised Multimodal Prehabilitation Programme in Cancer Patients Undergoing Colorectal Resection: A Randomized Control Trial. Acta Oncol..

[93] Golsteijn R.H.J., Bolman C., Peels D.A., Volders E., de Vries H., Lechner L. (2017). A Web-Based and Print-Based Computer-Tailored Physical Activity Intervention for Prostate and Colorectal Cancer Survivors: A Comparison of User Characteristics and Intervention Use. J. Med. Internet Res..

[94] Hwang S.H., Kang D.W., Lee M.K., Byeon J.Y., Park H., Park D.H., Kim K.C., Lee S.T., Chu S.H., Kim N.K. (2022). Changes in DNA Methylation after 6-Week Exercise Training in Colorectal Cancer Survivors: A Preliminary Study. Asia Pac. J. Clin. Oncol..

[95] Campbell M.K., Carr C., Devellis B., Switzer B., Biddle A., Amamoo M.A., Walsh J., Zhou B., Sandler R. (2009). A Randomized Trial of Tailoring and Motivational Interviewing to Promote Fruit and Vegetable Consumption for Cancer Prevention and Control. Ann. Behav. Med..

[96] Ligibel J.A., Meyerhardt J., Pierce J.P., Najita J., Shockro L., Campbell N., Newman V.A., Barbier L., Hacker E., Wood M. (2012). Impact of a Telephone-Based Physical Activity Intervention upon Exercise Behaviors and Fitness in Cancer Survivors Enrolled in a Cooperative Group Setting. Breast Cancer Res. Treat..

[97] Agnew H., Kitson S., Crosbie E.J. (2023). Interventions for Weight Reduction in Obesity to Improve Survival in Women with Endometrial Cancer. Cochrane Database Syst. Rev..

[98] Andersson M., Egenvall M., Danielsson J., Thorell A., Sturesson C., Soop M., Nygren-Bonnier M., Rydwik E. (2023). CANOPTIPHYS Study Protocol: Optimising PHYSical Function before CANcer Surgery: Effects of Pre-Operative Optimisation on Complications and Physical Function after Gastrointestinal Cancer Surgery in Older People at Risk-a Multicentre, Randomised, Parallel-Group Study. Trials.

[99] Hirschey R., Lipkus I., Jones L., Mantyh C., Sloane R., Demark-Wahnefried W. (2016). Message Framing and Physical Activity Promotion in Colorectal Cancer Survivors. Oncol. Nurs. Forum.

[100] Cramer H., Pokhrel B., Fester C., Meier B., Gass F., Lauche R., Eggleston B., Walz M., Michalsen A., Kunz R. (2016). A Randomized Controlled Bicenter Trial of Yoga for Patients with Colorectal Cancer. Psychooncology.

[101] Forbes C.C., Blanchard C.M., Mummery W.K., Courneya K.S. (2017). A Pilot Study on the Motivational Effects of an Internet-Delivered Physical Activity Behaviour Change Programme in Nova Scotian Cancer Survivors. Psychol. Health.

[102] Guercio B.J., Zhang S., Ou F.-S., Venook A.P., Niedzwiecki D., Lenz H.-J., Innocenti F., O’Neil B.H., Shaw J.E., Polite B.N. (2019). Associations of Physical Activity With Survival and Progression in Metastatic Colorectal Cancer: Results From Cancer and Leukemia Group B (Alliance)/SWOG 80405. J. Clin. Oncol..

[103] Møller T., Lillelund C., Andersen C., Ejlertsen B., Nørgaard L., Christensen K.B., Vadstrup E., Diderichsen F., Hendriksen C., Bloomquist K. (2013). At Cancer Diagnosis: A ‘Window of Opportunity’ for Behavioural Change towards Physical Activity. A Randomised Feasibility Study in Patients with Colon and Breast Cancer. BMJ Open.

[104] Sohl S.J., Danhauer S.C., Birdee G.S., Nicklas B.J., Yacoub G., Aklilu M., Avis N.E. (2016). A Brief Yoga Intervention Implemented during Chemotherapy: A Randomized Controlled Pilot Study. Complement Ther. Med..

[105] López-Rodríguez-Arias F., Sánchez-Guillén L., Aranaz-Ostáriz V., Triguero-Cánovas D., Lario-Pérez S., Barber-Valles X., Lacueva F.J., Ramirez J.M., Arroyo A. (2021). Effect of Home-Based Prehabilitation in an Enhanced Recovery after Surgery Program for Patients Undergoing Colorectal Cancer Surgery during the COVID-19 Pandemic. Support. Care Cancer.

[106] Morielli A.R., Usmani N., Boulé N.G., Severin D., Tankel K., Joseph K., Nijjar T., Fairchild A., Courneya K.S. (2021). Feasibility, Safety, and Preliminary Efficacy of Exercise During and After Neoadjuvant Rectal Cancer Treatment: A Phase II Randomized Controlled Trial. Clin. Color. Cancer.

[107] Onerup A., Andersson J., Angenete E., Bock D., Börjesson M., Ehrencrona C., Fagevik Olsén M., Larsson P.A., De La Croix H., Wedin A. (2022). Effect of Short-Term Homebased Pre- and Postoperative Exercise on Recovery after Colorectal Cancer Surgery (PHYSSURG-C): A Randomized Clinical Trial. Ann. Surg..

[108] Ozhanli Y., Akyuz N. (2021). The Effect of Progressive Relaxation Exercise on Physiological Parameters, Pain and Anxiety Levels of Patients Undergoing Colorectal Cancer Surgery: A Randomized Controlled Study. J. Perianesthesia Nurs..

[109] Peng L.H., Wang W.J., Chen J., Jin J.Y., Min S., Qin P.P. (2021). Implementation of the Pre-Operative Rehabilitation Recovery Protocol and Its Effect on the Quality of Recovery after Colorectal Surgeries. Chin. Med. J..

[110] Sohl S.J., Tooze J.A., Johnson E.N., Ridner S.H., Rothman R.L., Lima C.R., Ansley K.C., Wheeler A., Nicklas B., Avis N.E. (2022). A Randomized Controlled Pilot Study of Yoga Skills Training Versus an Attention Control Delivered During Chemotherapy Administration. J. Pain Symptom Manag..

[111] Taha A., Taha-Mehlitz S., Staartjes V.E., Lunger F., Gloor S., Unger I., Mungo G., Tschuor C., Breitenstein S., Gingert C. (2021). Association of a Prehabilitation Program with Anxiety and Depression before Colorectal Surgery: A Post Hoc Analysis of the PERACS Randomized Controlled Trial. Langenbecks Arch. Surg..

[112] Zopf E.M., Schulz H., Poeschko J., Aschenbroich K., Wilhelm T., Eypasch E., Kleimann E., Severin K., Benz J., Liu E. (2022). Effects of Supervised Aerobic Exercise on Cardiorespiratory Fitness and Patient-Reported Health Outcomes in Colorectal Cancer Patients Undergoing Adjuvant Chemotherapy—A Pilot Study. Support. Care Cancer.

[113] Howell D., Pond G.R., Bryant-Lukosius D., Powis M., McGowan P.T., Makuwaza T., Kukreti V., Rask S., Hack S., Krzyzanowska M.K. (2023). Feasibility and Effectiveness of Self-Management Education and Coaching on Patient Activation for Managing Cancer Treatment Toxicities. J. Natl. Compr. Canc Netw..

[114] Li C., Li Z.-Y., Lu Q., Zhou Y.-J., Qin X.-Y., Wu A.-W., Pang D. (2023). The Effectiveness of a Self-Management Program of Bowel Dysfunction in Patients with Mid and Low Rectal Cancer After Sphincter-Preserving Surgery: A Pilot Randomized Controlled Trial. Cancer Nurs..

[115] Min J., An K.-Y., Park H., Cho W., Jung H.J., Chu S.H., Cho M., Yang S.Y., Jeon J.Y., Kim N.K. (2023). Postoperative Inpatient Exercise Facilitates Recovery after Laparoscopic Surgery in Colorectal Cancer Patients: A Randomized Controlled Trial. BMC Gastroenterol..

[116] Sepucha K.R., Valentine K.D., Atlas S.J., Chang Y., Fairfield K.M., Ha J., Leavitt L., Lee V., Percac-Lima S., Richter J.M. (2023). Getting Patients Back for Routine Colorectal Cancer Screening: Randomized Controlled Trial of a Shared Decision-Making Intervention. Cancer Med..

[117] Sepucha K., Han P.K.J., Chang Y., Atlas S.J., Korsen N., Leavitt L., Lee V., Percac-Lima S., Mancini B., Richter J. (2023). Promoting Informed Decisions About Colorectal Cancer Screening in Older Adults (PRIMED Study): A Physician Cluster Randomized Trial. J. Gen. Intern. Med..

[118] Snowden A., Young J., Roberge D., Schipani S., Murray E., Richard C., Lussier M.-T., White C. (2023). Holistic Needs Assessment in Outpatient Cancer Care: A Randomised Controlled Trial. BMJ Open.

[119] Lynch B.M., Courneya K.S., Sethi P., Patrao T.A., Hawkes A.L. (2014). A Randomized Controlled Trial of a Multiple Health Behavior Change Intervention Delivered to Colorectal Cancer Survivors: Effects on Sedentary Behavior. Cancer.

[120] Yun Y.H., Lim C.I., Lee E.S., Kim Y.T., Shin K.H., Park K.J., Jeong S., Ryu K.W., Han W., Jung K.H. (2020). Efficacy of Health Coaching and a Web-Based Program on Physical Activity, Weight, and Distress Management among Cancer Survivors: A Multi-Centered Randomised Controlled Trial. Psychooncology.

[121] Mazzoni A.S., Brooke H.L., Berntsen S., Nordin K., Demmelmaier I. (2021). Effect of Self-Regulatory Behaviour Change Techniques and Predictors of Physical Activity Maintenance in Cancer Survivors: A 12-Month Follow-up of the Phys-Can RCT. BMC Cancer.

[122] Strandberg E., Bean C., Vassbakk-Svindland K., Brooke H.L., Sjövall K., Börjeson S., Berntsen S., Nordin K., Demmelmaier I. (2022). Who Makes It All the Way? Participants vs. Decliners, and Completers vs. Drop-Outs, in a 6-Month Exercise Trial during Cancer Treatment. Results from the Phys-Can RCT. Support. Care Cancer.

[123] Brown J.C., Troxel A.B., Ky B., Damjanov N., Zemel B.S., Rickels M.R., Rhim A.D., Rustgi A.K., Courneya K.S., Schmitz K.H. (2016). A Randomized Phase II Dose-Response Exercise Trial among Colon Cancer Survivors: Purpose, Study Design, Methods, and Recruitment Results. Contemp. Clin. Trials.

[124] Brown J.C., Rhim A.D., Manning S.L., Brennan L., Mansour A.I., Rustgi A.K., Damjanov N., Troxel A.B., Rickels M.R., Ky B. (2018). Effects of Exercise on Circulating Tumor Cells among Patients with Resected Stage I-III Colon Cancer. PLoS ONE.

[125] Hubbard G., Adams R., Campbell A., Kidd L., Leslie S.J., Munro J., Watson A. (2016). Is Referral of Postsurgical Colorectal Cancer Survivors to Cardiac Rehabilitation Feasible and Acceptable? A Pragmatic Pilot Randomised Controlled Trial with Embedded Qualitative Study. BMJ Open.

[126] Bakker E.A., Hartman Y.A.W., Hopman M.T.E., Hopkins N.D., Graves L.E.F., Dunstan D.W., Healy G.N., Eijsvogels T.M.H., Thijssen D.H.J. (2020). Validity and Reliability of Subjective Methods to Assess Sedentary Behaviour in Adults: A Systematic Review and Meta-Analysis. Int. J. Behav. Nutr. Phys. Act..

[127] Hedges L.V., Tipton E., Johnson M.C. (2010). Robust Variance Estimation in Meta-Regression with Dependent Effect Size Estimates. Res. Synth. Methods.

[128] Samdal G.B., Eide G.E., Barth T., Williams G., Meland E. (2017). Effective Behaviour Change Techniques for Physical Activity and Healthy Eating in Overweight and Obese Adults; Systematic Review and Meta-Regression Analyses. Int. J. Behav. Nutr. Phys. Act..

[129] Cuijpers P., Griffin J.W., Furukawa T.A. (2021). The Lack of Statistical Power of Subgroup Analyses in Meta-Analyses: A Cautionary Note. Epidemiol. Psychiatr. Sci..

[130] Umer A., Kelley G.A., Cottrell L.E., Giacobbi P., Innes K.E., Lilly C.L. (2017). Childhood Obesity and Adult Cardiovascular Disease Risk Factors: A Systematic Review with Meta-Analysis. BMC Public Health.

[131] Turner B.E., Steinberg J.R., Weeks B.T., Rodriguez F., Cullen M.R. (2022). Race/Ethnicity Reporting and Representation in US Clinical Trials: A Cohort Study. Lancet Reg. Health.

[132] Chen M.S., Lara P.N., Dang J.H.T., Paterniti D.A., Kelly K. (2014). Twenty Years Post-NIH Revitalization Act: Enhancing Minority Participation in Clinical Trials (EMPaCT): Laying the Groundwork for Improving Minority Clinical Trial Accrual: Renewing the Case for Enhancing Minority Participation in Cancer Clinical Trials. Cancer.

[133] National Academies of Sciences and Medicine (2022). Improving Representation in Clinical Trials and Research: Building Research Equity for Women and Underrepresented Groups.

[134] Smirnoff M., Wilets I., Ragin D.F., Adams R., Holohan J., Rhodes R., Winkel G., Ricci E.M., Clesca C., Richardson L.D. (2018). A Paradigm for Understanding Trust and Mistrust in Medical Research: The Community VOICES Study. AJOB Empir. Bioeth..

[135] Quiñones A.R., Mitchell S.L., Jackson J.D., Aranda M.P., Dilworth-Anderson P., McCarthy E.P., Hinton L. (2020). Achieving Health Equity in Embedded Pragmatic Trials for People Living with Dementia and Their Family Caregivers. J. Am. Geriatr. Soc..

[136] Thetford K., Gillespie T.W., Kim Y.-I., Hansen B., Scarinci I.C. (2021). Willingness of Latinx and African Americans to Participate in Nontherapeutic Trials: It Depends on Who Runs the Research. Ethn. Dis..

[137] Alhajji M., Bass S.B., Nicholson A., Washington A., Maurer L., Geynisman D.M., Fleisher L. (2022). Comparing Perceptions and Decisional Conflict Towards Participation in Cancer Clinical Trials Among African American Patients Who Have and Have Not Participated. J. Cancer Educ..

[138] Palmer R.C., Schneider E.C. (2005). Social Disparities across the Continuum of Colorectal Cancer: A Systematic Review. Cancer Causes Control..

[139] Qwaider Y.Z., Sell N.M., Boudreau C., Stafford C.E., Ricciardi R., Cauley C.E., Bordeianou L.G., Berger D.L., Kunitake H., Goldstone R.N. (2021). Zip Code-Related Income Disparities in Patients with Colorectal Cancer. Am. Surg..

[140] Zahnd W.E., Davis M.M., Rotter J.S., Vanderpool R.C., Perry C.K., Shannon J., Ko L.K., Wheeler S.B., Odahowski C.L., Farris P.E. (2019). Rural-Urban Differences in Financial Burden among Cancer Survivors: An Analysis of a Nationally Representative Survey. Support. Care Cancer.

[141] Mama S.K., Bhuiyan N., Foo W., Segel J.E., Bluethmann S.M., Winkels R.M., Wiskemann J., Calo W.A., Lengerich E.J., Schmitz K.H. (2020). Rural-Urban Differences in Meeting Physical Activity Recommendations and Health Status in Cancer Survivors in Central Pennsylvania. Support. Care Cancer.

[142] Granger C.L., Holland A.E., Gordon I.R., Denehy L. (2015). Minimal Important Difference of the 6-Minute Walk Distance in Lung Cancer. Chron. Respir. Dis..

[143] Jones L.W., Hornsby W.E., Goetzinger A., Forbes L.M., Sherrard E.L., Quist M., Lane A.T., West M., Eves N.D., Gradison M. (2012). Prognostic Significance of Functional Capacity and Exercise Behavior in Patients with Metastatic Non-Small Cell Lung Cancer. Lung Cancer.

[144] Rücker G., Schumacher M. (2008). Simpson’s Paradox Visualized: The Example of the Rosiglitazone Meta-Analysis. BMC Med. Res. Methodol..

[145] Campbell K.L., Winters-Stone K.M., Wiskemann J., May A.M., Schwartz A.L., Courneya K.S., Zucker D.S., Matthews C.E., Ligibel J.A., Gerber L.H. (2019). Exercise Guidelines for Cancer Survivors: Consensus Statement from International Multidisciplinary Roundtable. Med. Sci. Sports Exerc..

