# Supplementary material for: A Systematic Review and Meta-Analysis of Physical Activity Interventions in Colorectal Cancer Survivors: An Evidence Evaluation Attempt Across Racial/Ethnic Groups
Source: Healthcare (Basel). 2025 Dec 5;13(24):3198. doi: 10.3390/healthcare13243198 (PMC12732477; doi:10.3390/healthcare13243198)
Supplement: Supplementary file 1 [file healthcare-13-03198-s001.zip › healthcare-3971801-supplementary.pdf]

healthcare

Systematic Review

A Systematic Review and Meta-Analysis of Physical Activity Interventions in Colorectal Cancer Survivors: An Evidence Evaluation Attempt Across Racial/Ethnic Groups

Yves Paul Vincent Mbous [1,*], Rowida Mohamed [2,*], George A. Kelley [3,†] and Kimberly Michelle Kelly [4,†]

1 School of Pharmacy, Department of Pharmaceutical Systems and Policy, West Virginia University,
Morgantown, WV 26506, USA
2 Pritzker School of Medicine, the University of Chicago, Chicago, IL 60637, USA
3 School of Public and Population Health and School of Kinesiology, Boise State University,
Boise, ID 83725, USA; georgekelley@boisestate.edu
4 Preventive Medicine, the University of Tennessee Health Science Center,
Memphis, TN 38163, USA; kkelly44@uthsc.edu
* Correspondence: ypm0001@mix.wvu.edu (Y.P.V.M.);
rowida.mohamed@bsd.uchicago.edu (R.M.)
† These authors contributed equally as co-senior authors to this work.

Table S1: PRISMA 2020 Checklist

Section and Topic	Item #	Checklist item	Location where item is reported
**TITLE**			
Title	1	Identify the report as a systematic review.	yes
**ABSTRACT**			
Abstract	2	See the PRISMA 2020 for Abstracts checklist.	
**INTRODUCTION**			
Rationale	3	Describe the rationale for the review in the context of existing knowledge.	Pg 3
Objectives	4	Provide an explicit statement of the objective(s) or question(s) the review addresses.	Pg
**METHODS**			
Eligibility criteria	5	Specify the inclusion and exclusion criteria for the review and how studies were grouped for the syntheses.	Pg 4
Information sources	6	Specify all databases, registers, websites, organisations, reference lists and other sources searched or consulted to identify studies. Specify the date when each source was last searched or consulted.	Pg 4
Search strategy	7	Present the full search strategies for all databases, registers and websites, including any filters and limits used.	Pg 4
Selection process	8	Specify the methods used to decide whether a study met the inclusion criteria of the review, including how many reviewers screened each record and each report retrieved, whether they worked independently, and if applicable, details of automation tools used in the process.	Pg 4 and 5
Data collection process	9	Specify the methods used to collect data from reports, including how many reviewers collected data from each report, whether they worked independently, any processes for obtaining or confirming data from study investigators, and if applicable, details of automation tools used in the process.	Pg 5
Data items	10a	List and define all outcomes for which data were sought. Specify whether all results that were compatible with each outcome domain in each study were sought (e.g. for all measures, time points, analyses), and if not, the methods used to decide which results to collect.	Pg 6
	10b	List and define all other variables for which data were sought (e.g. participant and intervention characteristics, funding sources). Describe any assumptions made about any missing or unclear information.	Pg 6
Study risk of bias assessment	11	Specify the methods used to assess risk of bias in the included studies, including details of the tool(s) used, how many reviewers assessed each study and whether they worked independently, and if applicable, details of automation tools used in the process.	Pg 7
Effect measures	12	Specify for each outcome the effect measure(s) (e.g. risk ratio, mean difference) used in the synthesis or presentation of results.	Pg 7
Synthesis methods	13a	Describe the processes used to decide which studies were eligible for each synthesis (e.g. tabulating the study intervention characteristics and comparing against the planned groups for each synthesis (item #5)).	Pg 7
	13b	Describe any methods required to prepare the data for presentation or synthesis, such as handling of missing summary statistics, or data conversions.	Pg 7
	13c	Describe any methods used to tabulate or visually display results of individual studies and syntheses.	Pg 7
	13d	Describe any methods used to synthesize results and provide a rationale for the choice(s). If meta-analysis was performed, describe the model(s), method(s) to identify the presence and extent of statistical heterogeneity, and software package(s) used.	Pg 7
	13e	Describe any methods used to explore possible causes of heterogeneity among study results (e.g. subgroup analysis, meta-regression).	Pg 8 and 9
	13f	Describe any sensitivity analyses conducted to assess robustness of the synthesized results.	Pg 8 and 9
Reporting bias	14	Describe any methods used to assess risk of bias due to missing results in a synthesis (arising from reporting biases).	Pg 6

Table S1: PRISMA 2020 Checklist

Section and Topic	Item #	Checklist item	Location where item is reported
assessment			
Certainty assessment	15	Describe any methods used to assess certainty (or confidence) in the body of evidence for an outcome.	Pg 8 and 9
**RESULTS**			
Study selection	16a	Describe the results of the search and selection process, from the number of records identified in the search to the number of studies included in the review, ideally using a flow diagram.	Pg 10
	16b	Cite studies that might appear to meet the inclusion criteria, but which were excluded, and explain why they were excluded.	Pg 10 - 12
Study characteristics	17	Cite each included study and present its characteristics.	Pg 10 - 12
Risk of bias in studies	18	Present assessments of risk of bias for each included study.	Pg 13
Results of individual studies	19	For all outcomes, present, for each study: (a) summary statistics for each group (where appropriate) and (b) an effect estimates and its precision (e.g. confidence/credible interval), ideally using structured tables or plots.	Pg 13-15
Results of syntheses	20a	For each synthesis, briefly summarise the characteristics and risk of bias among contributing studies.	Pg 13-15
	20b	Present results of all statistical syntheses conducted. If meta-analysis was done, present for each the summary estimate and its precision (e.g. confidence/credible interval) and measures of statistical heterogeneity. If comparing groups, describe the direction of the effect.	Pg 13-15
	20c	Present results of all investigations of possible causes of heterogeneity among study results.	Pg 15-16
	20d	Present results of all sensitivity analyses conducted to assess the robustness of the synthesized results.	Pg 15-16
Reporting biases	21	Present assessments of risk of bias due to missing results (arising from reporting biases) for each synthesis assessed.	Pg 13
Certainty of evidence	22	Present assessments of certainty (or confidence) in the body of evidence for each outcome assessed.	Pg. 13
**DISCUSSION**			
Discussion	23a	Provide a general interpretation of the results in the context of other evidence.	Pg 17-19
	23b	Discuss any limitations of the evidence included in the review.	Pg 23-24
	23c	Discuss any limitations of the review processes used.	Pg 23-24
	23d	Discuss implications of the results for practice, policy, and future research.	Pg 19-23
**OTHER INFORMATION**			
Registration and protocol	24a	Provide registration information for the review, including register name and registration number, or state that the review was not registered.	Pg 3
	24b	Indicate where the review protocol can be accessed, or state that a protocol was not prepared.	Pg 3
	24c	Describe and explain any amendments to information provided at registration or in the protocol.	Pg 3
Support	25	Describe sources of financial or non-financial support for the review, and the role of the funders or sponsors in the review.	Pg 25
Competing interests	26	Declare any competing interests of review authors.	Pg 25
Availability of data, code and other materials	27	Report which of the following are publicly available and where they can be found: template data collection forms; data extracted from included studies; data used for all analyses; analytic code; any other materials used in the review.	Pg 25

From: Page MJ, McKenzie JE, Bossuyt PM, Boutron I, Hoffmann TC, Mulrow CD, et al. The PRISMA 2020 statement: an updated guideline for reporting systematic reviews. BMJ 2021;372:n71. doi: 10.1136/bmj.n71

Table S2: Search Strategy	
**PubMed search strategy**	
**Concept**	**Search terms**
Population	*MeSH terms:* colorectal neoplasm, colonic neoplasm, rectal neoplasm<br>*Free text terms:* colorectal neoplasm*, colorectal tumor*, colorectal tumour*, colorectal malign*, colorectal carcinom*, colorectal lymphoma*, colonic neoplasm*, colon tumor*, colon tumour*, colon malign*, colon carcinom*, colon lymphoma*, rectum neoplasm*, rectal tumor*, rectal tumour*, rectal malign*, rectal carcinom*, rectal lymphoma*.
Intervention	*MeSH terms:* motor activity, exercise movement techniques, sport, exercise therapy, physical fitness, exercise<br>*Free text terms:* exercis*, physical activit*, fitness*, physical capacity*, physical health, motor activit*, physical training, endurance training, resistance training, physical therapy, physical therapy modalit*, motor activity, exercise therapy, muscle stretching, resistance training, strength training, weight training, aerobic exercise, physical exertion, running, swimming, walking.
Comparator	*MeSH terms:* placebo, control group<br>*Free text terms:* usual care, standard care, placebo, placebo control*, wait-list, wait-list control, passive group, passive control, no-contact control, control group
Outcomes	*MeSH terms:* health behavior, health behaviour, motivation, health education, health promotion.<br>*Free text terms:* theory-based health behavior, theory-based health behaviour, behaviour change technique*, behaviour* therapy, behavior* therapy, behaviour* change*, behavior* change*, behavior change strateg*, behaviour change strateg*, behavior change technique*, behavior* modification*, behaviour* modification*, behaviour*, behavior*, theory-based, behaviour* change*, behaviour uptak*, behavior uptak*, MVPA, MET-hours, MET
Study design/setting	*MeSH terms*: clinical trial, randomized controlled trial<br>*Free text terms:* randomized clinical trial, random* clinical trial, RCT
**Cochrane (CENTRAL) search strategy**	
**Concept**	**Search terms**
Population	*MeSH descriptors:* colorectal neoplasm, colonic neoplasm, rectal neoplasm<br>*Title/abstract/keywords:* colorectal neoplasm*, colorectal tumor*, colorectal tumour*, colorectal malign*, colorectal carcinom*, colorectal lymphoma*, colonic neoplasm*, colon tumor*, colon tumour*, colon malign*, colon carcinom*, colon lymphoma*, rectum neoplasm*, rectal tumor*, rectal tumour*, rectal malign*, rectal carcinom*, rectal lymphoma*.
Intervention	*MeSH descriptors:* motor activity, exercise movement techniques, sport, exercise therapy, physical fitness, exercise<br>*Title/abstract/keywords:* exercis*, physical activit*, fitness*, physical capacity*, physical health, motor activit*, physical training, endurance training, resistance training, physical therapy, physical therapy modalit*, motor activity, exercise therapy, muscle stretching, resistance training, strength training, weight training, aerobic exercise, physical exertion, running, swimming, walking.
Comparator	*MeSH descriptors:* placebo, control group<br>*Title/abstract/keywords:* usual care, standard care, placebo, placebo control*, wait-list, wait-list control, passive group, passive control, no-contact control, control group
Outcomes	*MeSH descriptors:* health behavior, motivation, health education, health promotion<br>*Title/abstract/keywords:* theory-based health behavior, theory-based health behaviour, behaviour change technique*, behaviour* therapy, behavior* therapy, behaviour*change*, behavior* change*, behavior change strateg*, behaviour change strateg*, behavior change technique*, behavior* modification*, behaviour* modification*, behaviour*, behavior*,

	theory-based, behaviour* change*, behaviour uptak*,behavior uptak*, MVPA, MET-hours, MET
Study design/setting	*MeSH descriptors*: clinical trial, randomized controlled trial<br>*Title/abstract/keywords:* randomized clinical trial, random* clinical trial, RCT
**CINHAL and PSYINFO search strategy**	
**Concept**	**Search terms**
Population	colorectal neoplasm, colonic neoplasm, rectal neoplasm<br>colorectal neoplasm*, colorectal tumor*, colorectal tumour*, colorectal malign*, colorectal carcinom*, colorectal lymphoma*, colonic neoplasm*, colon tumor*, colon tumour*, colon malign*, colon carcinom*, colon lymphoma*, rectum neoplasm*, rectal tumor*, rectal tumour*, rectal malign*, rectal carcinom*, rectal lymphoma*.
Intervention	motor activity, exercise movement techniques, sport, exercise therapy, physical fitness, exercise<br>exercis*, physical activit*, fitness*, physical capacity*, physical health, motor activit*, physical training, endurance training, resistance training, physical therapy, physical therapy modalit*, motor activity, exercise therapy, muscle stretching, resistance training, strength training, weight training, aerobic exercise, physical exertion, running, swimming, walking.
Comparator	placebo, control group<br>*Free text terms:* usual care, standard care, placebo, placebo control*, wait-list, wait-list control, passive group, passive control, no-contact control, control group
Outcomes	health behavior, health behaviour, motivation, health education, health promotion.<br>theory-based health behavior, theory-based health behaviour, behaviour change technique*, behaviour* therapy, behavior* therapy, behaviour* change*, behavior* change*, behavior change strateg*, behaviour change strateg*, behavior change technique*, behavior* modification*, behaviour* modification*, behaviour*, behavior*, theory-based, behaviour* change*, behaviour uptak*, behavior uptak*, MVPA, MET-hours, MET
Study design/setting	clinical trial, randomized controlled trial<br>randomized clinical trial, random* clinical trial, RCT
**Web of Science search strategy**	
**Concept**	**Search terms**
Population (TS or TI or AB)	(colorectal neoplasm, colonic neoplasm, rectal neoplasm<br>colorectal neoplasm*, colorectal tumor*, colorectal tumour*, colorectal malign*, colorectal carcinom*, colorectal lymphoma*, colonic neoplasm*, colon tumor*, colon tumour*, colon malign*, colon carcinom*, colon lymphoma*, rectum neoplasm*, rectal tumor*, rectal tumour*, rectal malign*, rectal carcinom*, rectal lymphoma*).
Intervention(TS or TI or AB)	(motor activity, exercise movement techniques, sport, exercise therapy, physical fitness, exercise<br>exercis*, physical activit*, fitness*, physical capacity*, physical health, motor activit*, physical training, endurance training, resistance training, physical therapy, physical therapy modalit*, motor activity, exercise therapy, muscle stretching, resistance training, strength training, weight training, aerobic exercise, physical exertion, running, swimming, walking).
Comparator (TS or TI or AB)	(placebo, control group<br>*Free text terms:* usual care, standard care, placebo, placebo control*, wait-list, wait-list control, passive group, passive control, no-contact control, control group)

Outcomes (TS or TI or AB)	(health behavior, health behaviour, motivation, health education, health promotion.<br>theory-based health behavior, theory-based health behaviour,<br>behaviour change technique*, behaviour* therapy, behavior* therapy, behaviour* change*, behavior* change*, behavior change strateg*, behaviour change strateg*, behavior change technique*, behavior* modification*, behaviour* modification*, behaviour*, behavior*, theory-based, behaviour* change*, behaviour uptak*, behavior uptak*, MVPA, MET-hours, MET)
Study design (TS or TI or AB)	(clinical trial, randomized controlled trial<br>randomized clinical trial, random* clinical trial, RCT)

*MVPA: Moderate to vigorous physical activity; *MET-hours: Metabolic equivalent hours

Table S3: List of excluded studies with reasons

1. Effects of dyadic coping intervention on psychological distress and intimate relationships among bowel cancer patients and their spouses. Chinese Nursing Research Aug 2019 33(15)2568--2572 Intervention
2. Effect of preventive messages tailored to family history on health behaviors: the Family Healthware Impact Trial. Annals of Family Medicine Jan 2011 9(1)3--11 Intervention
3. Surgical approach to hysterectomy for benign gynaecological disease Cochrane Database of Systematic Reviews 2015 (8) Not applicable
4. Adalimumab for induction of remission in Crohn's disease Cochrane Database of Systematic Reviews 2019 (11) Not applicable
5. Laparoscopic versus open resection for sigmoid diverticulitis Cochrane Database of Systematic Reviews 2017 (11) Population
6. No effect of exercise on colon mucosal prostaglandin concentrations: a 12-month randomized controlled trial Cancer epidemiology, biomarkers {\&} prevention 2007 16(11)2351--2356 Population
7. Electronic patient self-Reporting of Adverse-events: Patient Information and aDvice (eRAPID): a randomised controlled trial in systemic cancer treatment. BMC Cancer May 2017 17()1--16 Intervention
8. Rethinking exercise identity: a qualitative study of physically inactive cancer patients' transforming process while undergoing chemotherapy. BMJ open Aug 2017 7(8)16689 Outcome
9. Peer support for the maintenance of physical activity and health in cancer survivors: the PEER trial - a study protocol of a randomised controlled trial Bmc Cancer 2019 19() Not applicable
10. Laparoscopic entry techniques Cochrane Database of Systematic Reviews 2019 (1) Not applicable
11. Supportive care for patients with gastrointestinal cancer Cochrane Database of Systematic Reviews 2004 (3) Not applicable
12. Interventions for preventing neuropathy caused by cisplatin and related compounds Cochrane Database of Systematic Reviews 2014 (3) Not applicable
13. Nutritional Adequacy and Diet Quality in Colorectal Cancer Patients Postsurgery: A Pilot Study Nutrition and Cancer-an International Journal 2016 68(4)577--588 Outcome
14. Bipolar versus monopolar transurethral resection of the prostate for lower urinary tract symptoms secondary to benign prostatic obstruction Cochrane Database of Systematic Reviews 2019 (12) Not applicable
15. Surgical cytoreduction for recurrent epithelial ovarian cancer Cochrane Database of Systematic Reviews 2013 (2) Not applicable
16. Diet and risk of inflammatory bowel disease Digestive and Liver Disease 2012 44(3)185--194 Not applicable
17. Health benefits of dietary fiber Nutrition Reviews 2009 67(4)188--205 Intervention
18. The impact of a bodyweight and physical activity intervention (BeWEL) initiated through a national colorectal cancer screening programme: randomised controlled trial. BMJ (Clinical research ed.) 2014 348()1823
19. Interest and informational preferences regarding genomic testing for modest increases in colorectal cancer risk Public Health Genomics 2014 (1)48--60 Population
20. Awareness of Lifestyle and Colorectal Cancer Risk: Findings from the BeWEL Study. BioMed research international 2015 2015()871613 Population
21. Conservative management for postprostatectomy urinary incontinence Cochrane Database of Systematic Reviews 2015 (1) Not applicable
22. Feasibility study to assess the impact of a lifestyle intervention ('LivingWELL') in people having an assessment of their family history of colorectal or breast cancer. BMJ open 2018 8(2)e019410 Outcome
23. Interventions for chronic pruritus of unknown origin Cochrane Database of Systematic Reviews 2020 (1) Not applicable
24. Sociodemographic profiles regarding bitter food consumption. Cross-sectional evidence from a general French population Appetite 2013 67()53--60 Intervention
25. Mortality results from a randomized prostate-cancer screening trial. The New England journal of medicine Mar 2009 360(13)1310--1319 Population
26. The immune system and mental health 2018 ()1--700 Not applicable
27. Evaluation of the EORTC QLQ-C30 questionnaire: A comparison with SF-36 Health Survey in a cohort of Italian long-survival cancer patients Annals of Oncology 1998 9(5)549--557 Population
28. A randomized controlled trial of a multilevel intervention to increase colorectal cancer screening among Latino immigrants in a primary care facility. Journal of general internal medicine Jun 2010 25(6)564--567 Population
29. ESPEN guidelines on nutrition in cancer patients Clinical Nutrition 2017 () Not applicable
30. Transperitoneal versus retroperitoneal laparoscopic adrenalectomy for adrenal tumours in adults Cochrane Database of Systematic Reviews 2018 (12) Not applicable

31. A Comprehensive Lifestyle Randomized Clinical Trial: Design and Initial Patient Experience Integrative Cancer Therapies 2017 16(1)3--20 Population
32. Early rehabilitation of cancer patients - a randomized controlled intervention study. BMC cancer 2013 13()9 Intervention
33. Coping with colorectal cancer: a qualitative exploration with patients and their family members. Family practice Oct 2014 31(5)598--606 Intervention
34. Design of a multicentre randomised trial to evaluate flexible sigmoidoscopy in colorectal cancer screening. Journal of medical screening Sep 2001 8(3)137--144 Population
35. The effectiveness of adherence intervention in a colon cancer prevention field trial. Preventive medicine Sep 1992 21(5)637--653 Population
36. Interventions to improve continuity of care in the followâ€ up of patients with cancer Cochrane Database of Systematic Reviews 2012 (7) Not applicable
37. Efficacy of communication skills training on colorectal cancer screening by GPs: a cluster randomised controlled trial. European Journal of Cancer Care Jan 2016 25(1)18--26 Intervention
38. Healthy eating interventions in adults living with and beyond colorectal cancer: a systematic review Journal of Human Nutrition and Dietetics 2019 32(4)501--511 Not applicable
39. Postoperative interventions for preventing bladder dysfunction after radical hysterectomy in women with earlyâ€ stage cervical cancer Cochrane Database of Systematic Reviews 2017 (11) Not applicable
40. Evaluation of the Efficacy of the Three-Component Health Care Management Program HEWCOT in Colorectal Cancer Patients Receiving Chemotherapy. Journal of cancer education : the official journal of the American Association for Cancer Education Apr 2020 35(2)274--283 Outcome
41. A randomized pilot study with daily walking during adjuvant chemotherapy for patients with breast and colorectal cancer. Acta oncologica (Stockholm, Sweden) 2014 53(4)510--520 Outcome
42. The effectiveness and costâ€ effectiveness of hospitalâ€ based specialist palliative care for adults with advanced illness and their caregivers Cochrane Database of Systematic Reviews 2020 (9) Not applicable
43. Increasing fruit and vegetable intake among adults attending colorectal cancer screening: the efficacy of a brief tailored intervention. Cancer epidemiology, biomarkers {\&} prevention : a publication of the American Association for Cancer Research, cosponsored by the American Society of Preventive Oncology Feb 2002 11(2)203--206 Population
44. Design of a randomized controlled trial to assess the comparative effectiveness of a multifaceted intervention to improve adherence to colorectal cancer screening among patients cared for in a community health center. BMC health services research Apr 2013 13()153 Population
45. Antiâ€ cytokine targeted therapies for ANCAâ€ associated vasculitis Cochrane Database of Systematic Reviews 2020 (9) Not applicable
46. Statins for preventing colorectal adenoma and carcinoma Cochrane Database of Systematic Reviews 2014 (5) Not applicable
47. Socioeconomic and racial/ethnic differences in the discussion of cancer screening: between-" versus "within-" physician differences." Health services research Jun 2007 42(3)950--970 Outcome
48. Hydromorphone for cancer pain Cochrane Database of Systematic Reviews 2016 (10) Not applicable
49. Interventions to encourage uptake of cancer screening for people with severe mental illness Cochrane Database of Systematic Reviews 2016 (9) Not applicable
50. International union of basic and clinical pharmacology. Cx. Classification of receptors for 5-hydroxytryptamine; pharmacology and function Pharmacological Reviews 2021 73(1)310--520 Not applicable
51. Client-directed interventions to increase community demand for breast, cervical, and colorectal cancer screening a systematic review. American journal of preventive medicine Jul 2008 35(1)S34--55 Study design
52. A randomized pilot trial of a telephone-based couples intervention for physical intimacy and sexual concerns in colorectal cancer. Psycho-oncology 2014 () Intervention
53. A Randomized Trial to Compare Alternative Educational Interventions to Increase Colorectal Cancer Screening in a Hard-to-Reach Urban Minority Population with Health Insurance. Journal of community health Oct 2015 40(5)975--983 Population
54. Invitation to screening colonoscopy in the population at familial risk for colorectal cancer Deutsches Arzteblatt international 2018 () Population
55. ENDCAT: endometrial cancer telephone follow-up trial Psycho-oncology 2013 22()19-- Outcome
56. Pharmacological interventions for pain in children and adolescents with lifeâ€ limiting conditions Cochrane Database of Systematic Reviews 2015 (3) Study design
57. Study protocol for a randomised controlled trial of brief, habit-based, lifestyle advice for cancer survivors: exploring behavioural outcomes for the Advancing Survivorship Cancer Outcomes Trial (ASCOT). BMJ open Nov 2016 6(11)e011646 Outcome
58. Pharmacological treatment of depression in people with a primary brain tumour Cochrane Database of Systematic Reviews 2020 (7) Study design

59. The impact of anxiety and depression in the quality of life and psychological well-being of Greek hematological cancer patients on chemotherapy Psychology Health {\&} Medicine 2020 25(2)201--213 Population
60. Are community-based health worker interventions an effective approach for early diagnosis of cancer? A systematic review and meta-analysis. Psycho-oncology Apr 2018 27(4)1089--1099 Study design
61. Is colorectal cancer an avoidable disease nowadays? Best Practice and Research: Clinical Gastroenterology 2004 () Study design
62. Traditional corticosteroids for induction of remission in Crohn's disease Cochrane Database of Systematic Reviews 2008 (2) Study design
63. Educational interventions for the management of cancerâ€ related fatigue in adults Cochrane Database of Systematic Reviews 2016 (11) Study design
64. Hyperbaric oxygenation for tumour sensitisation to radiotherapy Cochrane Database of Systematic Reviews 2018 (4) Study design
65. Neural circuitry mediating inflammation-induced central pain amplification in human experimental endotoxemia. Brain, behavior, and immunity Aug 2015 48()222--231 Outcome
66. Patterns of circadian activity rhythms and their relationships with fatigue and anxiety/depression in women treated with breast cancer adjuvant chemotherapy Supportive Care in Cancer 2010 18(1)105--114 Outcome
67. Lifestyle changes at middle age and mortality: A population-based prospective cohort study Journal of Epidemiology and Community Health 2016 71(1)59--66 Outcome
68. Patient perspectives on research recruitment through cancer registries. Cancer causes {\&} control : CCC Dec 2005 16(10)1171--1175 Outcome
69. Palliative chemotherapy for advanced or metastatic colorectal cancer Cochrane Database of Systematic Reviews 2000 (1) Study design
70. Laparoscopic versus open gastrectomy for gastric cancer Cochrane Database of Systematic Reviews 2016 (3) Study design
71. Evidence-based complementary therapeutic measures in prostate cancer Deutsche zeitschrift fur onkologie 2002 34(3)86--90 Population
72. Control Group Design, Contamination and Drop-Out in Exercise Oncology Trials: A Systematic Review Plos One 2015 10(3) Study design
73. Antioxidant supplements for prevention of mortality in healthy participants and patients with various diseases Cochrane Database of Systematic Reviews 2012 (3) Study design
74. Is physical activity or physical fitness more important in defining health benefits?. Medicine and science in sports and exercise 2001 33(6)S379--20 Study design
75. Correlates of poor adherence to a healthy lifestyle among a diverse group of colorectal cancer survivors Cancer Causes {\&} Control 2019 30(12)1327--1339 Outcome
76. A trial of 3 interventions to promote colorectal cancer screening in African Americans. Cancer Feb 2010 116(4)922--929 Population
77. Endogenous opiates and behavior: 2013 Peptides 2014 62()67--136 Population
78. A randomized double-blind placebo-controlled trial to evaluate the value of a single bolus intravenous alfentanil in CT colonography. BMC gastroenterology Nov 2011 11()128 Population
79. Erythropoietin or Darbepoetin for patients with cancer â€ metaâ€ analysis based on individual patient data Cochrane Database of Systematic Reviews 2009 (3) Study design
80. Principles and practice of phytotherapy: Modern herbal medicine 2012 ()1--1051 Study design
81. Low Back and Neck Pain: Comprehensive Diagnosis and Management: Third Edition 2004 ()1--921 Study design
82. Interventions for promoting habitual exercise in people living with and beyond cancer. The Cochrane database of systematic reviews 2013 (9)CD010192 Study design
83. Interventions to improve exercise behaviour in sedentary people living with and beyond cancer: a systematic review. British journal of cancer Feb 2014 110(4)831--841 Study design
84. Testing a culturally appropriate, theory-based intervention to improve colorectal cancer screening among Native Hawaiians. Preventive medicine Jun 2005 40(6)619--627 Population
85. Reducing cancer screening disparities in medicare beneficiaries through cancer patient navigation. Journal of the American Geriatrics Society Feb 2015 63(2)365--370 Outcome
86. Comparing the effect of a decision aid plus patient navigation with usual care on colorectal cancer screening completion in vulnerable populations: study protocol for a randomized controlled trial. Trials Jul 2014 15()275 Population
87. Carbon dioxide insufflation reduces discomfort due to flexible sigmoidoscopy in colorectal cancer screening. Scandinavian journal of gastroenterology Sep 2002 37(9)1103--1107 Population
88. Population-Based Colonoscopy Screening for Colorectal Cancer: A Randomized Clinical Trial. JAMA internal medicine Jul 2016 176(7)894--902 Population

89.	Assessing the Psychological Impact of Daily Bowel Preparation on Prostate Patients Who Receive Radiation Therapy. Journal of medical imaging and radiation sciences Mar 2018 49(1)70--75 Population
90.	Screening programmes for the early detection and prevention of oral cancer Cochrane Database of Systematic Reviews 2013 (11) Population
91.	The impact of illustrations on public understanding of the aim of cancer screening. Patient education and counseling Nov 2006 63(3)328--335 Intervention
92.	The efficacy of exercise in reducing depressive symptoms among cancer survivors: a meta-analysis. PloS one 2012 7(1)e30955 Study design
93.	Strategies to improve retention in randomised trials Cochrane Database of Systematic Reviews 2013 (12) Study design
94.	FOLLOW-UP AFTER COLORECTAL-CANCER - CURRENT PRACTICE IN THE NETHERLANDS European Journal of Surgery 1995 161(11)827--831 Outcome
95.	Improving Outcomes in Oncological Colorectal Surgery by Prehabilitation American Journal of Physical Medicine and Rehabilitation 2019 98(3)231--238 Study design
96.	Clinical aromatherapy: Essential oils in healthcare 2014 ()1--432 Study design
97.	Muscle contractile properties of cancer patients receiving chemotherapy: Assessment of feasibility and exercise effects Scandinavian Journal of Medicine {\&} Science in Sports 2020 30(10)1918--1929 Outcome
98.	An unblinded randomised controlled trial of preoperative oral supplements in colorectal cancer patients. Journal of Human Nutrition {\&} Dietetics Oct 2011 24(5)441--448 Outcome
99.	Preâ€ operative Nutrition Support in Patients Undergoing Gastrointestinal Surgery. Cochrane Database of Systematic Reviews 2012 (11) Study design
100.	Perioperative nutrition for the treatment of bladder cancer by radical cystectomy Cochrane Database of Systematic Reviews 2019 (5) Study design
101.	Dietary interventions for adult cancer survivors Cochrane Database of Systematic Reviews 2019 (11) Study design
102.	Herbal principles in cosmetics: Properties and mechanisms of action 2010 ()1--381
103.	Conquer fear: protocol of a randomised controlled trial of a psychological intervention to reduce fear of cancer recurrence. BMC cancer Apr 2013 13()201 Intervention
104.	Evaluation of the efficacy and usability of NCI's Facing Forward booklet in the cancer community setting Journal of cancer survivorship 2013 7(1)63--73 Outcome
105.	Anaesthetic techniques for risk of malignant tumour recurrence Cochrane Database of Systematic Reviews 2014 (11) Study design
106.	Interventions for sexual dysfunction following treatments for cancer in women Cochrane Database of Systematic Reviews 2016 (2) Study design
107.	Cancer and elders of color: Opportunities for reducing health disparities: Evidence review and recommendations for research and policy 2019 ()1--259 Study design
108.	Feasibility of an expressive-disclosure group intervention for post-treatment colorectal cancer patients: results of the Healthy Expressions study. Cancer Nov 2011 117(21)4993--5002 Outcome
109.	Improving colorectal cancer screening in Asian Americans: Results of a randomized intervention study. Cancer Jun 2014 120(11)1702--1712 Intervention
110.	Low-fat dietary pattern and change in body-composition traits in the Women's Health Initiative Dietary Modification Trial. The American journal of clinical nutrition Mar 2011 93(3)516--524 Intervention
111.	SAFETY PROFILE OF ELUXADOLINE IN PATIENTS WITH IRRITABLE BOWEL SYNDROME WITH DIARRHEA REPORTING INADEQUATE SYMPTOM CONTROL WITH PRIOR LOPERAMIDE USE: A PHASE 4 STUDY (RELIEF) Gastroenterology 2019 156(6)S91--S92 Population
112.	Outreach and Inreach Strategies for Colorectal Cancer Screening Among Latinos at a Federally Qualified Health Center: A Randomized Controlled Trial, 2015-2018. American journal of public health Apr 2020 110(4)587--594 Population
113.	Bowel health to better health: a minimal contact lifestyle intervention for people at increased risk of colorectal cancer. The British journal of nutrition 2009 102(11)1541--1546 Population
114.	Preoperative exercise training for patients with nonâ€ small cell lung cancer Cochrane Database of Systematic Reviews 2017 (6) Population
115.	Oxaliplatin-associated neuropathy: A review Annals of Pharmacotherapy 2005 39(1)128--135 Study design
116.	Colorectal Cancer Screening and Prevention in Women Digestive Diseases and Sciences 2015 60(3)698--710 Study design
117.	Randomized controlled trial of intraoperative goal-directed fluid therapy in aerobically fit and unfit patients having major colorectal surgery. British journal of anaesthesia 2012 108(1)53--62 Outcome
118.	Implementing an intervention to promote colon cancer screening through e-mail over the Internet: lessons learned from a pilot study. Medical care Sep 2008 46(9)S117--22 Outcome

119.	Lipid biomarkers and long-term risk of cancer in the Women's Health Study American Journal of Clinical Nutrition 2016 103(6)1397--1407 Outcome
120.	Evaluation of a home-based colorectal cancer screening intervention in a rural state. The Journal of rural health : official journal of the American Rural Health Association and the National Rural Health Care Association 2014 30(3)322--332 Population
121.	Early versus delayed oral fluids and food for reducing complications after major abdominal gynaecologic surgery Cochrane Database of Systematic Reviews 2014 (12) Study design
122.	Preoperative intensive, community-based vs. traditional stoma education: a randomized, controlled trial. Diseases of the colon and rectum Mar 2005 48(3)504--509 Outcome
123.	Randomised controlled trial on the effectiveness of home-based walking exercise on anxiety, depression and cancer-related symptoms in patients with lung cancer British Journal of Cancer 2015 112(3)438--445 Population
124.	Transcranial magnetic stimulation for the treatment of epilepsy Cochrane Database of Systematic Reviews 2016 (8) Study design
125.	Comparative Evaluation of Participation and Diagnostic Yield of Colonoscopy vs Fecal Immunochemical Test vs Risk-Adapted Screening in Colorectal Cancer Screening: Interim Analysis of a Multicenter Randomized Controlled Trial (TARGET-C). The American journal of gastroenterology Aug 2020 115(8)1264--1274 Population
126.	Early versus delayed appendicectomy for appendiceal phlegmon or abscess Cochrane Database of Systematic Reviews 2017 (6) Study design
127.	The effect of progressive muscle relaxation training on anxiety and quality of life after stoma surgery in colorectal cancer patients. Psycho-oncology 2003 12(3)254--266 Outcome
128.	Irritable bowel syndrome: a clinical review. JAMA 2015 313(9)949--958 Study design
129.	Oral versus intravenous fluoropyrimidines for colorectal cancer Cochrane Database of Systematic Reviews 2017 (7) Study design
130.	Effects of a health education and telephone counseling program on patients with a positive fecal occult blood test result for colorectal cancer screening: A randomized controlled trial. Psycho-oncology Oct 2017 26(10)1498--1504 Intervention
131.	Comparisons of exercise dose and symptom severity between exercisers and nonexercisers in women during and after cancer treatment. Journal of pain and symptom management 2012 43(5)842--854 Outcome
132.	Mass screening for colorectal cancer: compliance in Almopea Region. Techniques in coloproctology Nov 2004 8 Suppl 1()s193--5 Population
133.	A randomized controlled trial using patient navigation to increase colonoscopy screening among low-income minorities. Journal of the National Medical Association Mar 2008 100(3)278--284 Population
134.	Health Literacy among Medically Underserved: The Role of Demographic Factors, Social Influence, and Religious Beliefs. Journal of health communication Nov 2017 22(11)923--931 Population
135.	Examining the Durability of Colorectal Cancer Screening Awareness and Health Beliefs Among Medically Underserved Patients: Baseline to 12Â months Post-Intervention. Journal of cancer education : the official journal of the American Association for Cancer Education Apr 2019 34(2)297--303 Population
136.	Survival differences with immediate versus delayed chemotherapy for asymptomatic incurable metastatic colorectal cancer Cochrane Database of Systematic Reviews 2018 (11) Study design
137.	Evaluation of followâ€ up strategies for patients with epithelial ovarian cancer following completion of primary treatment Cochrane Database of Systematic Reviews 2014 (9) Study design
138.	Age-related vulnerabilities of older adults with colon adenomas: evidence from Project Prevent Cancer 2004 100(5)1085--1094 Outcome
139.	Does access to a colorectal cancer screening website and/or a nurse-managed telephone help line provided to patients by their family physician increase fecal occult blood test uptake?: A pragmatic cluster randomized controlled trial study protocol. BMC cancer May 2012 12()182 Population
140.	Urinary diversion and bladder reconstruction/replacement using intestinal segments for intractable incontinence or following cystectomy Cochrane Database of Systematic Reviews 2012 (2) Study design
141.	Community-Based, Preclinical Patient Navigation for Colorectal Cancer Screening Among Older Black Men Recruited From Barbershops: The MISTER B Trial. American Journal of Public Health Sep 2017 107(9)1433--1440 Intervention
142.	Patient knowledge about hormone replacement therapy: implications for treatment. Menopause (New York, N.Y.) 2000 7(4)266--272 Outcome
143.	Opioids for chronic nonâ€ cancer pain in children and adolescents Cochrane Database of Systematic Reviews 2017 (7) Study design
144.	Antiepileptic drugs for chronic nonâ€ cancer pain in children and adolescents Cochrane Database of Systematic Reviews 2017 (8) Study design

145. Effectiveness of a clinic-based colorectal cancer screening promotion program for underserved Hispanics. Cancer Apr 2011 117(8)1745--1754 Population
146. Oral isotretinoin for acne Cochrane Database of Systematic Reviews 2018 (11) Study design
147. Use of consumer wearable devices to promote physical activity among breast, prostate, and colorectal cancer survivors: a review of health intervention studies Journal of Cancer Survivorship 2020 14(3)386--392 Study design
148. Personalised care planning for adults with chronic or longâ€ term health conditions Cochrane Database of Systematic Reviews 2015 (3) Study design
149. Predictors of adherence and contamination in a randomized trial of exercise in colorectal cancer survivors. Psycho-oncology Dec 2004 13(12)857--866 Outcome
150. A longitudinal study of exercise barriers in colorectal cancer survivors participating in a randomized controlled trial. Annals of behavioral medicine : a publication of the Society of Behavioral Medicine Outcome
151. Physical activity in cancer survivors: a field in motion. Psycho-Oncology Apr 2009 18(4)337--342 Study design
152. Colorectal cancer risk assessment and screening recommendation: a community survey of healthcare providers' practice from a patient perspective Bmc Family Practice 2012 13() Outcome
153. Applying the Plan-Do-Study-Act (PDSA) approach to a large pragmatic study involving safety net clinics. BMC health services research Jun 2017 17(1)411 Study design
154. Exercise Effects on Depressive Symptoms in Cancer Survivors: A Systematic Review and Meta-analysis Cancer Epidemiology Biomarkers {\&} Prevention 2012 21(1)3--19 Study design
155. Study protocol for BeWEL: the impact of a BodyWEight and physicaL activity intervention on adults at risk of developing colorectal adenomas. BMC public health 2011 11()184 Population
156. A systematic review and meta-analysis of exercise interventions for colorectal cancer patients. European journal of cancer care 2014 23(1)3--14 Study design
157. Exercise for the management of cancerâ€ related fatigue in adults Cochrane Database of Systematic Reviews 2012 (11) Study design
158. Study of Durvalumab or Placebo given along with Standard Chemotherapy in Patients with Non-small Cell Lung Cancer (Stage II-III) who\^a??s tumor has been removed through surgery http://www.who.int/trialsearch/Trial2.aspx?TrialID=CTRI/2020/09/027739 2020 Population
159. Results of a lay health education intervention to increase colorectal cancer screening among Filipino Americans: A cluster randomized controlled trial. Cancer 2018 124()1535--1542 Population
160. Nordic Pole Walking for Individuals with Cancer: a Feasibility Randomized Controlled Trial Assessing Physical Function and Health-Related Quality of Life Rehabilitation oncology 2020 38(2)81--91 Outcome
161. Patients whose physicians recommend colonoscopy and those who follow through Journal of primary care {\&} community health 2013 4(2)83--94 Population
162. Effects of the Dietary Flavonoid Quercetin Upon Performance and Health. Current Sports Medicine Reports (American College of Sports Medicine) Jul 2009 8(4)206--213 Study design
163. A randomized controlled trial of a multicomponent, targeted, low-literacy educational intervention compared with a nontargeted intervention to boost colorectal cancer screening with fecal immunochemical testing in community clinics. Cancer Apr 2017 123(8)1390--1400 Population
164. Interventions for preventing and ameliorating cognitive deficits in adults treated with cranial irradiation Cochrane Database of Systematic Reviews 2014 (12) Study design
165. Interventions to enhance returnâ€ toâ€ work for cancer patients Cochrane Database of Systematic Reviews 2015 (9) Study design
166. Informing relatives about their hereditary or familial cancer risk: study protocol for a randomized controlled trial. Trials Mar 2014 15()86 Study design
167. Improving recognition and referral of patients with an increased familial risk of colorectal cancer: results from a randomized controlled trial. Colorectal disease : the official journal of the Association of Coloproctology of Great Britain and Ireland Jun 2015 17(6)499--510 Intervention
168. Pilot Randomized Controlled Trial of a Home Vegetable Gardening Intervention among Older Cancer Survivors Shows Feasibility, Satisfaction, and Promise in Improving Vegetable and Fruit Consumption, Reassurance of Worth, and the Trajectory of Central Adipos Journal of the Academy of Nutrition and Dietetics Outcome
169. Electronic Health Program to Empower Patients in Returning to Normal Activities After Colorectal Surgical Procedures: Mixed-Methods Process Evaluation Alongside a Randomized Controlled Trial. Journal of medical Internet research Jan 2019 21(1)e10674 Intervention
170. Tailored telephone counselling to increase participation of underusers in a population-based colorectal cancer-screening programme with faecal occult blood test: A randomized controlled trial. Revue d'epidemiologie et de sante publique Feb 2017 65(1)17--28 Population
171. Short- and long-term effects of tailored information versus general information on determinants and intentions related to early detection of cancer. Preventive medicine Jun 2004 38(6)694--703 Outcome

172. Patient burden of colonoscopy after positive fecal immunochemical testing for colorectal cancer screening. Endoscopy 2013 45(5)342--349 Population
173. Nonâ€ surgical interventions for late radiation cystitis in patients who have received radical radiotherapy to the pelvis Cochrane Database of Systematic Reviews 2002 (3) Study design
174. Interventions for the physical aspects of sexual dysfunction in women following pelvic radiotherapy Cochrane Database of Systematic Reviews 2003 (1) Study design
175. Flaxseed lignans as important dietary polyphenols for cancer prevention and treatment: Chemistry, pharmacokinetics, and molecular targets Pharmaceuticals 2019 12(2) Outcome
176. Meat: The balance between nutrition and health. A review Meat Science 2016 120()145--156 Study design
177. The effects of screening on health behaviour: A summary of the results of randomized controlled trials Journal of Public Health 2011 () Population
178. Eicosapentaenoic acid (EPA, an omegaâ€ 3 fatty acid from fish oils) for the treatment of cancer cachexia Cochrane Database of Systematic Reviews 2007 (1) Outcome
179. Adenoma detection with cap-assisted colonoscopy versus regular colonoscopy: a randomised controlled trial. Gut Oct 2012 61(10)1426--1434 Population
180. The impact of physical activity on fatigue and quality of life in lung cancer patients: a randomised controlled trial protocol. BMC cancer Dec 2012 12()572 Population
181. Telephone outreach to increase colon cancer screening in medicaid managed care organizations: a randomized controlled trial. Annals of family medicine 2013 11(4)335--343 Population
182. Effectiveness of a primary care practice intervention for increasing colorectal cancer screening in Appalachian Kentucky. Preventive medicine Jan 2014 58()70--74 Population
183. Vaccines for measles, mumps, rubella, and varicella in children Cochrane Database of Systematic Reviews 2020 (4) Study design
184. Folate supplementation in people with sickle cell disease Cochrane Database of Systematic Reviews 2018 (3) Population
185. Combining weight-loss counseling with the Weight Watchers plan for obese breast cancer survivors Obesity Research 2002 10(7)657--665 Population
186. Randomized controlled trial of a patient decision aid for colorectal cancer screening. Medical decision making : an international journal of the Society for Medical Decision Making 2002 22(2)125--139 Population
187. The Effectiveness of a Physician-Only and Physician-Patient Intervention on Colorectal Cancer Screening Discussions Between Providers and African American and Latino Patients. JGIM: Journal of General Internal Medicine Dec 2015 30(12)1780--1787 Intervention
188. Designing an intervention to help people with colorectal adenomas reduce their intake of red and processed meat and increase their levels of physical activity: a qualitative study. BMC cancer 2012 12()255 Population
189. Sensory environment on healthâ€ related outcomes of hospital patients Cochrane Database of Systematic Reviews 2012 (3) Study design
190. Whole fruits and fruit fiber emerging health effects Nutrients 2018 10(12) Outcome
191. Increasing preventive health services via tailored health communications. The American journal of managed care Oct 2014 20(10)828--835 Outcome
192. Interventions for the treatment of Paget's disease of the vulva Cochrane Database of Systematic Reviews 2019 (6) Study design
193. Lessons learned: Outcomes and methodology of a coping skills intervention trial comparing individual and group formats for patients with cancer International Journal of Psychiatry in Medicine 2001 31(3)289--304 Outcome
194. Personalised risk communication for informed decision making about taking screening tests Cochrane Database of Systematic Reviews 2013 (2) Study design
195. Optimal primary surgical treatment for advanced epithelial ovarian cancer Cochrane Database of Systematic Reviews 2011 (8) Study design
196. Fe en Acci'on/Faith in Action: promotion of cancer screening among churchgoing Latinas Cancer epidemiology biomarkers and prevention Population
197. Riskâ€ reducing bilateral salpingoâ€ oophorectomy in women with BRCA1 or BRCA2 mutations Cochrane Database of Systematic Reviews 2018 (8) Population
198. Designing a randomized controlled trial to evaluate a community-based narrative intervention for improving colorectal cancer screening for African Americans. Contemporary clinical trials Feb 2018 65()8--18 Intervention
199. Diet and Irritable Bowel Syndrome, with a Focus on Appetite-Regulating Gut Hormones 2014 ()5--16 Population
200. American Cancer Society Colorectal Cancer Survivorship Care Guidelines CA Cancer Journal for Clinicians 2015 Study design

201. Patient Beliefs About Colon Cancer Screening. Journal of cancer education : the official journal of the American Association for Cancer Education Mar 2016 31(1)39--46 Outcome
202. The Improving Rural Cancer Outcomes (IRCO) Trial: a factorial cluster-randomised controlled trial of a complex intervention to reduce time to diagnosis in rural patients with cancer in Western Australia: a study protocol. BMJ open Sep 2014 4(9)e006156 Study design
203. Tailored computer-based cancer risk communication: Correcting colorectal cancer risk perception Journal of Health Communication Mar 2004 9(2)127--141 Intervention
204. Project PREVENT: a randomized trial to reduce multiple behavioral risk factors for colon cancer. Cancer epidemiology, biomarkers {\&} prevention : a publication of the American Association for Cancer Research, cosponsored by the American Society of Preventive Oncology Jun 2005 14(6)1453--1459 Intervention
205. Nutrition and diagnosis-related care: Eighth edition 2015 ()1--1038 Study design
206. New hypotheses for the health-protective mechanisms of whole-grain cereals: What is beyond fibre? Nutrition Research Reviews 2010 23(1)65--134 Outcome
207. High calorie diet and the human brain: Metabolic consequences of long-term consumption 2015 ()1--308 Intervention
208. Highâ€ dose chemotherapy and autologous bone marrow or stem cell transplantation versus conventional chemotherapy for women with early poor prognosis breast cancer Cochrane Database of Systematic Reviews 2016 (5) Study design
209. Screening flexible sigmoidoscopy using an upper endoscope is better tolerated by women. The American journal of gastroenterology Jun 2004 99(6)1074--1080 Population
210. Effects of legume kernel fibres and citrus fibre on putative risk factors for colorectal cancer: a randomised, double-blind, crossover human intervention trial Nutrition Journal 2013 12() Intervention
211. Maximizing patient adherence to prehabilitation: what do the patients say? Supportive Care in Cancer 2018 26(8)2717--2723 Outcome
212. Corticosteroids for the resolution of malignant bowel obstruction in advanced gynaecological and gastrointestinal cancer Cochrane Database of Systematic Reviews 2000 (1) Outcome
213. Brain-gut interactions in IBS Frontiers in Pharmacology 2012 3 Population
214. Celecoxib for rheumatoid arthritis Cochrane Database of Systematic Reviews 2017 (6) Population
215. Time management in radiation oncology: development and evaluation of a modular system based on the example of rectal cancer treatment The DEGRO-QUIRO trial Strahlentherapie Und Onkologie 2012 188(1)5--11 Outcome
216. Get screened: a pragmatic randomized controlled trial to increase mammography and colorectal cancer screening in a large, safety net practice. BMC health services research Sep 2010 10()280 Population
217. Patient navigation for breast and colorectal cancer treatment: a randomized trial. Cancer epidemiology, biomarkers {\&} prevention : a publication of the American Association for Cancer Research, cosponsored by the American Society of Preventive Oncology Oct 2012 21(10)1673--1681 Outcome
218. Stem cell therapy for chronic ischaemic heart disease and congestive heart failure Cochrane Database of Systematic Reviews 2016 (12) Study design
219. The impact of social deprivation on the response to a randomised controlled trial of a weight management intervention (BeWEL) for people at increased risk of colorectal cancer. Journal of Human Nutrition {\&} Dietetics Jun 2018 31(3)306--313 Population
220. Process evaluation in an intervention designed to improve rates of colorectal cancer screening in a VA medical center. Health promotion practice Jul 2007 8(3)273--281 Population
221. Minority stress and leukocyte gene expression in sexual minority men living with treated HIV infection Brain Behavior and Immunity 2018 70()335--345 Population
222. Interventions for improving uptake of populationâ€ based screening for colorectal cancer using fecal occult blood testing Cochrane Database of Systematic Reviews 2004 (4) Study design
223. Interactive telemedicine: effects on professional practice and health care outcomes Cochrane Database of Systematic Reviews 2015 (9) Study design
224. A comparison of physical activity correlates across breast, prostate and colorectal cancer survivors in Nova Scotia, Canada. Supportive care in cancer : official journal of the Multinational Association of Supportive Care in Cancer Apr 2014 22(4)891--903 Outcome
225. Prevalence and Correlates of Strength Exercise Among Breast, Prostate, and Colorectal Cancer Survivors. Oncology Nursing Forum Mar 2015 42(2)118--127 Outcome
226. Coaching intervention as a strategy for minority recruitment to cancer clinical trials. Journal of oncology practice Nov 2013 9(6)294--299 Intervention
227. Pharmacological interventions for treating chronic prostatitis/chronic pelvic pain syndrome Cochrane Database of Systematic Reviews 2019 (10) Study design

228. An allied health rehabilitation program for patients following surgery for abdomino-pelvic cancer: a feasibility and pilot clinical study Supportive Care in Cancer 2020 28(3)1335--1350 Intervention
229. The effects of two health information texts on patient recognition memory: a randomized controlled trial. Patient education and counseling Aug 2013 92(2)260--265 Intervention
230. Lymphadenectomy for the management of endometrial cancer Cochrane Database of Systematic Reviews 2017 (10) Study design
231. Primary Outcomes of a Randomized Controlled Crossover Trial to Explore the Effects of a High Chlorophyll Dietary Intervention to Reduce Colon Cancer Risk in Adults: the Meat and Three Greens (M3G) Feasibility Trial Nutrients 2019 11(10) Intervention
232. A randomised controlled trial of a case management approach to encourage participation in colorectal cancer screening for people with schizophrenia in psychiatric outpatient clinics: study protocol for the J-SUPPORT 1901 (ACCESS) study. BMJ open Nov 2019 9(11)e032955 Population
233. Impact of a cancer education seminar on knowledge and screening intent among Chinese Americans: Results from a randomized, controlled, community-based trial. Cancer Apr 2018 124 Suppl()1622--1630 Intervention
234. Impact of longâ€ term opioid use for chronic nonâ€ cancer pain on misuse, abuse or addiction, overdose, falls and fractures Cochrane Database of Systematic Reviews 2014 (4) Study design
235. The LEAD trial - the effectiveness of a decision aid on decision making among citizens with lower educational attainment who have not participated in FIT-based colorectal cancer screening in Denmark: study protocol for a randomized controlled trial. Trials Oct 2018 19(1)543 Population
236. The LEAD trialâ€"The effectiveness of a decision aid on decision making among citizens with lower educational attainment who have not participated in FIT-based colorectal cancer screening in Denmark: A randomised controlled trial Patient Education and Counseling 2020 103(2)359--368 Population
237. Cytoreductive surgery plus chemotherapy versus chemotherapy alone for recurrent epithelial ovarian cancer Cochrane Database of Systematic Reviews 2010 (6) Study design
238. Telehealth System: A Randomized Controlled Trial Evaluating the Impact of an Internet-Based Exercise Intervention on Quality of Life, Pain, Muscle Strength, and Fatigue in Breast Cancer Survivors Cancer 2016 122(20)3166--3174 Population
239. Patient satisfaction and health-related quality of life after treatment for colon cancer Coloproctology 2007 29(5)247--257 Outcome
240. Comparative effectiveness of next generation genomic sequencing for disease diagnosis: design of a randomized controlled trial in patients with colorectal cancer/polyposis syndromes. Contemporary clinical trials Sep 2014 39(1)1--8 Outcome
241. Psychosocial interventions to improve quality of life and emotional wellbeing for recently diagnosed cancer patients Cochrane Database of Systematic Reviews 2012 (11) Study design
242. Traditional Chinese Medicine herbs for stopping bleeding from haemorrhoids Cochrane Database of Systematic Reviews 2010 (10) Study design
243. Reduced pain during screening colonoscopy with an ultrathin colonoscope: a randomized controlled trial. Endoscopy Aug 2012 44(8)740--746 Population
244. Helminth therapy (worms) for induction of remission in inflammatory bowel disease Cochrane Database of Systematic Reviews 2014 (1) Study design
245. Primary prevention of colorectal cancer: Diet and drugs Gastroenterology Clinics of North America 2002 () Study design
246. Combination of perianal-intrarectal lidocaine-prilocaine cream and periprostatic nerve block for pain control during transrectal ultrasound guided prostate biopsy: a randomized, controlled trial. The Journal of urology Feb 2009 181(2)583--585 Outcome
247. Prehabilitation with Whey Protein Supplementation on Perioperative Functional Exercise Capacity in Patients Undergoing Colorectal Resection for Cancer: A Pilot Double-Blinded Randomized Placebo-Controlled Trial. Journal of the Academy of Nutrition and Dietetics 2016 116(5)802--812 Intervention
248. Impact of an educational video-based strategy on the behavior process associated with colorectal cancer screening: a randomized controlled study. Cancer epidemiology Oct 2009 33(44259)216--222 Intervention
249. Azathioprine and 6â€ mercaptopurine for maintenance of surgicallyâ€ induced remission in Crohn's disease Cochrane Database of Systematic Reviews 2019 (8) Intervention
250. Management for intussusception in children Cochrane Database of Systematic Reviews 2017 (6) Study design
251. The impact of physical activity on disease-free survival in patients with high-risk stage ii or III colon cancer: one month screening results Asia-pacific journal of clinical oncology 2012 8()345-- Outcome
252. Nursing education as an intervention to decrease fatigue perception in oncology patients. European journal of oncology nursing : the official journal of European Oncology Nursing Society Apr 2006 10(2)150--155 Outcome

253.	Psychosocial interventions for reducing fatigue during cancer treatment in adults Cochrane Database of Systematic Reviews 2009 (1) Study design
254.	Managing Multi-Center Recruitment in the PLCO Cancer Screening Trial. Reviews on recent clinical trials 2015 10(3)187--193 Outcome
255.	The PLCO Cancer Screening Trial: Background, Goals, Organization, Operations, Results. Reviews on recent clinical trials 2015 10(3)173--180 Population
256.	Development of an e-health physical activity intervention for prostate and colorectal cancer survivors: the oncoactive+ intervention Psycho-oncology 2014 23 CC -()258-- Outcome
257.	Development of a computer-tailored physical activity intervention for prostate and colorectal cancer patients and survivors: OncoActive. BMC cancer Jun 2017 17(1)446 Study design
258.	Telephone, print, and Web-based interventions for physical activity, diet, and weight control among cancer survivors: a systematic review Journal of Cancer Survivorship 2015 9(4)660--682 Study design
259.	Clinical trials of antioxidants as cancer prevention agents: Past, present, and future Free Radical Biology and Medicine 2011 51(5)1068--1084 Outcome
260.	A telephoneâ€ delivered multiple health behaviour change intervention for colorectal cancer survivors: making the case for costâ€ effective healthcare European journal of cancer care Outcome
261.	Review of the health effects of berries and their phytochemicals on the digestive and immune systems Nutrition Reviews 2018 76(1)29--46 Outcome
262.	Nonâ€ steroidal antiâ€ inflammatory agents to induce regression and prevent the progression of cervical intraepithelial neoplasia Cochrane Database of Systematic Reviews 2018 (2) Study design
263.	Rural-urban differences in health behaviors and outcomes among older, overweight, long-term cancer survivors in the RENEW randomized control trial. Cancer Causes {\&} Control: CCC Feb 2019 Outcome
264.	Systems of support to increase colorectal cancer screening and follow-up rates (SOS): design, challenges, and baseline characteristics of trial participants. Contemporary clinical trials Nov 2010 31(6)589--603 Population
265.	Implementation intentions and colorectal screening: a randomized trial in safety-net clinics. American Journal of Preventive Medicine Dec 2014 47(6)703--714 Population
266.	Feasibility randomised controlled trial of a guided workbook intervention to support work-related goals among cancer survivors in the UK BMJ open 2019 9(1)e022746 Intervention
267.	Alerts in electronic medical records to promote a colorectal cancer screening programme: a cluster randomised controlled trial in primary care. The British journal of general practice : the journal of the Royal College of General Practitioners Jul 2016 66(648)e483--90 Population
268.	Laparoscopic versus open transhiatal oesophagectomy for oesophageal cancer Cochrane Database of Systematic Reviews 2016 (3) Study design
269.	A culturally and linguistically salient pilot intervention to promote colorectal cancer screening among Latinos receiving care in a Federally Qualified Health Center Health Education Research 2019 34(3)310--320 Population
270.	The effect of test kit provision, and individual and family education on the uptake rates of fecal occult blood test in an Asian population: a randomized controlled trial. Cancer causes {\&} control : CCC Nov 2014 25(11)1473--1488 Population
271.	Water infusion versus air insufflation for colonoscopy Cochrane Database of Systematic Reviews 2015 (5) Study design
272.	Harnessing the Question-Behavior Effect to Enhance Colorectal Cancer Screening in an mHealth Experiment. American Journal of Public Health Nov 2016 106(11)1998--2004 Population
273.	OncoArray: Prostate Cancer Database of Genotypes and Phenotypes: dbGaP 2017 Population
274.	Desmopressin for treating nocturia in men Cochrane Database of Systematic Reviews 2017 (10) Study design
275.	HealthLinks randomized controlled trial: Design and baseline results. Contemporary clinical trials May 2016 48()1--11 Study design
276.	Lateral pararectal versus transrectal stoma placement for prevention of parastomal herniation Cochrane Database of Systematic Reviews 2019 (4) Study design
277.	The effect on compliance of a health education leaflet in colorectal cancer screening in general practice in central England. Journal of epidemiology and community health Apr 1997 51(2)187--191 Population
278.	Shared Decision Making and the Use of Decision Aids Deutsches Arzteblatt international Outcome
279.	Omegaâ€ 3 polyunsaturated fatty acids (PUFA) for type 2 diabetes mellitus Cochrane Database of Systematic Reviews 2008 (1) Study design
280.	The National Institute on Aging and the National Cancer Institute SEER collaborative study on comorbidity and early diagnosis of cancer in the elderly Cancer 1994 74(7 Suppl CC - SR-CANCER CC - Colorectal CC - Gut)2101--2106 Population
281.	Developing and pilot testing a telephone-delivered lifestyle intervention for colorectal cancer survivors - 'Canchange' Asia-Pacific journal of clinical oncology 2009 5()A215 Study design

282. 'Canchange': a trial of a telephone-delivered lifestyle intervention for colorectal cancer (CRC) survivors Asia-Pacific journal of clinical oncology 2010 6()193 Outcome
283. A randomised controlled trial of the effects of a telephone-delivered program on health behaviours and quality of life for colorectal cancer survivors ('canchange') Asia-pacific journal of clinical oncology 2011 7()80--81 Outcome
284. Effects of a multiple health behavior change intervention for colorectal cancer survivors on psychosocial outcomes and quality of life: a randomized controlled trial. Annals of behavioral medicine : a publication of the Society of Behavioral Medicine Dec 2014 48(3)359--70 Outcome
285. Emerging aspects of food and nutrition on gut microbiota Journal of Agricultural and Food Chemistry 2013 61(40)9559--9574
286. Emerging aspects of food and nutrition on gut microbiota Journal of Agricultural and Food Chemistry 2013 61(40)9559--9574 Study design
287. Primary care nutrition: Writing the nutrition prescription 2017 ()1--370 Study design
288. A Systematic Review and Meta-analysis of Physical Exercise Prehabilitation in Major Abdominal Surgery (PROSPERO 2017 CRD42017080366). Journal of gastrointestinal surgery : official journal of the Society for Surgery of the Alimentary Tract Jun 2020 24(6)1375--1385 Study design
289. Diet, physical activity or both for prevention or delay of type 2 diabetes mellitus and its associated complications in people at increased risk of developing type 2 diabetes mellitus Cochrane Database of Systematic Reviews 2017 (12) Study design
290. Study protocol: a randomized controlled trial of patient navigation-activation to reduce cancer health disparities. BMC cancer Oct 2010 10()551 Intervention
291. Randomized, controlled trial of a multimodal intervention to improve cancer screening rates in a safety-net primary care practice. Journal of general internal medicine Jan 2014 29(1)41--49 Population
292. The Norwegian dietary guidelines and colorectal cancer survival (CRC-NORDIET) study: a food-based multicentre randomized controlled trial. BMC cancer 2017 (1)83 Intervention
293. Ivermectin versus albendazole or thiabendazole for Strongyloides stercoralis infection Cochrane Database of Systematic Reviews 2016 (1) Study design
294. Early enteral nutrition within 24 hours of lower gastrointestinal surgery versus later commencement for length of hospital stay and postoperative complications Cochrane Database of Systematic Reviews 2019 (7) Study design
295. Total intravenous anaesthesia versus inhalational anaesthesia for adults undergoing transabdominal robotic assisted laparoscopic surgery Cochrane Database of Systematic Reviews 2017 (4) Study design
296. Screening for colorectal cancer using the faecal occult blood test, Hemoccult Cochrane Database of Systematic Reviews 2007 (1) Study design
297. Hypofractionation for clinically localized prostate cancer Cochrane Database of Systematic Reviews 2019 (9) Study design
298. Socio-ecological correlates of physical activity in breast and colon cancer survivors 4 years after participation in a randomized controlled exercise trial (PACT study) PloS one 2020 15(4)e0231663 Outcome
299. Coffee and health: A review of recent human research Critical Reviews in Food Science and Nutrition 2006 46(2)101--123 Study design
300. Feasibility and efficacy of pairing fecal immunochemical testing with mammography for increasing colorectal cancer screening among uninsured Latinas in northern Manhattan Preventive Medicine 2011 53(3)194--198 Population
301. Tailored message interventions versus typical messages for increasing participation in colorectal cancer screening among a non-adherent population: A randomized controlled trial BMC Public Health May 2016 16()431 Population
302. Study protocol for Moving Bright Eating Smart"- A phase 2 clinical trial on the acceptability and feasibility of a diet and physical activity intervention to prevent recurrence in colorectal cancer survivors." 1 487 (13)jan Study design
303. Study protocol on comparative effectiveness of mindfulness meditation and qigong on psychophysiological outcomes for patients with colorectal cancer: a randomized controlled trial. BMC complementary and alternative medicine 2017 17(1)390 Study design
304. The Mediterranean Diet: Health and Science 2013 1--402 Study design
305. An entertainment-education colorectal cancer screening decision aid for African American patients: A randomized controlled trial. Cancer Apr 2017 123(8)1401--1408 Population
306. Spiritually based intervention to increase colorectal cancer screening among African Americans: screening and theory-based outcomes from a randomized trial. Health education {\&} behavior : the official publication of the Society for Public Health Education Aug 2013 40(4)458--468 Population
307. Effect of patient navigation on colorectal cancer screening in a community-based randomized controlled trial of urban African American adults. Cancer causes {\&} control : CCC Feb 2015 26(2)239--246 Population
308. Mistletoe therapy in oncology Cochrane Database of Systematic Reviews 2008 (2) Study design

309. Electrical stimulation for faecal incontinence in adults Cochrane Database of Systematic Reviews 2007 (3) Study design
310. Efficacy, cost-utility and reach of an eHealth self-management application 'Oncokompas' that helps cancer survivors to obtain optimal supportive care: study protocol for a randomised controlled trial Trials 2017 18(1) Study design
311. Follow-up after rectal cancer: developing and testing a novel patient-led follow-up program. Study protocol. Acta oncologica (Stockholm, Sweden) Feb 2017 56(2)307--313 Study design
312. Peer Support as an Ideal Solution for Racial/Ethnic Disparities in Colorectal Cancer Screening: Evidence from a Systematic Review and Meta-analysis. Diseases of the colon and rectum Jun 2020 63(6)850--858 Study design
313. Cancer prevention for working class, multiethnic populations through small businesses: the healthy directions study Cancer Causes {\&} Control 2003 14(8)749--760 Intervention
314. Online Narratives and Peer Support for Colorectal Cancer Screening A Pilot Randomized Trial American Journal of Preventive Medicine 2013 45(1)98--107 Population
315. Naftopidil for the treatment of lower urinary tract symptoms compatible with benign prostatic hyperplasia Cochrane Database of Systematic Reviews 2018 (10) Population
316. Followâ€ up strategies following completion of primary cancer treatment in adult cancer survivors Cochrane Database of Systematic Reviews 2019 17(11) Study design
317. No change in health behavior, BMI or self-rated health after a psychosocial cancer rehabilitation: Results of a randomized trial Acta Oncologica 2011 50(2)289--298 Intervention
318. Low rectal cancer: a call for a change of approach in abdominoperineal resection. Journal of Clinical Oncology Dec 2005 23(36)9257--9264 Intervention
319. Interventions for maintenance of surgically induced remission in Crohn's disease: a network metaâ€ analysis Cochrane Database of Systematic Reviews 2019 (9) Study design
320. Screening for prostate cancer Cochrane Database of Systematic Reviews 2013 (1) Study design
321. Fecal transplantation for treatment of inflammatory bowel disease Cochrane Database of Systematic Reviews 2018 (11) Outcome
322. Colonoscopy uptake for high-risk individuals with a family history of colorectal neoplasia: A multicenter, randomized trial of tailored counseling versus standard information. Medicine Aug 2016 95(33)e4303 Intervention
323. [Cancer prevention in familial cancer]. Gan to kagaku ryoho. Cancer {\&} chemotherapy Apr 2002 29(4)545--549 Study design
324. Impact of transrectal ultrasound guided prostate biopsy on quality of life: A prospective randomized trial comparing 6 versus 12 cores JOURNAL OF UROLOGY Jan 2001 165(1)100--103 Population
325. Preventing cancer: dietary lifestyle or clinical intervention? ASIA PACIFIC JOURNAL OF CLINICAL NUTRITION 2002 11(S)S618--S631 Intervention
326. Skin toxicities associated with epidermal growth factor receptor inhibitors TARGETED ONCOLOGY Apr 2009 4(2)107--119 Population
327. Long-Term Quality of Life After Radiotherapy for the Treatment of Anal Cancer CANCER Feb 2010 116(4)822--829 Outcome
328. Evaluation of follow-up strategies for patients with epithelial ovarian cancer following completion of primary treatment COCHRANE DATABASE OF SYSTEMATIC REVIEWS 2011 (6) Study design
329. Definition of supportive care: does the semantic matter? CURRENT OPINION IN ONCOLOGY Jul 2014 26(4)372--379 Study design
330. A randomized pilot trial of a telephone-based couples intervention for physical intimacy and sexual concerns in colorectal cancer PSYCHO-ONCOLOGY Sep 2014 23(9)1005--1013 Outcome
331. Current Practice and Barriers in Cancer Rehabilitation: Perspectives of Korean Physiatrists CANCER RESEARCH AND TREATMENT Jul 2015 47(3)370--378 Outcome
332. Exercise improves quality of life in patients with cancer: a systematic review and meta-analysis of randomised controlled trials BRITISH JOURNAL OF SPORTS MEDICINE Jul 2016 50(13)796--803 Study design
333. Oncology care provider perspectives on exercise promotion in people with cancer: an examination of knowledge, practices, barriers, and facilitators SUPPORTIVE CARE IN CANCER Jul 2017 25(7)2297--2304 Outcome
334. Mammography screening: A major issue in medicine EUROPEAN JOURNAL OF CANCER Feb 2018 90()34--62 Population
335. Which exercise prescriptions improve quality of life and physical function in patients with cancer during and following treatment? A systematic review and meta-analysis of randomised controlled trials BRITISH JOURNAL OF SPORTS MEDICINE Apr 2018 52(8)505+ Outcome
336. Feasibility and preliminary efficacy of an exercise telephone counseling intervention for hematologic cancer survivors: a phase II randomized controlled trial JOURNAL OF CANCER SURVIVORSHIP Jun 2018 12(3)357--370 Population

337. Cognitively-Based Compassion Training versus cancer health education to improve health-related quality of life in survivors of solid tumor cancers and their informal caregivers: study protocol for a randomized controlled pilot trial TRIALS Apr 2019 20() Outcome
338. Exercise training, circulating cytokine levels and immune function in cancer survivors: A meta-analysis BRAIN BEHAVIOR AND IMMUNITY Oct 2019 81()92--104 Study design
339. Exercise intervention for post-treatment colorectal cancer survivors: a systematic review and meta-analysis JOURNAL OF CANCER SURVIVORSHIP Dec 2020 14(6)878--893 Study design
340. Oestrogens for treatment or prevention of pelvic organ prolapse in postmenopausal women Cochrane Database of Systematic Reviews 2010 (9) Study design
341. Prehabilitation Prior to Major Cancer Surgery: Training for Surgery to Optimize Physiologic Reserve to Reduce Postoperative Complications Current Anesthesiology Reports 2018 8(4)375--385 Study design
342. Evaluation of intervention strategies to manage fatigue during active treatment and to prevent persistent fatigue after curative treatment for cancer http://www.who.int/trialsearch/Trial2.aspx?TrialID=ISRCTN20583070 2007 Study design
343. Mailed Outreach Is Superior to Usual Care Alone for Colorectal Cancer Screening in the USA: A Systematic Review and Meta-analysis. Digestive diseases and sciences Sep 2019 64(9)2489--2496 Population
344. Directâ€ acting antivirals for chronic hepatitis C Cochrane Database of Systematic Reviews 2017 (9) Study design
345. Health behavior correlates among colon cancer survivors: NCSTRIDES baseline results American Journal of Health Behavior 2006 30(6)720--730 Intervention
346. Culturally targeted patient navigation for increasing african americans' adherence to screening colonoscopy: a randomized clinical trial. Cancer epidemiology, biomarkers {\&} prevention : a publication of the American Association for Cancer Research, cosponsored by the American Society of Preventive Oncology Sep 2013 22(9)1577--1587 Population
347. Implementation of culturally targeted patient navigation system for screening colonoscopy in a direct referral system. Health education research Oct 2013 28(5)803--815 Population
348. Multimodal prehabilitation to reduce the incidence of delirium and other adverse events in elderly patients undergoing elective major abdominal surgery: An uncontrolled before-and-after study Plos One 2019 14(6) Outcome
349. Healthy and unhealthy dietary patterns and the risk of chronic disease: an umbrella review of meta-analyses of prospective cohort studies British Journal of Nutrition 2020 124(11)1133--1144 Study design
350. Program to improve colorectal cancer screening in a low-income, racially diverse population: a randomized controlled trial. Annals of family medicine 2012 10(5)412--417 Population
351. Followâ€ up strategies for patients treated for nonâ€ metastatic colorectal cancer Cochrane Database of Systematic Reviews 2019 (9) Outcome
352. Evaluating a nurse-led survivorship care package (SurvivorCare) for bowel cancer survivors: study protocol for a randomized controlled trial. Trials Aug 2013 14()260 Intervention
353. SCORE: shared care of Colorectal cancer survivors: protocol for a randomised controlled trial Trials 2017 18(1)506 Study design
354. Assessing Acceptability of a Diagnostic and Malaria Treatment Package Delivered by Community Health Workers in Malaria-Endemic Settings of Burkina Faso, Nigeria, and Uganda Clinical Infectious Diseases 2016 63()S306--S311 Outcome
355. Physical activity in colorectal cancer patients: a review of clinical trials Journal of cancer research and clinical oncology 2012 138()129-- Population
356. Prehabilitation for major abdominal urologic oncology surgery Current Opinion in Urology 2018 28(3)243--250 Study design
357. Effects of a tailored interactive multimedia computer program on determinants of colorectal cancer screening: a randomized controlled pilot study in physician offices. Patient education and counseling Apr 2007 66(1)67--74 Population
358. The effects of tailoring knowledge acquisition on colorectal cancer screening self-efficacy Journal of Health Communication Jun 2015 20(6)697--709 Population
359. A Cluster Randomized Controlled Trial of a Lay Health Worker Intervention to Increase Healthy Eating and Physical Activity Among Vietnamese Americans Preventing Chronic Disease 2020 17 Population
360. Decision Aid to Technologically Enhance Shared decision making (DATES): study protocol for a randomized controlled trial. Trials 2013 14()381 Population
361. Perceived Barriers and Facilitators of Using a Web-Based Interactive Decision Aid for Colorectal Cancer Screening in Community Practice Settings: Findings From Focus Groups With Primary Care Clinicians and Medical Office Staff Journal of Medical Internet Research 2013 15(12) Outcome

362. Dietary flavonoid for preventing colorectal neoplasms Cochrane Database of Systematic Reviews 2012 (8) Study design
363. Lay health educators and print materials for the promotion of colorectal cancer screening among Korean Americans: A randomized comparative effectiveness study. Cancer Jul 2017 123(14)2705--2715 Population
364. Lupin kernel fibre foods improve bowel function and beneficially modify some putative faecal risk factors for colon cancer in men British journal of nutrition 2006 95(2)372--378 Intervention
365. Transcutaneous electrical nerve stimulation for acute pain Cochrane Database of Systematic Reviews 2015 (6) Study design
366. Diet, exercise, and complementary therapies after primary treatment for cancer Lancet Oncology 2006 7(12)1017--1026 Study design
367. Pain management for women in labour: an overview of systematic reviews Cochrane Database of Systematic Reviews 2012 (3) Study design
368. Randomized clinical trial on enhanced recovery versus standard care following open liver resection British journal of surgery 2013 100(8)1015--1024 Intervention
369. Interventions for promoting physical activity in people with neuromuscular disease Cochrane Database of Systematic Reviews 2020 (3) Study design
370. Use of irinotecan combined with 5-fluorouracil and leucovorin as first-line therapy for metastatic colorectal cancer Current oncology (Toronto, Ont.) 2001 8(2)60--68 Study design
371. Preoperative mechanical preparation of the colon: the patient's experience. BMC surgery May 2007 7()5 Intervention
372. Effect of Coffee Added to a Polyethylene glycol plus Ascorbic acid Solution for Bowel Preparation prior to Colonoscopy. Journal of gastrointestinal and liver diseases : JGLD Mar 2016 25(1)63--69 Population
373. Interventions for treating collagenous colitis Cochrane Database of Systematic Reviews 2017 (11) Study design
374. Cannabis for the treatment of Crohn's disease Cochrane Database of Systematic Reviews 2018 (11) Study design
375. Combined intermittent pneumatic leg compression and pharmacological prophylaxis for prevention of venous thromboembolism Cochrane Database of Systematic Reviews 2016 (9) Study design
376. Reduced length of stay and convalescence in laparoscopic vs open sigmoid resection with traditional care: a double blinded randomized clinical trial Colorectal Disease Outcome
377. The NordICC Study: rationale and design of a randomized trial on colonoscopy screening for colorectal cancer. Endoscopy Jul 2012 44(7)695--702 Population
378. Diet and lifestyle intervention among patients with colorectal adenomas: rationale and design of a Malaysian study. Asian Pacific journal of cancer prevention : APJCP 2005 Population
379. Management of long-term colorectal cancer survivors in Korea Journal of the Korean Medical Association 2016 59(4)276--286 Intervention
380. Feasibility of preoperative supervised home-based exercise in older adults undergoing colorectal cancer surgery - A randomized controlled design PloS one 2019 14(7)e0219158 Outcome
381. Evaluation of Online Learning Modules for Improving Physical Activity Counseling Skills, Practices, and Knowledge of Oncology Nurses Oncology Nursing Forum 2017 44(6)729--738 Outcome
382. Quality of life and dietary changes among cancer patients: A systematic review Quality of Life Research: An International Journal of Quality of Life Aspects of Treatment, Care {\&} Rehabilitation Mar 2015 24(3)705--719 Study design
383. Patient activation increases colorectal cancer screening rates: a randomized trial among low-income minority patients. Cancer epidemiology, biomarkers {\&} prevention : a publication of the American Association for Cancer Research, cosponsored by the American Society of Preventive Oncology Jan 2012 21(1)45--52 Population
384. Assessing Colorectal Cancer Screening Barriers by Two Methods. Journal of cancer education : the official journal of the American Association for Cancer Education Jun 2018 33(3)536--543 Population
385. Voglibose for prevention of type 2 diabetes mellitus: a randomised, double-blind trial in Japanese individuals with impaired glucose tolerance Lancet (london, england) 2009 373(9675)1607--1614 Population
386. Comparison of routes for achieving parenteral access with a focus on the management of patients with Ebola virus disease Cochrane Database of Systematic Reviews 2015 (2) Study design
387. A patient initiated computer program improved breast cancer screening practices in primary care [commentary on Williams RB, Boles M, Johnson RE. A patient-initiated system for preventive health care. A randomized trial in community-based primary care prac Evidence Based Nursing 1999 Population
388. Physical activity, sedentary behaviour, diet, and cancer: an update and emerging new evidence. The Lancet. Oncology Aug 2017 18(8)e457--e471 Study design

389. Improving uptake of flexible sigmoidoscopy screening: a randomized trial of nonparticipant reminders in the English Screening Programme. Endoscopy Jan 2017 49(1)35--43 Population
390. Dietary Interventions 2006 (11) Study design
391. Ghrelin for the management of cachexia associated with cancer Cochrane Database of Systematic Reviews 2018 (2) Study design
392. Nerveâ€ sparing radical hysterectomy compared to standard radical hysterectomy for women with early stage cervical cancer (stage Ia2 to IIa) Cochrane Database of Systematic Reviews 2019 (2) Study design
393. Conjugated Linoleic Acid: Potential Health Benefits as a Functional Food Ingredient Annual Review of Food Science and Technology 2016 7()221--244 Study design
394. Acupuncture and PC6 stimulation for the prevention of postoperative nausea and vomiting in patients undergoing elective laparoscopic resection of colorectal cancer: a study protocol for a three-arm randomised pilot trial. BMJ open Jan 2017 7(1)e013457 Outcome
395. Association of Body Esteem with Fitness and Body Fat Among Colorectal Cancer Survivors: secondary Analyses from a Randomized Trial International journal of behavioral medicine 2019 26(6)619--628 Outcome
396. Evaluation of the addition of video-based education for patients receiving standard pre-chemotherapy education. European journal of cancer care Jul 2008 17(4)328--339 Intervention
397. Dexmedetomidine for conscious sedation with colorectal endoscopic submucosal dissection: a prospective double-blind randomized controlled study. Clinical and translational gastroenterology Jul 2018 9(7)167 Intervention
398. Prospective study of fruit and vegetable intake and risk of prostate cancer Jnci-Journal of the National Cancer Institute 2007 99(15)1200--1209 Population
399. Design of a randomized clinical trial of a colorectal cancer screening decision aid to promote appropriate screening in community-dwelling older adults. Clinical Trials Dec 2017 14(6)648--658 Population
400. Interventions for weight reduction in obesity to improve survival in women with endometrial cancer Cochrane Database of Systematic Reviews 2018 (2) Intervention
401. Aerobic physical exercise for adult patients with haematological malignancies Cochrane Database of Systematic Reviews 2019 (1) Population
402. Changes in health behavior 1 year after testing negative at a colorectal cancer screening: a randomized-controlled study. European Journal Of Cancer Prevention: The Official Journal Of The European Cancer Prevention Organisation (ECP) Jul 2018 27(4)316--322 Population
403. Improving Cancer Preventive Behaviors: A Randomized Trial of Tailored Lifestyle Feedback in Colorectal Cancer Screening. Cancer epidemiology, biomarkers {\&} prevention : a publication of the American Association for Cancer Research, cosponsored by the American Society of Preventive Oncology 2018 27(12)1442--1449 Population
404. Understanding quality of life in patients with colorectal cancer: comparison of data from a randomised controlled trial, a population based cohort study and the norm reference population Inflammation Research 2004 64()S130--S135 Outcome
405. Diet and exercise in uterine cancer survivors (DEUS pilot) - piloting a healthy eating and physical activity program: study protocol for a randomized controlled trial Trials 2016 17 Population
406. Improving Multiple Behaviors for Colorectal Cancer Prevention Among African American Church Members Health Psychology 2004 23(5)492--502 Population
407. General health checks in adults for reducing morbidity and mortality from disease Cochrane Database of Systematic Reviews 2019 (1) Study design
408. A Media and Clinic Intervention to Increase Colorectal Cancer Screening in Ohio Appalachia. BioMed research international 2015 2015()943152 Population
409. Nonâ€ steroidal antiâ€ inflammatory drugs (NSAIDs) for axial spondyloarthritis (ankylosing spondylitis and nonâ€ radiographic axial spondyloarthritis) Cochrane Database of Systematic Reviews 2015 (7) Study design
410. Renewed: protocol for a randomised controlled trial of a digital intervention to support quality of life in cancer survivors BMJ Open 2019 Study design
411. Palliative surgery versus medical management for bowel obstruction in ovarian cancer Cochrane Database of Systematic Reviews 2010 (7) Study design
412. Budesonide for maintenance of remission in Crohn's disease Cochrane Database of Systematic Reviews 2014 (8) Study design
413. Intraâ€ peritoneal prophylactic agents for preventing adhesions and adhesive intestinal obstruction after nonâ€ gynaecological abdominal surgery Cochrane Database of Systematic Reviews 2009 (1) Study design
414. Role of somatostatin in the regulation of central and peripheral factors of satiety and obesity International Journal of Molecular Sciences 2020 21(7) Outcome

415.	Dietary fiber intake and risk of colorectal cancer and incident and recurrent adenoma in the Prostate, Lung, Colorectal, and Ovarian Cancer Screening Trial. The American journal of clinical nutrition Oct 2015 102(4)881--890 Outcome
416.	Feasibility of a patient-controlled cognitive-behavioral intervention for pain, fatigue, and sleep disturbance in cancer Oncology Nursing Forum 2010 37(3)E151--9 Intervention
417.	Pilot randomized controlled trial of a patient-controlled cognitive-behavioral intervention for the pain, fatigue, and sleep disturbance symptom cluster in cancer Journal of pain and symptom management 2012 44(6)810--822 Intervention
418.	Healthy aging - A women's issue Western Journal of Medicine 1997 167(4)220--232 Study design
419.	Followâ€ up protocols for women with cervical cancer after primary treatment Cochrane Database of Systematic Reviews 2013 (11) Study design
420.	Cigarette smoking, physical activity, and alcohol consumption as predictors of cancer incidence among women at high risk of breast cancer in the NSABP P-1 trial Cancer epidemiology, biomarkers {\&} prevention 2014 23(5)823--832 Intervention
421.	A provider intervention to improve colorectal cancer screening in county health centers. Medical care 2008 46(9 Suppl 1)S109--16 Population
422.	A cancer center's approach to engaging African American men about cancer: the men's fellowship breakfast, Southeastern Michigan, 2008-2014. Preventing chronic disease 2014 11()E164 Population
423.	Nutrition, health and disease: A lifespan approach: Second edition 2015 ()1--325 Study design
424.	The polyp prevention trial II: dietary intervention program and participant baseline dietary characteristics. Cancer epidemiology, biomarkers {\&} prevention : a publication of the American Association for Cancer Research, cosponsored by the American Society of Preventive Oncology May 1996 5(5)385--392 Population
425.	Implementation of a 4-y, high-fiber, high-fruit-and-vegetable, low-fat dietary intervention: results of dietary changes in the Polyp Prevention Trial. The American journal of clinical nutrition Sep 2001 74(3)387--401 Population
426.	Storytelling for promoting colorectal cancer screening among underserved Latina women: a randomized pilot study. Cancer control : journal of the Moffitt Cancer Center 2009 16(1)79--87 Population
427.	A cancer screening intervention for underserved Latina women by lay educators. Journal of women's health (2002) May 2012 21(5)557--566 Population
428.	Randomized controlled dissemination study of community-to-clinic navigation to promote CRC screening: Study design and implications. Contemporary clinical trials Feb 2017 53()106--114 Population
429.	Lifestyle characteristics among participants in a Norwegian colorectal cancer screening trial. European journal of cancer prevention : the official journal of the European Cancer Prevention Organisation (ECP) 2006 15(1)10--19 Outcome
430.	Impact of colorectal cancer screening on future lifestyle choices: a three-year randomized controlled trial. Clinical gastroenterology and hepatology : the official clinical practice journal of the American Gastroenterological Association Apr 2007 5(4)477--483 Population
431.	Colorectal cancer screening among ethnically diverse, low-income patients: a randomized controlled trial. Archives of internal medicine May 2011 171(10)906--912 Population
432.	Critical research gaps and recommendations to inform research prioritisation for more effective prevention and improved outcomes in colorectal cancer Gut 2018 Study design
433.	The impact of biological interventions for ulcerative colitis on healthâ€ related quality of life Cochrane Database of Systematic Reviews 2015 (9) Study design
434.	The safety, feasibility, and acceptability of patient-controlled sedation for colonoscopy: prospective study. Hong Kong medical journal = Xianggang yi xue za zhi Apr 2004 10(2)84--88 Intervention
435.	Current clinical applications of omega-6 and omega-3 fatty acids. Nutrition in Clinical Practice Aug 2006 21(4)323--417 Study design
436.	Improving fecal occult blood testing compliance using a mailed educational reminder. Journal of general internal medicine Nov 2009 24(11)1192--1197 Population
437.	Cost-effectiveness of a mailed educational reminder to increase colorectal cancer screening. BMC gastroenterology Aug 2011 11()93 Population
438.	Implementation of supportive care and best supportive care interventions in clinical trials enrolling patients with cancerâ€ . Annals of oncology : official journal of the European Society for Medical Oncology Sep 2015 26(9)1838--1845 Intervention
439.	Positioning and spinal bracing for pain relief in metastatic spinal cord compression in adults Cochrane Database of Systematic Reviews 2015 (9) Study design
440.	Impact of reinforced education by telephone and short message service on the quality of bowel preparation: a randomized controlled study. Endoscopy Nov 2015 47(11)1018--1027 Intervention
441.	Changes in weight and health-related behavior using smartphone applications in patients with colorectal polyps Journal of Nutrition Education and Behavior May 2019 51(5)539--546 Outcome

442.	Study protocol of the CORRECT multicenter trial: the efficacy of blended cognitive behavioral therapy for reducing psychological distress in colorectal cancer survivors. BMC cancer Jul 2018 18(1)748 Study design
443.	Supporting treatment decision making in advanced cancer: a randomized trial of a decision aid for patients with advanced colorectal cancer considering chemotherapy. Journal of clinical oncology : official journal of the American Society of Clinical Oncology May 2011 29(15)2077--2084 Intervention
444.	Colorectal cancer screening and physical activity promotion among obese women: an online evaluation of targeted messages. Journal of health communication 2012 17(10)1187--203 Population
445.	Cluster Randomized Trial of a Church-Based Peer Counselor and Tailored Newsletter Intervention to Promote Colorectal Cancer Screening and Physical Activity Among Older African Americans. Health education {\&} behavior : the official publication of the Society for Public Health Education Population
446.	A randomized, controlled comparison of warm water infusion in lieu of air insufflation versus air insufflation for aiding colonoscopy insertion in sedated patients undergoing colorectal cancer screening and surveillance. Gastrointestinal endoscopy Sep 2009 70(3)505--510 Population
447.	A randomized controlled trial to improve colon cancer screening in rural family medicine: an Iowa Research Network (IRENE) study. Journal of the American Board of Family Medicine : JABFM 2013 26(5)486--497 Population
448.	Continuous local anaesthetic wound infusion for postoperative pain after midline laparotomy for colorectal resection in adults Cochrane Database of Systematic Reviews 2019 (10) Study design
449.	Long-term morbidity and quality of life in patients with localized prostate cancer undergoing definitive radiotherapy or radical prostatectomy International Journal of Radiation Oncology Biology Physics 1999 43(4)735--743 Population
450.	A randomized controlled trial examining the effectiveness of a STOMA psychosocial intervention programme on the outcomes of colorectal patients with a stoma: study protocol. Journal of Advanced Nursing (John Wiley {\&} Sons, Inc.) Jun 2015 71(6)1310--1323 Study design
451.	Pilot trial of a STOMA psychosocial intervention programme for colorectal cancer patients with stomas Journal of advanced nursing 2019 75(6)1338--1346 Intervention
452.	Comparison of the effects of a supervised exercise program and usual care in patients with colorectal cancer undergoing chemotherapy. Cancer nursing 2014 37(2)E21--9 Outcome
453.	Effects of a case management program on patients with oral precancerous lesions: a randomized controlled trial. Supportive care in cancer : official journal of the Multinational Association of Supportive Care in Cancer Jan 2016 24(1)275--284 Population
454.	A multidisciplinary team approach for nutritional interventions conducted by specialist nurses in patients with advanced colorectal cancer undergoing chemotherapy: A clinical trial. Medicine Jun 2017 96(26)e7373 Intervention
455.	Food and Western Disease: Health and Nutrition from an Evolutionary Perspective 2009 ()1--354 Study design
456.	Worry associated with screening for colorectal carcinomas. Scandinavian journal of gastroenterology Mar 1997 32(3)238--245 Population
457.	Physicians encouraging colorectal screening: a randomized controlled trial of enhanced office and patient management on compliance with colorectal cancer screening. Archives of internal medicine Jan 2009 169(1)47--55 Population
458.	Modifying attributions of colorectal cancer risk Cancer Epidemiology Biomarkers {\&} Prevention 2004 13(4)560--566 Intervention
459.	Effects of communicating social comparison information on risk perceptions for colorectal cancer. Journal of health communication Jun 2006 11(4)391--407 Population
460.	Cancer Prevention Research: Back to the future Cancer Prevention Research 2009 2(6)503--513 Study design
461.	AACR White paper: Shaping the future of cancer prevention - A roadmap for advancing science and public health Cancer Prevention Research 2018 11(12)735--778 Study design
462.	Herbal preparations for uterine fibroids Cochrane Database of Systematic Reviews 2013 (4) Study design
463.	Herbal medicines for fatty liver diseases Cochrane Database of Systematic Reviews 2013 (8) Study design
464.	High dose rate versus low dose rate intracavity brachytherapy for locally advanced uterine cervix cancer Cochrane Database of Systematic Reviews 2014 (10) Study design
465.	How acceptable is a referral and telephone-based outcall programme for men diagnosed with cancer? A feasibility study. European journal of cancer care Dec 2006 15(5)467--475 Outcome
466.	The psychological impact of a specialist referral and telephone intervention on male cancer patients: a randomised controlled trial. Psycho-oncology Jun 2010 19(6)617--625 Outcome
467.	Storytelling for promoting colorectal cancer prevention and early detection among Latinos. Patient Education {\&} Counseling Aug 2007 67(3)272--278 Intervention

468. Mesh versus nonâ€ mesh for inguinal and femoral hernia repair Cochrane Database of Systematic Reviews 2018 (9) Population
469. Integrative Medicine for Children 2009 Population
470. Exercise interventions for people undergoing multimodal cancer treatment that includes surgery Cochrane Database of Systematic Reviews 2018 (12) Study design
471. The Family Health Promotion Project (FHPP): design and baseline data from a randomized trial to increase colonoscopy screening in high risk families. Contemporary clinical trials Mar 2012 33(2)426--435 Population
472. Perioperative enhanced recovery programmes for gynaecological cancer patients Cochrane Database of Systematic Reviews 2015 (3) Study design
473. Facilitating psychosexual adjustment for women undergoing pelvic radiotherapy: pilot of a novel patient psycho-educational resource. Health expectations : an international journal of public participation in health care and health policy Dec 2016 19(6)1290--1301 Outcome
474. The Impact of Total Body Prehabilitation on Post-Operative Outcomes After Major Abdominal Surgery: A Systematic Review World Journal of Surgery 2018 42(9)2781--2791 Study design
475. New issues in genetic counseling of hereditary colon cancer. Clinical cancer research : an official journal of the American Association for Cancer Research Nov 2007 13(22)6857s--61s Outcome
476. Determinants of successful implementation of population-based cancer screening programmes European Journal of Cancer 2012 48(5)743--748 Study design
477. Radioiodine therapy for differentiated thyroid carcinoma with thyroglobulin positive and radioactive iodine negative metastases Cochrane Database of Systematic Reviews 2009 (1) Study design
478. Interventions for anal canal intraepithelial neoplasia Cochrane Database of Systematic Reviews 2012 (12) Intervention
479. Bisphosphonates for advanced prostate cancer Cochrane Database of Systematic Reviews 2017 (12) Study design
480. A multimedia patient education program on colorectal cancer screening increases knowledge and willingness to consider screening among Hispanic/Latino patients. Patient education and counseling Aug 2009 76(2)220--226 Population
481. Dietary and serum alpha-tocopherol, beta-carotene and retinol, and risk for colorectal cancer in male smokers. European journal of clinical nutrition Jul 2002 56(7)615--621 Population
482. Postoperative radiotherapy for prostate cancer: a comparison of four consensus guidelines and dosimetric evaluation of 3D-CRT versus tomotherapy IMRT. International journal of radiation oncology, biology, physics Nov 2012 84(3)725--732 Study design
483. Folic acid with or without vitamin B12 for the prevention and treatment of healthy elderly and demented people Cochrane Database of Systematic Reviews 2008 (4) Outcome
484. A randomized trial of generic versus tailored interventions to increase colorectal cancer screening among intermediate risk siblings Annals of Behavioral Medicine Apr 2009 37(2)207--217 Population
485. The VITamin D and OmegA-3 TriaL (VITAL): Rationale and design of a large randomized controlled trial of vitamin D and marine omega-3 fatty acid supplements for the primary prevention of cancer and cardiovascular disease Contemporary Clinical Trials 2012 33(1)159--171 Study design
486. The efficacy of tailored print materials in promoting colorectal cancer screening: results from a randomized trial involving callers to the National Cancer Institute's Cancer Information Service. Journal of health communication 2005 10()83--104 Population
487. Physical activity intervention studies - What we know and what we need to know - A scientific statement from the American Heart Association Council on Nutrition, Physical Activity, and Metabolism (Subcommittee on Physical Activity); Council on Cardiovascu Circulation 2006 Study design
488. Nutrition and human health from a sex-gender perspective Molecular Aspects of Medicine 2011 32(1)1--70 Study design
489. Nutrition and human health from a sex-gender perspective Molecular Aspects of Medicine 2011 32(1)1--70 Outcome
490. Longâ€ term hormone therapy for perimenopausal and postmenopausal women Cochrane Database of Systematic Reviews 2017 (1) Study design
491. Pharmacological interventions for treating heart failure in patients with Chagas cardiomyopathy Cochrane Database of Systematic Reviews 2016 (7) Study design
492. Primary prevention of colorectal cancer: lifestyle, nutrition, exercise. Recent results in cancer research. Fortschritte der Krebsforschung. Progres dans les recherches sur le cancer 2005 166()177--211 Outcome
493. Effect of a Nurse-Led Psychoeducational Intervention on Healthcare Service Utilization Among Adults With Advanced Cancer. Oncology nursing forum Jul 2015 42(4)E310--8 Outcome
494. A mobile device application (app) to improve adherence to an enhanced recovery program for colorectal surgery: a randomized controlled trial Surgical Endoscopy 2020 34(2)742--751 Outcome

495. Randomized phase II study of S-1 dosing schedule for resected colorectal cancer. BMC cancer Jun 2015 15()452 Outcome
496. Cognitive Behavioral Therapy for Insomnia Outcomes in Women After Primary Breast Cancer Treatment: A Randomized, Controlled Trial Oncology Nursing Forum 2014 41(3)241--253 Intervention
497. Results of a community-based randomized trial to increase colorectal cancer screening among Filipino Americans. American journal of public health Nov 2010 100(11)2228--2234 Population
498. Wearable activity technology and action-planning (WATAAP) to promote physical activity in cancer survivors: Randomised controlled trial protocol International Journal of Clinical and Health Psychology May 2018 18(2)124--132 Study design
499. Mobilizing social support networks to improve cancer screening: the COACH randomized controlled trial study design. BMC Cancer Jan 2015 15(1)907 Population
500. Enhancing adherence in trials promoting change in diet and physical activity in individuals with a diagnosis of colorectal adenoma; a systematic review of behavioural intervention approaches. BMC cancer Jul 2015 15()505 Study design
501. Differences in response to a dietary intervention between the general population and first-degree relatives of colorectal cancer patients. Journal of nutrition education and behavior 2014 46(5)376--383 Outcome
502. Physical activity interventions for diseaseâ€ related physical and mental health during and following treatment in people with nonâ€ advanced colorectal cancer Cochrane Database of Systematic Reviews 2020 (5) Study design
503. Colon cancer information as a source of exercise motivation for relatives of patients with colon cancer. Psychology, health {\&} medicine Dec 2010 15(6)729--741 Intervention
504. The effect of a multifaceted efficacy intervention on exercise behavior in relatives of colon cancer patients. International journal of behavioral medicine Dec 2012 19(4)550--562 Population
505. The impact on health-related quality of life in the first 12 months: a randomised comparison of preoperative short-course radiation versus long-course chemoradiation for T3 rectal cancer (Trans-Tasman Radiation Oncology Group Trial 01.04) European journal of cancer (oxford, england : 1990) 2016 55()15--26 Outcome
506. Outsmart HPV: Acceptability and short-term effects of a web-based HPV vaccination intervention for young adult gay and bisexual men. Vaccine Dec 2018 36(52)8158--8164 Population
507. Exercise clinical trials in cancer prevention research: A call to action Cancer Epidemiology Biomarkers and Prevention 1999 8(3)201--207 Study design
508. Educating patients with limited literacy skills: the effectiveness of printed and videotaped materials about colon cancer. American journal of public health Jan 1994 84(1)119--121 Intervention
509. American association of clinical endocrinologists medical guidelines for the clinical use of dietary supplements and nutraceuticals Endocrine Practice 2003 9(5)417--470 Study design
510. Laparoscopy versus laparotomy for benign ovarian tumour Cochrane Database of Systematic Reviews 2009 (2) Study design
511. Ablation for atrial fibrillation: an evidence-based analysis Ontario health technology assessment series 2006 6(7)1--63 Study design
512. Balloon kyphoplasty for treatment of painful osteoporotic vertebral compression fractures: an evidence update Ontario health technology assessment series 2010 10(20)1--22 Outcome
513. Primary prevention of diseases of old age Clinics in Geriatric Medicine 2002 18(3)407--+ Population
514. Interactive, culturally sensitive education on colorectal cancer screening. Medical care Sep 2008 46(9)S44--50 Population
515. A randomized trial comparing the effect of two phone-based interventions on colorectal cancer screening adherence. Annals of behavioral medicine : a publication of the Society of Behavioral Medicine Dec 2011 42(3)294--303 Population
516. Adherence with postdischarge venous thromboembolism chemoprophylaxis recommendations after colorectal cancer surgery among elderly Medicare beneficiaries. Annals of surgery Jul 2014 260(1)103--108 Outcome
517. Randomized Trial of a Web-Based Intervention to Address Barriers to Clinical Trials. Journal of clinical oncology : official journal of the American Society of Clinical Oncology Feb 2016 34(5)469--478 Intervention
518. Cancer Prevention: Obstacles, Challenges, and the Road Ahead Journal of the National Cancer Institute 2016 108(2) Study design
519. Adjuvant therapy with antidepressants for the management of inflammatory bowel disease Cochrane Database of Systematic Reviews 2019 (4) Study design
520. Vaginal dilator therapy for women receiving pelvic radiotherapy Cochrane Database of Systematic Reviews 2014 (9) Study design
521. Concentrated sugars and incidence of prostate cancer in a prospective cohort British Journal of Nutrition 2018 120(6)703--710 Population

522. Using a computer to teach patients about fecal occult blood screening. A randomized trial. Journal of general internal medicine Nov 2005 20(11)984--988 Population
523. Multimodal prehabilitation improves functional capacity before and after colorectal surgery for cancer: a five-year research experience. Acta oncologica (Stockholm, Sweden) Feb 2017 56(2)295--300 Outcome
524. Role and impact of multimodal prehabilitation for gynecologic oncology patients in an Enhanced Recovery After Surgery (ERAS) program International Journal of Gynecological Cancer 2019 29(8)1235--1243 Outcome
525. Exercise interventions on health-related quality of life for cancer survivors. The Cochrane database of systematic reviews Aug 2012 (8)CD007566 Study design
526. Exercise interventions on healthâ€ related quality of life for people with cancer during active treatment Cochrane Database of Systematic Reviews 2012 (8) Study design
527. Cost effectiveness of interventions to promote screening for colorectal cancer: a randomized trial. Journal of preventive medicine and public health = Yebang Uihakhoe chi May 2011 44(3)101--110 Outcome
528. Fish oil decreases C-reactive protein/albumin ratio improving nutritional prognosis and plasma fatty acid profile in colorectal cancer patients. Lipids Sep 2013 48(9)879--888 Outcome
529. Riskâ€ reducing medications for primary breast cancer: a network metaâ€ analysis Cochrane Database of Systematic Reviews 2019 (4) Study design
530. Alphaâ€ glucosidase inhibitors for prevention or delay of type 2 diabetes mellitus and its associated complications in people at increased risk of developing type 2 diabetes mellitus Cochrane Database of Systematic Reviews 2018 (12) Population
531. Fruits 2020 10(12) Study design
532. Sedentary behavior is associated with colorectal adenoma recurrence in men Cancer Causes {\&} Control Oct 2014 25(10)1387--1395 Population
533. Guidelines for the management of hereditary colorectal cancer from the British Society of Gastroenterology (BSG)/Association of Coloproctology of Great Britain and Ireland (ACPGBI)/United Kingdom Cancer Genetics Group (UKCGG) Gut 2020 69(3)411--444 Study design
534. Vitamin D and Physical Activity in Patients With Colorectal Cancer: Epidemiological Evidence and Therapeutic Implications. Cancer Journal (Sudbury, Mass.) May 2016 22(3)223--231 Outcome
535. Effects of home-based diet and exercise on functional outcomes among older, overweight long-term cancer survivors: RENEW: a randomized controlled trial. JAMA May 2009 301(18)1883--91 Study design
536. Group trajectory analysis helps to identify older cancer survivors who benefit from distance-based lifestyle interventions. Cancer 2015 121(24)4433--4440 Outcome
537. Culturally targeted educational intervention to increase colorectal health awareness among African Americans. Journal of health care for the poor and underserved Aug 2010 21(3)132--147 Population
538. Exercise during and after neoadjuvant rectal cancer treatment (the EXERT trial): study protocol for a randomized controlled trial. Trials Jan 2018 19(1)35 Study design
539. Lifestyle interventions are feasible in patients with colorectal cancer with potential short-term health benefits: a systematic review. International journal of colorectal disease 2017 32(6)765--775 Study design
540. Interventions outside the workplace for reducing sedentary behaviour in adults under 60 years of age Cochrane Database of Systematic Reviews 2020 (7) Intervention
541. Blood pressure lowering efficacy of renin inhibitors for primary hypertension Cochrane Database of Systematic Reviews 2017 (4) Study design
542. A randomized controlled trial of the impact of targeted and tailored interventions on colorectal cancer screening. Cancer Nov 2007 110(9)2083--2091 Population
543. A randomized trial of genetic and environmental risk assessment (GERA) for colorectal cancer risk in primary care: trial design and baseline findings. Contemporary clinical trials Jan 2011 32(1)25--31 Outcome
544. A randomized controlled trial of a tailored navigation and a standard intervention in colorectal cancer screening. Cancer epidemiology, biomarkers {\&} prevention : a publication of the American Association for Cancer Research, cosponsored by the American Society of Preventive Oncology Jan 2013 22(1)109--117 Population
545. Geriatric diseases: Evaluation and management 2018 ()1--834 Study design
546. Effect of listening to music and essential oil inhalation on patients undergoing screening CT colonography: a randomized controlled trial. European journal of radiology Dec 2014 83(12)2172--2176 Population
547. Type II or type III radical hysterectomy compared to chemoradiotherapy as a primary intervention for stage IB2 cervical cancer Cochrane Database of Systematic Reviews 2018 (10) Study design
548. Bone Health in Gynecologic Cancers-does FOSAVANCE Help? Https://clinicaltrials.gov/show/nct00593580 Outcome

549. Women's Health Initiative (WHI) https://clinicaltrials.gov/show/NCT00000611 1999 Population
550. Combination Herbal Therapy (CHT) Versus Placebo in Patients With Irritable Bowel Syndrome (IBS) https://clinicaltrials.gov/show/NCT00130741 2005 Outcome
551. Rehabilitation of Cancer Survivors in Denmark: the Effect of a Psychosocial Rehabilitation Course https://clinicaltrials.gov/show/NCT01086683 2010 Intervention
552. The Efficacy of Health Partnership Program for Cancer Patients https://clinicaltrials.gov/show/NCT01527409 2012 Outcome
553. Early Rehabilitation of Cancer Patients https://clinicaltrials.gov/show/NCT01588262 2012 13()9 Outcome
554. Randomized Study Comparing the Use of Epidural Analgesia to Intravenous Narcotics for Laparoscopic Colorectal Resection https://clinicaltrials.gov/show/NCT02086123 2013 Outcome
555. Physical Training and Cancer-a Multicenter Clinical Trial https://clinicaltrials.gov/show/NCT02473003 2015 Outcome
556. Men Together Making a Difference: health Promotion for Black Men https://clinicaltrials.gov/show/NCT02572414 2015 Population
557. Efficacy of a Smart Management Strategy for Health (SMASH) Program for Overcoming Cancer Crisis and Growing Positively https://clinicaltrials.gov/show/NCT02650661 2015 Outcome
558. Physical Activity Intervention With People at Increased Risk of Developing Colon Cancer https://clinicaltrials.gov/show/NCT02724306 2016 Population
559. Enhancing Prevention Pathways Toward Tribal Colorectal Health https://clinicaltrials.gov/show/NCT02882620 2016 Outcome
560. STI.VI. Study: how to Improve Lifestyles in Screening Contexts https://clinicaltrials.gov/show/NCT03118882 2017 Population
561. Views on Cancer Prevention and Daily Lifestyle https://clinicaltrials.gov/show/NCT03575117 2018 Population
562. Exploring the Effects of a High Chlorophyll Dietary Intervention to Reduce Colon Cancer Risk in Adults https://clinicaltrials.gov/show/NCT03582306 2018 Population
563. Internet-Based Motivational Interviewing for Colonoscopy https://clinicaltrials.gov/show/NCT03595904 2018 Population
564. Randomized Trial : hemorrhoidal Pedicle Ligation vs Laser vs Open Hemorrhoidectomy https://clinicaltrials.gov/show/NCT03757728 2018 Population
565. Adapting Multiple Behavior Interventions That Effectively Improve Cancer Survivor Health Cancer Survivor Health https://clinicaltrials.gov/show/NCT04000880 1
566. The Diagnosis of Invasive Depth of Early Rectal Cancer https://clinicaltrials.gov/show/NCT04222530 2020 Intervention
567. Lifestyle Intervention Among Participants of the French Colorectal Cancer Screening Program https://clinicaltrials.gov/show/NCT04257526 2020 Population
568. The Impact of Physical Exercise on Sleep in Colorectal Cancer Patients During Prehabilitation Period https://clinicaltrials.gov/show/NCT04270500 2020 Outcome
569. Reducing Fear of Cancer Recurrence in Danish Colorectal Cancer Survivors https://clinicaltrials.gov/show/NCT04287218 2020 Population
570. Longitudinal Incision Versus Cruciate Incision in the Construction of an End Colostomy https://clinicaltrials.gov/show/NCT04397783 2020 Population
571. Vitamin D-induced molecular mechanisms to potentiate cancer therapy and to reverse drug-resistance in cancer cells Nutrients 2020 12(6)1--25 Outcome
572. Natalizumab for induction of remission in Crohn's disease Cochrane Database of Systematic Reviews 2018 (8) Study design
573. From the bench to public health: Population-level implementation intentions in colorectal cancer screening American Journal of Preventive Medicine 2014 46(3)273--280 Population
574. The influence of health and lifestyle characteristics on the relation of serum 25-hydroxyvitamin D with risk of colorectal and breast cancer in postmenopausal women American Journal of Epidemiology 2012 Intervention
575. Lamotrigine versus carbamazepine monotherapy for epilepsy: an individual participant data review Cochrane Database of Systematic Reviews 2018 (6) Study design
576. Effectiveness of Lay Health Worker Outreach in Reducing Disparities in Colorectal Cancer Screening in Vietnamese Americans. American journal of public health 2015 105(10)2083--2089 Population
577. Treatment and prevention of pouchitis after ileal pouchâ€ anal anastomosis for chronic ulcerative colitis Cochrane Database of Systematic Reviews 2019 (11) Study design
578. Blood CEA levels for detecting recurrent colorectal cancer Cochrane Database of Systematic Reviews 2015 (12) Study design
579. Imaging modalities for the nonâ€ invasive diagnosis of endometriosis Cochrane Database of Systematic Reviews 2016 (2) Study design

580. Combination of the nonâ€ invasive tests for the diagnosis of endometriosis Cochrane Database of Systematic Reviews 2016 (7) Study design
581. Rouxâ€ enâ€ Y versus Billrothâ€ I reconstruction after distal gastrectomy for gastric cancer Cochrane Database of Systematic Reviews 2018 (4) Study design
582. Physical training and cancer (phys-can)-design and description of a multicenter clinical trial within a multidisciplinary consortium Psycho-oncology. 2014 23()205 Outcome
583. Negative pressure wound therapy for surgical wounds healing by primary closure Cochrane Database of Systematic Reviews 2020 (6) Study design
584. Feasibility of a novel exercise prehabilitation programme in patients scheduled for elective colorectal surgery: a feasibility randomised controlled trial Supportive care in cancer 2020 28(7)3197--3206 Outcome
585. A new model of early, integrated palliative care: palliative rehabilitation for newly diagnosed patients with non-resectable cancer. Supportive care in cancer : official journal of the Multinational Association of Supportive Care in Cancer Sep 2019 27(9)3291--3300 Study design
586. Evaluation of intervention strategies to manage fatigue during active treatment and to prevent persistent fatigue after curative treatment for cancer http://www.who.int/trialsearch/Trial2.aspx?TrialID=NTR183 2005 () Study design
587. Anticipated regret to increase uptake of colorectal cancer screening in Scotland (ARTICS): study protocol for a randomised controlled trial. BMC public health Sep 2013 13()849 Population
588. Randomised controlled trial of a tailored information pack for patients undergoing surgery and treatment for rectal cancer. European journal of oncology nursing : the official journal of European Oncology Nursing Society Apr 2014 18(2)183--191 Outcome
589. Development and implementation of a medical oncology quality improvement tool for a regional community oncology network: the Fox Chase Cancer Center Partners initiative. Journal of the National Comprehensive Cancer Network : JNCCN Oct 2007 5(9)875--882 Intervention
590. Effects of a psycho-educational programme on health-related quality of life in patients treated for colorectal and anal cancer: A feasibility trial. European journal of oncology nursing : the official journal of European Oncology Nursing Society Apr 2016 21()181--188 Outcome
591. Assessing the information desire of patients with advanced cancer by providing information with a decision aid, which is evaluated in a randomized trial: a study protocol. BMC medical informatics and decision making Feb 2011 11()9 Intervention
592. Effect of an Educational Video to Increase Calls and Screening into an Anal Cancer Clinical Trial Among HIV+ Hispanics in PR: Results from a Randomized Controlled Behavioral Trial. AIDS and behavior May 2019 23(5)1135--1146 Population
593. Epidural analgesia combined with a comprehensive physiotherapy program after Cytoreductive Surgery and HIPEC is associated with enhanced post-operative recovery and reduces intensive care unit stay: A retrospective study of 124 patients Ejso 2016 42(12)1938--1943 Intervention
594. Quality of life after rectal resection for cancer, with or without permanent colostomy. Cochrane Database of Systematic Reviews 2012 (12) Study design
595. Living With Cancer: Assessing The Educational, Symptomatic, and Quality Of Life Impact Of a Comprehensive Patient Symposia On Cancer Patients Blood 2013 122(21) Study design
596. A Web-based Multimedia Program Before Colonoscopy Increased Knowledge and Decreased Anxiety, Sedation Requirement, and Procedure Time. Journal of clinical gastroenterology Jul 2018 52(6)519--523 Population
597. The recruitment of African-Americans to cancer prevention and control studies Preventive Medicine 1996 25(5)547--553 Study design
598. Systemic treatments for metastatic cutaneous melanoma Cochrane Database of Systematic Reviews 2018 (2) Study design
599. Closure methods for laparotomy incisions for preventing incisional hernias and other wound complications Cochrane Database of Systematic Reviews 2017 (11) Study design
600. Whole brain radiation therapy (WBRT) alone versus WBRT and radiosurgery for the treatment of brain metastases Cochrane Database of Systematic Reviews 2017 (9) Study design
601. Screening for carcinoma of the prostate by digital rectal examination in a randomly selected population BMJ Population
602. Long-term health outcomes and cost-effectiveness of a computer-tailored physical activity intervention among people aged over fifty: modelling the results of a randomized controlled trial. BMC public health Oct 2014 14()1099 Outcome
603. Physical Activity Maintenance Following Home-Based, Individually Tailored Print Interventions for African American Women Health Promotion Practice 2020 21(2)268--276 Population
604. Spinal cord stimulation for cancerâ€ related pain in adults Cochrane Database of Systematic Reviews 2015 (6) Outcome
605. Effectiveness of probiotics, prebiotics, and prebiotic-like components in common functional foods Comprehensive Reviews in Food Science and Food Safety 2020 19(4)1908--1933 Study design

606. A culturally tailored navigator program for colorectal cancer screening in a community health center: a randomized, controlled trial. Journal of general internal medicine Feb 2009 24(2)211--217 Population
607. Older Adults with Cancer: A Randomized Controlled Trial of Occupational and Physical Therapy. Journal of the American Geriatrics Society May 2019 67(5)953--960 Intervention
608. Uncovering the Genetic Architecture of Colorectal Cancer with Focus of Rare and Less Frequent Variants Database of Genotypes and Phenotypes: dbGaP 2017 Outcome
609. Health4Families: an intervention to improve weight and health behaviors in families with hereditary breast/ovarian cancer or Lynch syndrome Journal of clinical oncology 2017 35(15) Population
610. The ASAMET trial: a randomized, phase II, double-blind, placebo-controlled, multicenter, 2â€‰Ã—â€‰2 factorial biomarker study of tertiary prevention with low-dose aspirin and metformin in stage I-III colorectal cancer patients BMC cancer 2018 18(1)1210 Outcome
611. Videotape-based decision aid for colon cancer screening. A randomized, controlled trial. Annals of internal medicine Nov 2000 133(10)761--769 Population
612. Enrollment of racial and ethnic minorities in the Prostate, Lung, Colorectal and Ovarian Cancer Screening Trial. Journal of the National Medical Association 2008 100(3)291--298 Population
613. Cancer screening utilization: is there a role for social work in cancer prevention? Social work in health care 1995 21(4)59--70 Population
614. Improving medical students' communication with limited-literacy patients: project development and implementation. Journal of cancer education : the official journal of the American Association for Cancer Education 2001 16(2)68--71 Population
615. The tired study: two interventions for fatigued advanced cancer patients, a randomised controlled trial Psycho-oncology 2013 22()231-- Intervention
616. Cognitive behavioral therapy or graded exercise therapy compared with usual care for severe fatigue in patients with advanced cancer during treatment: a randomized controlled trial Annals of oncology 2020 31(1)115--122 Intervention
617. Objectively measured mobilisation is enhanced by a new behaviour support tool in patients undergoing abdominal cancer surgery European Journl of Surgical Oncology Intervention
618. Determinants of colorectal cancer screening in primary care. Journal of cancer education : the official journal of the American Association for Cancer Education 2008 23(4)241--247 Population
619. Offering annual fecal occult blood tests at annual flu shot clinics increases colorectal cancer screening rates. Annals of family medicine 2009 7(1)17--23 Population
620. Comparative effectiveness of two pharmacy-based colorectal cancer screening interventions during an annual influenza vaccination campaign. Journal of the American Pharmacists Association : JAPhA 2010 50(2)181--187 Population
621. An intervention to decrease cancer fatalism among rural elders. Oncology nursing forum Apr 1999 26(3)583--588 Intervention
622. An intervention study to increase colorectal cancer knowledge and screening among community elders. Public health nursing (Boston, Mass.) 2004 21(5)435--442 Population
623. Evaluation of efficacy and efficiency of a pragmatic intervention by a social worker to support informal caregivers of elderly patients (The ICE Study): study protocol for a randomized controlled trial Trials 2016 17(1)531 Study design
624. Nonprescription product therapeutics: Second edition 2011 ()1--887 Study design
625. Low-Fat Dietary Pattern among Postmenopausal Women Influences Long-Term Cancer, Cardiovascular Disease, and Diabetes Outcomes. The Journal of nutrition Sep 2019 149(9)1565--1574 Population
626. The therapeutic importance of acid-base balance Biochemical Pharmacology 2021 183 Study design
627. The effect of a school-based educational intervention on gender differences in reported family cancer history. Journal of cancer education : the official journal of the American Association for Cancer Education 2008 23(3)180--185 Intervention
628. DOSE ESCALATION FOR METASTATIC SPINAL CORD COMPRESSION IN PATIENTS WITH RELATIVELY RADIORESISTANT TUMORS International Journal of Radiation Oncology Biology Physics 2011 80(5)1492--1497 Outcome
629. Systemic antibiotics for treating malignant wounds Cochrane Database of Systematic Reviews 2017 (8) Study design
630. Assessing the effect of patient navigator assistance for psychosocial support services on health-related quality of life in a randomized clinical trial in Latino breast, prostate, and colorectal cancer survivors Cancer 2020 126(5)1112--1123 Intervention
631. Prevention of colorectal cancer: The future is now Current Colorectal Cancer Reports 2014 10(1)84--93 Study design
632. Laparoscopic versus open liver resection for benign and malignant hepatic lesions in adults Cochrane Database of Systematic Reviews 2013 (5) Study design

633. Individualized nutrition intervention is of major benefit to colorectal cancer patients: long-term follow-up of a randomized controlled trial of nutritional therapy American Journal of Clinical Nutrition 2012 96(6)1346--1353 Outcome
634. Intervention to improve psychological functioning for newly diagnosed patients with cancer. Oncology nursing forum Jul 2002 29(6)967--975 Intervention
635. A randomized trial of two print interventions to increase colon cancer screening among first-degree relatives. Patient education and counseling May 2008 71(2)215--227 Population
636. Telephone interventions for symptom management in adults with cancer Cochrane Database of Systematic Reviews 2020 (6) Study design
637. Treatment for Barrett's oesophagus Cochrane Database of Systematic Reviews 2010 (1) Study design
638. Thalidomide for managing cancer cachexia Cochrane Database of Systematic Reviews 2012 (4) Outcome
639. Communication preference moderates the effect of a tailored intervention to increase colorectal cancer screening among African Americans. Patient education and counseling Dec 2014 97(3)370--375 Population
640. Neuromodulators for pain management in rheumatoid arthritis Cochrane Database of Systematic Reviews 2012 (1) Study design
641. Knowledge, ignorance and priorities for research in key areas of cancer survivorship: findings from a scoping review British Journal of Cancer 2011 105()S82--S94 Study design
642. Pioglitazone for type 2 diabetes mellitus Cochrane Database of Systematic Reviews 2006 (4) Population
643. Interventions for preventing oral mucositis in patients with cancer receiving treatment: oral cryotherapy Cochrane Database of Systematic Reviews 2015 (12) Population
644. CO2 insufflation during colonoscopy decreases post-interventional pain in deeply sedated patients: a randomized controlled trial. Wiener klinische Wochenschrift Population
645. Personal navigation increases colorectal cancer screening uptake. Cancer epidemiology, biomarkers {\&} prevention : a publication of the American Association for Cancer Research, cosponsored by the American Society of Preventive Oncology Mar 2015 24(3)506--511 Population
646. Taxonomy for colorectal cancer screening promotion: Lessons from recent randomized controlled trials. Preventive medicine Aug 2017 101()229--234 Population
647. [Bowel preparation for colonoscopy. Any significant progress on the horizon?]. Gastroenterologia y hepatologia Apr 2015 38(4)287--300 Population
648. Laparoscopic versus open distal pancreatectomy for pancreatic cancer Cochrane Database of Systematic Reviews 2016 (4) Population
649. Can cancer risk information raise awareness without increasing anxiety? A randomized trial. Preventive medicine Sep 2006 43(3)187--190 Intervention
650. The impact of individually-tailored lifestyle advice in the colorectal cancer screening context: A randomised pilot study in North-West London Preventive Medicine 2010 Outcome
651. Dose-Dependent Increases in Ellagitannin Metabolites as Biomarkers of Intake in Humans Consuming Standardized Black Raspberry Food Products Designed for Clinical Trials Molecular Nutrition and Food Research 2020 64(10) Outcome
652. Effectiveness, training and quality assurance of colonoscopy screening for colorectal cancer. Gut Jun 2015 64(6)982--990 Population
653. Effect of testing for cancer on cancerâ€ or venous thromboembolism (VTE)â€ related mortality and morbidity in people with unprovoked VTE Cochrane Database of Systematic Reviews 2018 (11) Study design
654. Health disadvantages in colorectal cancer screening among African Americans: considering the cultural context of narrative health promotion. Journal of Health Care for the Poor {\&} Underserved May 2009 20(2)102--119 Population
655. Assessment is not enough: a randomized controlled trial of the effects of HRQL assessment on quality of life and satisfaction in oncology clinical practice. Psycho-oncology Dec 2007 16(12)1069--1079 Outcome
656. In a randomized controlled trial, missing data led to biased results regarding anxiety Journal of clinical epidemiology 2004 57(11)1131--1137 Outcome
657. A randomized psychosocial intervention study on the effect of home visits on the well-being of Danish colorectal cancer patients--the INCA Project. Psycho-oncology Nov 2005 14(11)949--961 Intervention
658. No effect on survival of home psychosocial intervention in a randomized study of Danish colorectal cancer patients. Psycho-oncology Aug 2009 18(8)875--885 Intervention
659. Modern epidemiology: Third edition 2011 ()1--758 Study design
660. Computed tomography for diagnosis of acute appendicitis in adults Cochrane Database of Systematic Reviews 2019 (11) Study design

661. Nonâ€ invasive interventions for improving wellâ€ being and quality of life in patients with lung cancer Cochrane Database of Systematic Reviews 2011 (9) Study design
662. Hormone replacement therapy after surgery for epithelial ovarian cancer Cochrane Database of Systematic Reviews 2020 (1) Study design
663. Epidural analgesia versus patientâ€ controlled intravenous analgesia for pain following intraâ€ abdominal surgery in adults Cochrane Database of Systematic Reviews 2018 (8) Study design
664. Effectiveness of a theory-based intervention to increase colorectal cancer screening among Iranian health club members: a randomized trial. Journal of behavioral medicine Oct 2014 37(5)1019--1029 Population
665. Motivational interviewing and screening colonoscopy in high-risk individuals. A randomized controlled trial. Patient education and counseling Jun 2018 101(6)1082--1087 Intervention
666. Nutritional countermeasures targeting reactive oxygen species in cancer: From mechanisms to biomarkers and clinical evidence Antioxidants and Redox Signaling 2013 19(17)2157--2196 Outcome
667. Post-study aspirin intake and factors motivating participation in a colorectal cancer chemoprevention trial. Cancer epidemiology, biomarkers {\&} prevention : a publication of the American Association for Cancer Research, cosponsored by the American Society of Preventive Oncology Mar 2002 11(3)281--285 Outcome
668. Randomized Evaluation of Cognitive-Behavioral Therapy and Graded Exercise Therapy for Post-Cancer Fatigue. Journal of pain and symptom management 2017 54(1)74--84 Outcome
669. Adoption, reach, and implementation of a cancer education intervention in African American churches. Implementation Science Mar 2017 12()1--11 Intervention
670. Peer support interventions for parents and carers of children with complex needs Cochrane Database of Systematic Reviews 2013 (6) Study design
671. Holmium laser enucleation of the prostate for the treatment of lower urinary tract symptoms in men with benign prostatic hyperplasia Cochrane Database of Systematic Reviews 2019 (3) Study design
672. Longitudinal changes in lifestyle behaviors and health status in colon cancer survivors. Cancer epidemiology, biomarkers {\&} prevention : a publication of the American Association for Cancer Research, cosponsored by the American Society of Preventive Oncology Jun 2004 13(6)1022--1031 Outcome
673. A multi-disciplinary model of survivorship care following definitive chemoradiation for anal cancer BMC Cancer 2019 19(1) Study design
674. Full publication of results initially presented in abstracts Cochrane Database of Systematic Reviews 2018 (11) Study design
675. Komen/ASCO program aims to swell ranks of minority oncologists. Feb 2009 101(4)224--225,227 Study design
676. Patient Empowerment Improved Perioperative Quality of Care in Cancer Patients Aged \textgreater= 65 Years A Randomized Controlled Trial Plos One 2015 10(9) Population
677. Buprenorphine for treating cancer pain Cochrane Database of Systematic Reviews 2015 (3) Outcome
678. Oxycodone for cancerâ€ related pain Cochrane Database of Systematic Reviews 2017 (8) Study design
679. Systematic screening and assessment of psychosocial wellâ€ being and care needs of people with cancer Cochrane Database of Systematic Reviews 2019 (3) Study design
680. Postoperative complications and mobilisation following major abdominal surgery with vs. Without fitness tracker-based feedback (EXPELLIARMUS): Study protocol for a student-led multicentre randomised controlled trial (CHIR-Net SIGMA study group) Trials Study design
681. Fast-track" rehabilitation for elective colonic surgery in Germany - prospective observational data from a multi-centre quality assurance programme" International Journal of Colorectal Disease 2008 23(1)93--99 Outcome
682. The Role of Aspirin, Vitamin D, Exercise, Diet, Statins, and Metformin in the Prevention and Treatment of Colorectal Cancer. Current treatment options in oncology 2015 16(9)43 Outcome
683. Effectiveness of a brief phone intervention to increase participation in a population-based colorectal cancer screening programme: a randomized controlled trial. Colorectal disease : the official journal of the Association of Coloproctology of Great Britain and Ireland Oct 2019 21(10)1120--1129 Population
684. Screening for colorectal cancer by once only sigmoidoscopy: a feasibility study in Turin, Italy. Journal of medical screening 1996 3(2)72--78 Population
685. Patient and physician reminders to promote colorectal cancer screening: a randomized controlled trial. Archives of internal medicine Feb 2009 169(4)364--371 Population
686. Nature-derived compounds modulating Wnt/β-catenin pathway: a preventive and therapeutic opportunity in neoplastic diseases Acta Pharmaceutica Sinica B 2020 10(10)1814--1834 Outcome
687. Colorectal cancer video for the deaf community: a randomized control trial. Journal of cancer education : the official journal of the American Association for Cancer Education Dec 2010 25(4)518--523 Intervention

688.	Effect of an educational pamphlet on colon cancer screening: a randomized, prospective trial. European journal of gastroenterology {\&} hepatology Apr 2010 22(4)444--449 Population
689.	Costs and cost effectiveness of a health care provider-directed intervention to promote colorectal cancer screening. Journal of clinical oncology : official journal of the American Society of Clinical Oncology Nov 2009 27(32)5370--5375 Population
690.	Personalized medicine (beyond pet biomarkers) 2016 ()1--447 Study design
691.	BMI Is a Risk Factor for Colorectal Cancer Mortality. Digestive diseases and sciences Sep 2017 62(9)2511--2517 Outcome
692.	Physical activity and the gastro-intestinal tract: Responses in health and disease 2016 ()1--222 Outcome
693.	Physical activity and the gastro-intestinal tract: Responses in health and disease 2016 ()1--222 Study design
694.	Randomised controlled trial of a repeated consultation support intervention for patients with colorectal cancer. Psycho-oncology Apr 2019 28(4)702--709 Intervention
695.	Against colorectal cancer in our neighborhoods (ACCION): A comprehensive community-wide colorectal cancer screening intervention for the uninsured in a predominantly Hispanic community Preventive Medicine 2016 91()273--280 Population
696.	Children as Biomarker Orphans: Progress in the Field of Pediatric Biomarkers Journal of Pediatrics 2018 193()14--20.e31 Population
697.	Chemoradiation for advanced primary vulval cancer Cochrane Database of Systematic Reviews 2011 (4) Study design
698.	Interventions for preventing delirium in hospitalised nonâ€ ICU patients Cochrane Database of Systematic Reviews 2016 (3) Study design
699.	EMOTIONAL DISCLOSURE AND SYMPTOMS, EMOTIONAL DISTRESS, HEALTHCARE UTILIZATION, AND QUALITY OF LIFE IN IBS: A CONTROLLED TRIAL Digestive Disease Week Abstracts and Itinerary Planner 2003 2003()T1419--Abstract No. T1419 Outcome
700.	Association of progression-free survival with patient-reported outcomes and survival: results from a randomised phase 3 trial of panitumumab. British journal of cancer Dec 2007 97(11)1469--1474 Outcome
701.	Effect of Colonoscopy Outreach vs Fecal Immunochemical Test Outreach on Colorectal Cancer Screening Completion: A Randomized Clinical Trial. JAMA Sep 2017 318(9)806--815 Population
702.	Biologics or tofacitinib for people with rheumatoid arthritis naive to methotrexate: a systematic review and network metaâ€ analysis Cochrane Database of Systematic Reviews 2017 (5) Study design
703.	Biologics or tofacitinib for people with rheumatoid arthritis unsuccessfully treated with biologics: a systematic review and network metaâ€ analysis Cochrane Database of Systematic Reviews 2017 (3) Study design
704.	Exercise and colorectal cancer: a systematic review and meta-analysis of exercise safety, feasibility and effectiveness 2020 Study design
705.	Antibiotics for meconiumâ€ stained amniotic fluid in labour for preventing maternal and neonatal infections Cochrane Database of Systematic Reviews 2014 (11) Study design
706.	Final analysis of the phase III randomized trial of cetuximab (CET) plus either brivanib alaninate (BRIV) or placebo in patients (pts) with chemotherapy refractory, K-RAS wild-type (WT), metastatic colorectal carcinoma (mCRC): the NCIC Clinical Trials Gro Journal of clinical oncology Outcome
707.	Phase III randomized trial of cetuximab (CET) plus either brivanib alaninate (BRIV) or placebo in patients (pts) with metastatic (MET) chemotherapy refractory K-RAS wild-type (WT) colorectal carcinoma (CRC): the NCIC Clinical Trials Group and AGITG CO.20 Journal of clinical oncology Outcome
708.	A decision aid to support informed choices about bowel cancer screening among adults with low education: randomised controlled trial. BMJ (Clinical research ed.) Oct 2010 341()c5370 Population
709.	Efficacy to effectiveness transition of an Educational Program to Increase Colorectal Cancer Screening (EPICS): study protocol of a cluster randomized controlled trial. Implementation Science Jan 2013 8(1)86 Outcome
710.	Increasing vegetable and fruit intake: Randomized intervention and monitoring in an at-risk population Cancer Epidemiology Biomarkers and Prevention 2000 9(3)307--317 Intervention
711.	Evaluating holistic needs assessment in outpatient cancer care--a randomised controlled trial: the study protocol BMJ open 2015 5(5)e006840 Study design
712.	A family-based multimedia intervention to enhance the uptake of colorectal cancer screening among older South Asian adults in Hong Kong: a study protocol for a cluster randomized controlled trial. BMC public health May 2019 19(1)652 Population
713.	Colorectal cancer screening with odour material by canine scent detection. Gut Jun 2011 60(6)814--819 Population

714.	Fatigue, Quality of Life, and Physical Fitness Following an Exercise Intervention in Survivors of Multiple Myeloma: A Randomised Controlled Trial Using a Zelen Design Blood 2018 132() Population
715.	Colon cancer information as a source of exercise motivation. Psychology {\&} Health Dec 2006 21(6)739--755 Intervention
716.	Randomised controlled trial of a supervised exercise rehabilitation program for colorectal cancer survivors immediately after chemotherapy: study protocol. BMC cancer 2007 7()154 Study design
717.	Exploring Views of Healthcare Professionals, Researchers, and People Living with and beyond Colorectal Cancer on a Healthy-Eating and Active Lifestyle Resource Nutrients 2019 11(10) Outcome
718.	What Happens Now? Psychosocial Care for Cancer Survivors After Medical Treatment Completion Journal of Clinical Oncology 2012 30(11)1215--1220 Study design
719.	Why are some people more successful at lifestyle change than others? Factors associated with successful weight loss in the BeWEL randomised controlled trial of adults at risk of colorectal cancer. The international journal of behavioral nutrition and physical activity 2015 12.87 Population
720.	Effect of evidence based risk information on "informed choice" in colorectal cancer screening: randomised controlled trial BMJ Population
721.	Knowledge, attitudes, and screening practices among older men regarding prostate cancer American Journal of Public Health 2000 90(10)1595--1600 Population
722.	Can targeted intervention in CRC patients' relatives influence screening behaviour? A pilot study. Colorectal disease : the official journal of the Association of Coloproctology of Great Britain and Ireland Feb 2008 10(2)179--186 Intervention
723.	A Study Comparing Colorectal Cancer Screening Techniques. Gastroenterology nursing : the official journal of the Society of Gastroenterology Nurses and Associates 2017 40(2)121--127 Population
724.	Randomized controlled trial of the impact of intensive patient education on compliance with fecal occult blood testing. Journal of general internal medicine Mar 2005 20(3)278--282 Population
725.	Olanzapine for the prevention and treatment of cancerâ€ related nausea and vomiting in adults Cochrane Database of Systematic Reviews 2018 (9) Study design
726.	Oral water soluble contrast for malignant bowel obstruction Cochrane Database of Systematic Reviews 2018 (3) Study design
727.	Nutrients and oxidative stress: Friend or foe? Oxidative Medicine and Cellular Longevity 2018 2018() Outcome
728.	Primary prevention of colorectal cancer: Myth or reality? World Journal of Gastroenterology Nov 2014 20(41)15060--15069 Study design
729.	The Effects of the Nurse Navigation Program in Promoting Colorectal Cancer Screening Behaviors: a Randomized Controlled Trial. Journal of cancer education : the official journal of the American Association for Cancer Education Feb 2020 35(1)112--124 Population
730.	Construction and optimization of gene expression signatures for prediction of survival in two-arm clinical trials. BMC bioinformatics Jul 2020 21(1)333 Study design
731.	Colorectal carcinoma screening among Hispanics and non-Hispanic whites in a rural setting. Cancer Jun 2005 103(12)2491--2498 Population
732.	Finasteride improves the sensitivity of digital rectal examination for prostate cancer detection. The Journal of urology May 2007 177(5)1749--1752 Population
733.	Comparing the use of evidence and culture in targeted colorectal cancer communication for African Americans. Patient education and counseling Dec 2010 81.S22--33 Outcome
734.	A randomized controlled trial of a decision aid for women at increased risk of ovarian cancer. Medical decision making : an international journal of the Society for Medical Decision Making 2006 26(4)360--372 Population
735.	The Next Step Trial: impact of a worksite colorectal cancer screening promotion program. Preventive medicine Mar 1999 28(3)276--283 Population
736.	Interventions for preventing upper gastrointestinal bleeding in people admitted to intensive care units Cochrane Database of Systematic Reviews 2018 (6) Study design
737.	Lessons learned from adapting a cognitive-existential group intervention for fear of cancer recurrence to an individual format Psycho-oncology 2014 23()6 Intervention
738.	Salvage systemic therapy for advanced gastric and oesophagoâ€ gastric junction adenocarcinoma Cochrane Database of Systematic Reviews 2020 (11) Study design
739.	Lay health educators increase colorectal cancer screening among Hmong Americans: A cluster randomized controlled trial. Cancer 2017 123(1)98--106 Population
740.	Adalimumab for maintenance of remission in Crohn's disease Cochrane Database of Systematic Reviews 2020 (5) Study design
741.	Nursing intervention to enhance outpatient chemotherapy symptom management: patient-reported outcomes of a randomized controlled trial Cancer 2015 121(21)3905--3913 Intervention
742.	Psychosocial interventions for informal caregivers of people living with cancer Cochrane Database of Systematic Reviews 2019 (6) Study design

743.	Mifepristone for uterine fibroids Cochrane Database of Systematic Reviews 2012 (8) Study design
744.	Exercise interventions for adults with cancer receiving radiation therapy alone Cochrane Database of Systematic Reviews 2019 (10) Study design
745.	Promoting culturally appropriate colorectal cancer screening through a health educator: a randomized controlled trial. Cancer Sep 2006 107(5)959--966 Population
746.	Promoting culturally appropriate colorectal cancer screening through a health educator: A randomized controlled trial Cancer Sep 2006 107(5)959--966 Population
747.	Development of intervention materials for individuals with limited English proficiency - Lessons learned from Colorectal Cancer Screening in Chinese Americans"" Medical Care 2008 46(9)S51--S61 Intervention
748.	Human microbiota in health and disease: From pathogenesis to therapy 2018 ()1--680 Study design
749.	Obesity as a Disease Medical Clinics of North America 2018 102(1)13--33 Population
750.	Stent placement versus surgical palliation for adults with malignant gastric outlet obstruction Cochrane Database of Systematic Reviews 2018 (5) Study design
751.	Association of Survival With Adherence to the American Cancer Society Nutrition and Physical Activity Guidelines for Cancer Survivors After Colon Cancer Diagnosis: the CALGB 89803/Alliance Trial JAMA oncology 2018 4(6)783--790 Outcome
752.	Minimally invasive surgery versus open surgery for the treatment of solid abdominal and thoracic neoplasms in children Cochrane Database of Systematic Reviews 2015 (1) Population
753.	Blended therapy for fear of cancer recurrence: a clinical case study Psycho-oncology 2015 24()247--Outcome
754.	Nonâ€ surgical interventions for late rectal problems (proctopathy) of radiotherapy in people who have received radiotherapy to the pelvis Cochrane Database of Systematic Reviews 2016 (4) Study design
755.	Multimodal prehabilitation in colorectal cancer patients to improve functional capacity and reduce postoperative complications: the first international randomized controlled trial for multimodal prehabilitation. BMC cancer Jan 2019 19(1)98 Outcome
756.	Design of the Physical exercise during Adjuvant Chemotherapy Effectiveness Study (PACES): a randomized controlled trial to evaluate effectiveness and cost-effectiveness of physical exercise in improving physical fitness and reducing fatigue BMC cancer Dec 2010 10()673 Study design
757.	Interventions for treating sexual dysfunction in patients with chronic kidney disease Cochrane Database of Systematic Reviews 2010 (12) Study design
758.	Nicotinic treatment of post-chemotherapy subjective cognitive impairment: a pilot study. Journal of cancer survivorship : research and practice Outcome
759.	Physical Activity during Cancer Treatment (PACT) Study: design of a randomised clinical trial BMC Cancer Study design
760.	Diet and prostate cancer: Mechanisms of action and implications for chemoprevention Nature Reviews Urology 2010 7(8)442--453 Population
761.	Laparoscopic versus open total mesorectal excision for rectal cancer Cochrane Database of Systematic Reviews 2014 (4) Study design
762.	A randomized controlled trial of a tailored interactive computer-delivered intervention to promote colorectal cancer screening: Sometimes more is just the same Annals of Behavioral Medicine Jun 2011 41(3)284--299 Population
763.	Radical prostatectomy versus deferred treatment for localised prostate cancer Cochrane Database of Systematic Reviews 2020 (6) Study design
764.	Selenium for preventing cancer Cochrane Database of Systematic Reviews 2018 (1) Study design
765.	Patient education for informed decision making about prostate cancer screening: a randomized controlled trial with 1-year follow-up. Annals of family medicine 2003 1(1)22--28 Population
766.	Increasing awareness and knowledge of lifestyle recommendations for cancer prevention in Lynch syndrome carriers: Randomized controlled trial Clinical Genetics 2018 Population
767.	Vascularâ€ endothelialâ€ growthâ€ factor (VEGF) targeting therapies for endocrine refractory or resistant metastatic breast cancer Cochrane Database of Systematic Reviews 2012 (7) Study design
768.	Motivational interviewing and colorectal cancer screening: A peek from the inside out Patient Education and Counseling Aug 2008 72(2)210--217 Population
769.	Transabdominal preâ€ peritoneal (TAPP) vs totally extraperitoneal (TEP) laparoscopic techniques for inguinal hernia repair. Cochrane Database of Systematic Reviews 2005 (1) Outcome
770.	Randomized trial of a decision aid for individuals considering genetic testing for hereditary nonpolyposis colorectal cancer risk. Cancer Sep 2008 113(5)956--965 Population
771.	Promoting use of colorectal cancer screening tests. Can we change physician behavior? Journal of general internal medicine Dec 2005 20(12)1097--1101 Population
772.	Healthy colon, healthy life: a novel colorectal cancer screening intervention. American journal of preventive medicine Jul 2010 39(1)1--14 Population

773. Improving the quality and acceptance of colonoscopy preparation by reinforced patient education with short message service: results from a randomized, multicenter study (PERICLES-II). Gastrointestinal endoscopy Mar 2019 89(3)506--513.e4 Population

774. Effect of WeChat and short message service on bowel preparation: an endoscopist-blinded, randomized controlled trial. European journal of gastroenterology {\&} hepatology Feb 2019 31(2)170--177 Population

775. Health literacy levels and views about being invited to a colorectal cancer screening program. Acta Oncologica Population

776. Communityâ€ based selfâ€ management programs for improving participation in life activities in older adults with chronic conditions Cochrane Database of Systematic Reviews 2012 (9) Study design

777. General practice vs surgical-based follow-up for patients with colon cancer: randomised controlled trial British journal of cancer 2006 94(8)1116--1121 Outcome

778. A mixed methods process evaluation of a print-based intervention supported by internet tools to improve physical activity in UK cancer survivors Public Health 2019 175()19--27 Outcome

779. Inflammation-induced pain sensitization in men and women: does sex matter in experimental endotoxemia? Pain Population

780. Improving colorectal cancer screening by targeting office systems in primary care practices: disseminating research results into clinical practice. Archives of internal medicine Mar 2005 165(6)661--666 Population

781. A randomized comparison of print and web communication on colorectal cancer screening. JAMA internal medicine Jan 2013 173(2)122--129 Population

782. Local anaesthetics and regional anaesthesia versus conventional analgesia for preventing persistent postoperative pain in adults and children Cochrane Database of Systematic Reviews 2018 (6) Study design

783. Quality of Life of Colorectal Cancer Patients in Certified Centers versus Non-Certified Hospitals Onkologie 2012 35(4)162--168 Outcome

784. Gastrointestinal physiology: Development, principles and mechanisms of regulation 2018 ()1--1028 Study design

785. Smartphone education improves embarrassment, bowel preparation, and satisfaction with care in patients receiving colonoscopy: A randomized controlled trail. Medicine Nov 2020 99(46)e23102 Intervention

786. Effect of prehabilitation on objectively measured physical fitness after neoadjuvant treatment in preoperative rectal cancer patients: a blinded interventional pilot study. British journal of anaesthesia 2015 114(2)244--251 Outcome

787. Testing to prevent colon cancer: results from a rural community intervention. Annals of family medicine 2013 11(6)500--507 Intervention

788. Can a tailored telephone intervention delivered by volunteers reduce the supportive care needs, anxiety and depression of people with colorectal cancer? A randomised controlled trial. Psycho-oncology Oct 2012 21(10)1053--1062 Intervention

789. Oral morphine for cancer pain Cochrane Database of Systematic Reviews 2016 (4) Study design

790. Opioids for cancerâ€ related pain in children and adolescents Cochrane Database of Systematic Reviews 2017 (7) Study design

791. Opioids for cancer pain â€ an overview of Cochrane reviews Cochrane Database of Systematic Reviews 2017 (7) Study design

792. Colorectal cancer risk information presented by a nonphysician assistant does not increase screening rates. Canadian family physician Medecin de famille canadien Aug 2014 60(8)731--738 Intervention

793. The Kanker Nazorg Wijzer (Cancer Aftercare Guide) protocol: the systematic development of a web-based computer tailored intervention providing psychosocial and lifestyle support for cancer survivors Bmc Cancer 2015 15() Study design

794. Diet and exercise intervention adherence and health-related outcomes among older long-term breast, prostate, and colorectal cancer survivors. Annals of behavioral medicine : a publication of the Society of Behavioral Medicine Oct 2014 99(2)235--45 Outcome

795. Effect of Increasing Levels of Web-Based Behavioral Support on Changes in Physical Activity, Diet, and Symptoms in Men With Prostate Cancer: Protocol for a Randomized Controlled Trial JMIR RESEARCH PROTOCOLS Nov 2018 7(11) Population

796. Promoting physical activity in patients with colon adenomas: a randomized pilot intervention trial Plos one 2012 7(7)e39719 Population

797. Use of activity tracking in major visceral surgery-the Enhanced Perioperative Mobilization (EPM) trial: Study protocol for a randomized controlled trial Trials Study design

798. Evaluation of a physiatrist-directed prehabilitation intervention in frail patients with colorectal cancer: a randomised pilot study protocol. BMJ open 2017 7(6)e015565 Study design

799. Centralisation of services for gynaecological cancer Cochrane Database of Systematic Reviews 2012 (3) Study design

800. Biofeedback for treatment of chronic idiopathic constipation in adults Cochrane Database of Systematic Reviews 2014 (3) Study design
801. A blended knowledge translation initiative to improve colorectal cancer staging [ISRCTN56824239]. BMC health services research Jan 2006 6()4 Outcome
802. A randomized controlled trial to improve lymph node assessment in stage II colon cancer. Archives of surgery (Chicago, Ill. : 1960) Nov 2008 143(11)1050--5; Outcome
803. What does Medicaid expansion mean for cancer screening and prevention? Results from a randomized trial on the impacts of acquiring Medicaid coverage. Cancer Mar 2016 122(5)791--797 Outcome
804. Chinese medical herbs for chemotherapy side effects in colorectal cancer patients Cochrane Database of Systematic Reviews 2005 (1) Study design
805. Surufatinib in advanced extrapancreatic neuroendocrine tumours (SANET-ep): a randomised, double-blind, placebo-controlled, phase 3 study. The Lancet. Oncology Nov 2020 21(11)1500--1512 Outcome
806. Spiritual and religious interventions for adults with cancer and their carers: an overview of systematic reviews Cochrane Database of Systematic Reviews 2020 (7) Study design
807. Chinese herbal medicines for induction of remission in advanced or late gastric cancer Cochrane Database of Systematic Reviews 2013 (4) Study design
808. Effect of team support training on rehabilitation of stoma patients with rectal cancer World chinese journal of digestology 2015 23(7)1196--1201 Intervention
809. Interventions for treating gas gangrene Cochrane Database of Systematic Reviews 2015 (12) Study design
810. Occupational therapy to improve quality of life for colorectal cancer survivors: a randomized clinical trial Supportive Care in Cancer 2020 28(3)1503--1511 Intervention
811. Dietary fibre for the prevention of recurrent colorectal adenomas and carcinomas Cochrane Database of Systematic Reviews 2017 (1) Study design
812. Multicenter randomized trial of centralized nurse-led telephone-based care coordination to improve outcomes after surgical resection for colorectal cancer: the CONNECT intervention. Journal of clinical oncology : official journal of the American Society of Clinical Oncology Oct 2013 31(28)3585--3591 Outcome
813. Patient education for colon cancer screening: a randomized trial of a video mailed before a physical examination Annals of internal medicine 2004 141(9)683--692 Population
814. Hydrocolloid dressing as a prophylactic use for hand-foot skin reaction induced by multitargeted kinase inhibitors: protocol of a phase 3 randomised self-controlled study. BMJ open Oct 2020 10(10)e038276 Study design
815. Moxibustion for alleviating side effects of chemotherapy or radiotherapy in people with cancer Cochrane Database of Systematic Reviews 2018 (11) Study design
816. Simethicone improves bowel cleansing with low-volume polyethylene glycol: a multicenter randomized trial Endoscopy 2018 50(4)412--422 Outcome
817. Does a pulmonary rehabilitation based ERAS program (PREP) affect pulmonary complication incidence, pulmonary function and quality of life after lung cancer surgery? Study protocol for a multicenter randomized controlled trial Bmc Pulmonary Medicine 2020 20(1) Study design
818. Trained facilitators' experiences with structured advance care planning conversations in oncology: an international focus group study within the ACTION trial. BMC Cancer Oct 2019 19(1)1--13 Outcome
819. The role of calcium in peri- and postmenopausal women: 2006 Position statement of the North American Menopause Society Menopause 2006 13(6)862--877 Population
820. Evaluation of supervised multimodal prehabilitation programme in cancer patients undergoing colorectal resection: a randomized control trial. Acta oncologica (Stockholm, Sweden) 2018 57(6)849--859 Baseline PA greater than 150min/week
821. A randomized phase II dose-response exercise trial among colon cancer survivors: Purpose, study design, methods, and recruitment results. Contemporary clinical trials 2016 47()366--375 Study design
822. Effects of exercise on circulating tumor cells among patients with resected stage I-III colon cancer. PloS one 2018 13(10)e0204875 Outcome
823. Building a physical activity intervention into clinical care for breast and colorectal cancer survivors in Wisconsin: a randomized controlled pilot trial Journal of cancer survivorship 2019 13(4)593--602 Population
824. A randomized controlled bicenter trial of yoga for patients with colorectal cancer. Psycho-oncology Apr 2016 25(4)412--420 Outcome
825. A pilot study on the motivational effects of an internet-delivered physical activity behaviour change programme in Nova Scotian cancer survivors Psychology {\&} Health Feb 2017 32(2)234--252 Outcome
826. A Web-Based and Print-Based Computer-Tailored Physical Activity Intervention for Prostate and Colorectal Cancer Survivors: A Comparison of User Characteristics and Intervention Use. Journal of medical Internet research 2017 19(8)e298 Baseline PA greater than 150min/week

827. Short-term efficacy of a computer-tailored physical activity intervention for prostate and colorectal cancer patients and survivors: a randomized controlled trial. The international journal of behavioral nutrition and physical activity 2018 15(1)106 Baseline PA greater than 150min/week
828. Phase II Feasibility Study of a Weight Loss Intervention in Female Breast and Colorectal Cancer Survivors (SWOG S1008). Obesity (Silver Spring, Md.) 2018 26(10)1539--1549 Outcome
829. Associations of Physical Activity With Survival and Progression in Metastatic Colorectal Cancer: Results From Cancer and Leukemia Group B (Alliance)/SWOG 80405 Journal of Clinical Oncology 2019 37(29)2620--+ Outcome
830. Message Framing and Physical Activity Promotion in Colorectal Cancer Survivors. Oncology nursing forum Nov 2016 43(6)697--705 Comparator
831. Is referral of postsurgical colorectal cancer survivors to cardiac rehabilitation feasible and acceptable? A pragmatic pilot randomised controlled trial with embedded qualitative study. BMJ open 2016 6(1)e009284 Outcome
832. At cancer diagnosis ? A ?window of opportunity? for behavioural change towards physical activity for colon and breast cancer patients http://www.who.int/trialsearch/Trial2.aspx?TrialID=ISRCTN24901641 2012 () Outcome
833. Dietary and physical activity interventions for colorectal cancer survivors: a randomized controlled trial Scientific reports 2018 8(1)5731 Baseline PA greater than 150min/week
834. Impact of a telephone-based physical activity intervention upon exercise behaviors and fitness in cancer survivors enrolled in a cooperative group setting. Breast cancer research and treatment 40940 132(1)205-13 Study design
835. A randomized controlled trial of a multiple health behavior change intervention delivered to colorectal cancer survivors: Effects on sedentary behavior Cancer 2014 () Duplicate
836. A randomized controlled trial of WATAAP to promote physical activity in colorectal and endometrial cancer survivors Psycho-Oncology Jul 2019 28(7)1420--1429 Outcome
837. Prehabilitation is feasible in patients with rectal cancer undergoing neoadjuvant chemoradiotherapy and may minimize physical deterioration: results from the REx trial Colorectal disease 2019 21(5)548--562 Baseline PA greater than 150min/week
838. CRIB--the use of cardiac rehabilitation services to aid the recovery of patients with bowel cancer: a pilot randomised controlled trial (RCT) with embedded feasibility study. BMJ open 2014 4(2)e004684 Baseline PA greater than 150min/week
839. Health Education Materials With or Without a Physical Activity Program for Patients Who Have Undergone Treatment for High-Risk Stage II or Stage III Colon Cancer https://clinicaltrials.gov/show/NCT00819208 2009 () Duplicate
840. Diet and Physical Activity Intervention in CRC Survivors https://clinicaltrials.gov/show/NCT01708824 2012 () Outcome
841. The Effects of a 9 Week Exercise Programme on Fitness and Quality of Life in Rectal Cancer Patients After Chemoradiotherapy and Before Surgery https://clinicaltrials.gov/show/NCT01914068 2013 () Outcome
842. Prospective Randomised Trial of Exercise and / or Antioxidants in COlorectal Cancer Patients Undergoing Surgery https://clinicaltrials.gov/show/NCT02264496 2014 () Outcome
843. Physical Activity in Patients With Metastatic Colorectal Cancer Who Receive Palliative First-line Chemotherapy https://clinicaltrials.gov/show/NCT02597075 2015 () Outcome
844. The Feasibility of an Active Lifestyle Programme in Patients Recovering From Colorectal Cancer https://clinicaltrials.gov/show/NCT02751892 2016 () Outcome
845. Self-monitoring and Reminder Texts to Increase Physical Activity After Cancer: a Pilot Randomized Controlled Trial https://clinicaltrials.gov/show/NCT02966054 2016 () Duplicate
846. Motivational Interviewing to Increase Physical Activity Behaviour in Cancer Patients https://clinicaltrials.gov/show/NCT03210129 2017 Outcome
847. Exercise to Reduce Chemotherapy-Induced Peripheral Neuropathy https://clinicaltrials.gov/show/NCT03515356 2018 Outcome
848. A Web-based Tailored Health Behavior Intervention for Breast and Colon Cancer Survivors (eWellness) https://clinicaltrials.gov/show/NCT04188002 2019 () Outcome
849. The effect of oncologists' exercise recommendations on the level of exercise and quality of life in survivors of breast and colorectal cancer: A randomized controlled trial Cancer 2015 121(16)2740--2748 Population
850. A brief yoga intervention implemented during chemotherapy: A randomized controlled pilot study. Complementary therapies in medicine Apr 2016 25()139--142 Outcome
851. A self-regulation-based intervention to increase physical activity in cancer patients Psychology, health & medicine 2016 21(2)163--175 Population
852. A randomized controlled trial of physical activity, dietary habit, and distress management with the Leadership and Coaching for Health (LEACH) program for disease-free cancer survivors Bmc Cancer 2017 17 Population

853. Efficacy of health coaching and a web-based program on physical activity, weight, and distress management among cancer survivors: A multi-centered randomised controlled trial Psycho-Oncology 2020 29(7)1105--1114 Outcome
854. A randomized trial of tailoring and motivational interviewing to promote fruit and vegetable consumption for cancer prevention and control." Annals of Behavioral Medicine 38, no. 2 (2009): 71-85. Baseline PA not meeting threshold criteria
855. Effects of Community-based Exercise Prehabilitation for Patients Scheduled for Colorectal Surgery With High Risk for Postoperative Complications: Results of a Randomized Clinical Trial Annals of Surgery 2022 275(2)E299--E306 .Study design
856. Mobile health technologies supporting colonoscopy preparation: A systematic review and meta-analysis of randomized controlled trials PLoS ONE 2021 16(44623)e0248679 .Study design
857. The influence of telehealth-based cancer rehabilitation interventions on disability: a systematic review Journal of Cancer Survivorship 2022 () .Study design
858. Recruitment strategies and design considerations in a trial of resistance training to prevent dose-limiting toxicities in colon cancer patients undergoing chemotherapy Contemporary Clinical Trials feb 2021 101()106242 .Study design
859. The Role of Diet in Prognosis among Cancer Survivors: A Systematic Review and Meta-Analysis of Dietary Patterns and Diet Interventions Nutrients jan 2022 14(2) .Study design
860. Functional capacity of prediabetic patients: effect of multimodal prehabilitation in patients undergoing colorectal cancer resection Acta Oncologica 2021 60(8)1025--1031 .Intervention
861. Predictors of Response to an Evidence-Based Behavioral Cancer Pain Management Intervention: An Exploratory Analysis From a Clinical Trial Journal of Pain and Symptom Management 2021 62(2)391--399 .Intervention
862. Cognitive skill training improves memory, function, and use of cognitive strategies in cancer survivors Supportive Care in Cancer jan 2022 30(1)711--720 .Population
863. A multilevel, low literacy dual language intervention to promote colorectal cancer screening in community clinics in Florida: A randomized controlled trial Preventive Medicine may 2022 158()107021 .Intervention
864. The Patient Navigator: Can a systematically developed online health information tool improve patient participation and outcomes related to the consultation in older patients newly diagnosed with colorectal cancer? BMC Cancer jan 2022 22(1)109 .Intervention
865. Does exercise intensity matter for fatigue during (neo-)adjuvant cancer treatment? The Phys-Can randomized clinical trial Scandinavian Journal of Medicine & Science in Sports () .Outcome
866. Effects of Cognitive Training and Social Support on Cancer-Related Fatigue and Quality of Life in Colorectal Cancer Survivors: A Systematic Review and Meta-Analysis Integrative Cancer Therapies 2022 21() .Study design
867. Impact of a health literacy intervention combining general practitioner training and a consumer facing intervention to improve colorectal cancer screening in underserved areas: protocol for a multicentric cluster randomized controlled trial BMC Public Health () .Population
868. Protein Recommendation to Increase Muscle (PRIMe): Study protocol for a randomized controlled pilot trial investigating the feasibility of a high protein diet to halt loss of muscle mass in patients with colorectal cancer Clinical Nutrition ESPEN feb 2021 41()175--185 .Study design
869. Encouraging participation in colorectal cancer screening for people with schizophrenia: A randomized controlled trial Acta Psychiatrica Scandinavica oct 2021 144(4)318--328 .Population
870. Older frail prehabilitated patients who cannot attain a 400 m 6-min walking distance before colorectal surgery suffer more postoperative complications European Journal of Surgical Oncology 2021 47(4)874--881 .Outcome
871. Examining strategies for addressing high levels of 'I don't know' responding to risk perception questions for colorectal cancer and diabetes: an experimental investigation Psychology and Health jul 2021 36(7)862--878 .Intervention
872. SupPoRtive Exercise Programmes for Accelerating REcovery after major ABdominal Cancer surgery trial (PREPARE-ABC): Study protocol for a multicentre randomized controlled trial Colorectal Disease oct 2021 23(10)2750--2760 .Study design
873. Patient-led follow-up reduces outpatient doctor visits and improves patient satisfaction. One-year analysis of secondary outcomes in the randomised trial Follow-Up after Rectal CAncer (FURCA) Acta Oncologica sep 2021 60(9)1130--1139 .Intervention
874. Economic evaluation of web- versus telephone-based interventions to simultaneously increase colorectal and breast cancer screening among women Cancer Prevention Research sep 2021 14(9)905--916 .Population
875. Changes in DNA methylation after 6-week exercise training in colorectal cancer survivors: A preliminary study Asia-Pacific Journal of Clinical Oncology feb 2022 18(1)52--60 .Outcome
876. Physical Activity Interventions for Colorectal Cancer Survivors: A Systematic Review and Meta-analysis of Randomized Controlled Trials Cancer nursing 2021 44(6)E414--E428 .Study design

877. Quality of life and patient satisfaction after single- and multiport laparoscopic surgery in colon cancer: a multicentre randomised controlled trial (SIMPLE Trial) Surgical Endoscopy nov 2021 35(11)6278--6290 .Outcome
878. Feasibility, engagement, and acceptability of a behavioral pain management intervention for colorectal cancer survivors with pain and psychological distress: data from a pilot randomized controlled trial Supportive Care in Cancer sep 2021 29(9)5361--5369 .Intervention
879. Predictors of Colorectal Cancer Screening Among African American Men Living with HIV Journal of Community Health 2021 46(6)1099--1106 .Population
880. Effectiveness of preoperative group education for patients with colorectal cancer: managing expectations Supportive Care in Cancer sep 2021 29(9)5263--5271 .Intervention
881. Randomized controlled trial of storytelling vs didactic education effects on dietary colorectal cancer risk behaviors among latinxs Journal of Health Care for the Poor and Underserved 2021 32(3)1531--1553 .Intervention
882. General Health Checks in Adult Primary Care: A Review JAMA - Journal of the American Medical Association jun 2021 325(22)2294--2306 .Population
883. Effect of home-based prehabilitation in an enhanced recovery after surgery program for patients undergoing colorectal cancer surgery during the COVID-19 pandemic Supportive Care in Cancer 2021 29(12)7785--7791 .Outcome
884. Assessing the effectiveness of a community health advisor plus screen to save educational intervention on stool-based testing adherence in an African American safety net clinic population: study protocol for a randomized pragmatic trial Trials feb 2022 23(1)151 .Intervention
885. The FamilyTalk randomized controlled trial: patient-reported outcomes in clinical genetic sequencing for colorectal cancer Cancer Causes and Control may 2021 32(5)483--492 .Intervention
886. Effect of self-regulatory behaviour change techniques and predictors of physical activity maintenance in cancer survivors: a 12-month follow-up of the Phys-Can RCT BMC Cancer nov 2021 21(1)1272 .Duplicate
887. Interventions to improve physical activity in colorectal cancer survivors: protocol for a systematic review and meta-analysis of randomized controlled trials Journal of Advanced Nursing sep 2021 77(9)3921--3932 .Study design
888. Impact of non-pharmacological interventions on quality of life, anxiety, and depression scores in patients with colorectal cancer: a systematic review and meta-analysis of randomized controlled trials Supportive Care in Cancer oct 2021 29(10)5635--5652 .Intervention
889. Patient and patient group engagement in cancer clinical trials: A stakeholder charter apr 2021 28(2)1447--1458 .Population
890. Colorectal cancer lifetime risk accuracy and behavior change intentions before and after risk assessment Cancer Causes and Control apr 2021 32(4)423--428 .Outcome
891. The Effects of Personalizing Colorectal Cancer Risk Communication on Risk Perceptions and Health Behavior Intentions: a Randomized Trial of Average-Risk Adults Journal of Cancer Education aug 2021 36(4)719--727 .Intervention
892. Feasibility, Safety, and Preliminary Efficacy of Exercise During and After Neoadjuvant Rectal Cancer Treatment: A Phase II Randomized Controlled Trial Clinical Colorectal Cancer 2021 20(3)216--226 .Outcome
893. Communicating risk to promote colorectal cancer screening: a multi-method study to test tailored versus targeted message strategies Health Education Research mar 2022 37(2)79--93 .Population
894. Roux-en-Y versus Billroth-I reconstruction after distal gastrectomy for gastric cancer Cochrane Database of Systematic Reviews sep 2021 2021(9)CD012998 .Population
895. Effectiveness of an independent physical activity programme in improving physical activity amongst breast and colorectal cancer survivors: Study protocol for a randomized controlled trial Medical Journal of Malaysia jul 2021 76(4)480--487 .Study design
896. Effect of Short-term Homebased Pre- and Postoperative Exercise on Recovery After Colorectal Cancer Surgery (PHYSSURG-C): A Randomized Clinical Trial Annals of Surgery () .Outcome
897. The Effect of Progressive Relaxation Exercise on Physiological Parameters, Pain and Anxiety Levels of Patients Undergoing Colorectal Cancer Surgery: A Randomized Controlled Study Journal of Perianesthesia Nursing apr 2021 37(2)238--246 .Outcome
898. AGA Clinical Practice Update on Medical Management of Colonic Diverticulitis: Expert Review Gastroenterology feb 2021 160(3)906--911.e1 .Intervention
899. A Low Literacy, Multimedia Health Information Technology Intervention to Enhance Patient-Centered Cancer Care in Safety Net Settings Increased Cancer Knowledge in a Randomized Controlled Trial Cancer Control 2021 28()10732700000000000 .Population
900. Implementation of the pre-operative rehabilitation recovery protocol and its effect on the quality of recovery after colorectal surgeries Chinese Medical Journal 2021 134(23)2865--2873 .Outcome
901. Participation in Competing Strategies for Colorectal Cancer Screening: A Randomized Health Services Study (PICCOLINO Study) Gastroenterology mar 2021 160(4)1097--1105 .Population

902. A Meta-analysis on association between statins and colorectal cancer Zhonghua liu xing bing xue za zhi = Zhonghua liuxingbingxue zazhi feb 2021 42(2)343--350 .Study design
903. Pilot Randomized Controlled Trial of Feasibility, Acceptability, and Preliminary Efficacy of a Web-Based Physical Activity and Sedentary Time Intervention for Survivors of Physical Inactivity-Related Cancers International Journal of Behavioral Medicine apr 2022 29(2)220--229 .Population
904. Health services research in colorectal cancer: a quasi-experimental interventional pilot study on in- and outpatient oncology Journal of Cancer Research and Clinical Oncology 147 () .Intervention
905. Visceral adiposity and inflammatory bowel disease International Journal of Colorectal Disease nov 2021 36(11)2305--2319 .Outcome
906. WASh multicentre randomised controlled trial: Water-assisted sigmoidoscopy in English NHS bowel scope screening Gut may 2021 70(5)845--852 .Population
907. A Randomized Controlled Pilot Study of Yoga Skills Training Versus an Attention Control Delivered During Chemotherapy Administration Journal of Pain and Symptom Management 2022 63(1)23--32 .Outcome
908. Who makes it all the way? Participants vs. decliners, and completers vs. drop-outs, in a 6-month exercise trial during cancer treatment. Results from the Phys-Can RCT Supportive Care in Cancer () .Duplicate
909. Association of a prehabilitation program with anxiety and depression before colorectal surgery: a post hoc analysis of the pERACS randomized controlled trial Langenbeck's Archives of Surgery 2021 406(5)1553--1561 .Outcome
910. Effectiveness of self-help workbook intervention on quality of life in cancer patients receiving chemotherapy: results of a randomized controlled trial BMC Cancer may 2021 21(1)588 .Outcome
911. Risk factors for poor health-related quality of life in patients with colon cancer include stoma and smoking habits Health and Quality of Life Outcomes 2021 19(1) .Outcome
912. Effects of physical exercise on natural killer cell activity during (neo)adjuvant chemotherapy: A randomized pilot study Physiological Reports 2021 9(11)e14919 .Outcome
913. Precision medicine: preliminary results from the Initiative for Molecular Profiling and Advanced Cancer Therapy 2 (IMPACT2) study npj Precision Oncology mar 2021 5(1)21 .Intervention
914. Systematic review on optical diagnosis of early gastrointestinal neoplasia Journal of Clinical Medicine 2021 10(13) .Study design
915. Study protocol for the Exercising Together\textcopyright trial: a randomized, controlled trial of partnered exercise for couples coping with cancer Trials aug 2021 22(1)579 .Study design
916. Short-term outcomes of complete mesocolic excision versus D2 dissection in patients undergoing laparoscopic colectomy for right colon cancer (RELARC): a randomised, controlled, phase 3, superiority trial The Lancet Oncology mar 2021 22(3)391--401 .Intervention
917. Randomized Controlled Trial of Personalized Colorectal Cancer Risk Assessment vs Education to Promote Screening Uptake The American journal of gastroenterology feb 2021 116(2)391--400 .Population
918. Effects of supervised aerobic exercise on cardiorespiratory fitness and patient-reported health outcomes in colorectal cancer patients undergoing adjuvant chemotherapyâ€"a pilot study Supportive Care in Cancer 2022 30(3)1945--1955 .Outcome

Table S4: Extent of theory use across relevant articles							
Item	*Courneya et al. 2016*	*Hawkes et al. 2013*	*Pinto et al.2013*	*Van Blarigan et al. 2019*	*Van Vulpen et al. 2016*	*Van Waart et al. 2018*	*Watson et al. 2015*
**Theory/model mentioned**	1	1	1	1	1	1	1
**Targeted construct mentioned as predictor of behavior**	0	0	0	1	0	2	1
**Intervention based on single theory**	1	0	1	1	1	1	0
**Theory/predictors used to select recipients for the intervention**	0	0	0	0	0	2	0
**Theory/predictors used to select /develop intervention techniques**	1	1	1	1	1	1	1
**Theory/predictors used to tailor intervention techniques to recipients**	0	1	1	1	1	1	1
**All intervention techniques are explicitly linked to at least one theory-relevant construct/predictor**	2	2	2	2	2	2	1
**At least one, but not all, of the intervention techniques are explicitly linked to at least one theory relevant construct/predictor**	0	1	1	1	0	1	1
**Group of techniques are linked to a group of constructs/predictors**	2	2	2	2	2	2	2
**All theory-relevant constructs/predictors are explicitly linked to at least one intervention technique**	2	2	2	2	2	2	2
**At least one, but not all, of the theory relevant constructs/predictors are explicitly linked to at least one intervention technique**	1	1	1	1	0	2	1
**Theory-relevant constructs/predictors are measured**	0	0	0	0	0	0	0
**Quality of measures**	0	0	0	0	0	0	0
**Randomization of participants to condition**	1	1	1	1	1	1	1

**Changes in measured theory relevant constructs/predictor**	0	0	0	0	0	0	2
**Mediational analysis of construct/s/predictors**	0	0	0	0	0	0	0
**Results discussed in relation to theory**	0	0	0	0	0	0	0
**Appropriate support for theory**	0	0	0	0	0	0	0
**Results used to refine theory**	0	0	0	0	0	0	0

Notes: 1: Yes (present); 0: No(absent); 2: Unclear

Table S5: Overview of the behavior change techniques used in our final sample of articles

Authors/ year	Intervention	Control
**Backman et al. 2014**	*1.1: Goal setting (behaviour)*<br>*1.3: Goal setting (outcome)*<br>*1.4: Action planning*<br>*2.1 Other(s) monitoring with awareness*<br>*3.1: Social support (general)*<br>*6.1: Modelling of the behaviour*	*n/a*
**Bourke et al. 2011**	*1.1: Goal setting (behaviour)*<br>*1.3: Goal setting (outcome)*<br>*1.4: Action planning*<br>*4.1: Instruction on how to perform a behaviour*<br>*2.1 Other(s) monitoring with awareness*<br>*2.3: Self-monitoring of behaviour*<br>*8.6: Generalization of a target behaviour*<br>*9.1: Persuasive argument/Credible source*	*n/a*
**Brown et al. 2018**	*1.1: Goal setting (behaviour)*<br>*1.2: Problem solving*<br>*1.3: Goal setting (outcome)*<br>*2.2: Feedback on behaviour*<br>*3.1: Social support (general)*<br>*3.3: Social support (emotional)*<br>*4.1: Instruction on how to perform a behaviour*<br>*5.1: Health consequences*<br>*8.7: Graded tasks*<br>*9.1: Persuasive argument/Credible source*<br>*10.4: Social reward*<br>*12.1: Restructuring the physical environment (they provided treadmill)*	*n/a*
**Carli et al. 2020**	*1.1: Goal setting (behaviour)*<br>*1.3: Goal setting (outcome)*<br>*1.4: Action planning*<br>*1.5: Review behaviour goal(s)*<br>*4.1: Instruction on how to perform a behaviour*	*After waitlist:*<br>*1.1: Goal setting (behaviour)*<br>*1.3: Goal setting (outcome)*<br>*1.4: Action planning*<br>*1.5: Review behaviour goal(s)*<br>*4.1: Instruction on how to perform a behaviour*
**Christensen et al. 2019**	*1.1: Goal setting (behaviour)*<br>*1.3: Goal setting (outcome)*<br>*1.4: Action planning*<br>*1.5: Review behaviour goal(s)*<br>*1.7: Review outcome goal(s)*<br>*2.5: Monitoring of outcome(s) of behaviour without feedback*<br>*4.1. Instructions on how to perform the behaviour.*<br>*4.2: Information about antecedents*	*n/a*

	8.7: Graded tasks	
**Courneya et al. 2003**	*1.1: Goal setting (behaviour)*<br>*1.3: Goal setting (outcome)*<br>*1.7. Review outcome goal(s)*<br>*2.2. Feedback on behaviour*<br>*4.1. Instructions on how to perform the behaviour.*<br>*8.7: Graded tasks*<br>*9.1: Persuasive argument/Credible source*	*n/a*
**Courneya et al. 2016**	*1.1: Goal setting (behaviour)*<br>*1.2: Problem solving*<br>*1.3: Goal setting (outcome)*<br>*1.4: Action planning*<br>*1.5: Review of behaviour goal*<br>*1.6: Discrepancy between actual behaviour and goal standard*<br>*1.7: Review outcome goal(s)*<br>*2.2: Feedback on behaviour*<br>*2.3: Self-monitoring of behaviour*<br>*3.1: Social support (general)*<br>*3.2: Social support (practical)*<br>*3.3: Social support (emotional)*<br>*4.1: Instruction on how to perform behaviour*<br>*5.1: Health consequences*<br>*8.1: Behaviour practice*<br>*8.7: Graded tasks*<br>*9.1: Persuasive argument/Credible source*<br>*12.1: Restructuring the physical environment*	*5.1: Health consequences*
**Demark-Wahnefried et al. 2012**	*1.1: Goal setting (behaviour)*<br>*1.2: Problem solving*<br>*1.5: Review behaviour goal(s)*<br>*1.6: Discrepancy between current behaviour and goal standard*<br>*2.2. Feedback on behaviour*<br>*2.3. Self-monitoring of behaviour*<br>*3.1. Social support (unspecified)*<br>*4.1: Instruction on how to perform behaviour*<br>*7.1: Prompts/cues*<br>*10.3: Non-specific reward*<br>*12.1: Restructuring the physical environment*	*n/a*
**Hawkes et al. 2013**	*1.1: Goal setting (behaviour)*<br>*1.2: Problem solving*<br>*1.4: Action planning*<br>*1.5: Review of behaviour goal*<br>*2.2: Feedback on behaviour*<br>*2.3: Self-monitoring of behaviour*<br>*5.1: Health consequences*<br>*9.1: Persuasive argument/Credible source*	*5.1: Health consequences*
**Young Kim et al. 2019**	*1.1: Goal setting (behaviour)*<br>*1.2: Problem solving*	*n/a*

	1.3: Goal setting (outcome)<br>*1.4: Action planning*<br>*1.5: Review behaviour goal(s)*<br>*2.3: Self-monitoring of behaviour*<br>*4.1: Instruction on how to perform the behaviour*<br>*5.1: Health consequences*<br>*6.1: Modelling of the behaviour*<br>*7.1: Prompts/cues*<br>*8.7: Graded tasks*<br>*8.6: Generalization of a target behaviour*<br>*9.1: Persuasive argument/Credible source*	
**Kyung Lee et al. 2017**	*1.1: Goal setting (behaviour)*<br>*1.2: Problem solving*<br>*1.3: Goal setting (outcome)*<br>*1.4: Action planning*<br>*1.5: Review behaviour goal(s)*<br>*2.3: Self-monitoring of behaviour*<br>*4.1: Instruction on how to perform the behaviour*<br>*5.1: Health consequences*<br>*6.1: Modelling of the behaviour*<br>*7.1: Prompts/cues*<br>*8.7: Graded tasks*<br>*8.6: Generalization of a target behaviour*<br>*9.1: Persuasive argument/Credible source*	*n/a*
**Kyung Lee et al 2018**	*1.1: Goal setting (behaviour)*<br>*1.3: Goal setting (outcome)*<br>*2.3: Self-monitoring of behaviour*<br>*4.1: Instruction on how to perform the behaviour*<br>*5.1: Health consequences*<br>*7.1: Prompts/cues*	*n/a*
**Mayer et al. 2018**	*1.1: Goal setting (behaviour)*<br>*1.3: Goal setting (outcome)*<br>*1.4: Action planning*<br>*2.3: Self-monitoring of behaviour*<br>*2.4: Self-monitoring of outcome(s) of behaviour*<br>*5.1: Health consequences*<br>*6.1: Modelling of the behaviour*	*5.1: Health consequences*
**Pinto et al. 2011**	*1.1: Goal setting (behaviour)*<br>*1.2: Problem solving*<br>*1.3: Goal setting (outcome)*<br>*1.4: Action planning*<br>*2.2: Feedback on behaviour*<br>*2.3: Self-monitoring of behaviour*<br>*2.4: Self-monitoring of outcome(s) of behaviour*<br>*5.1: Health consequences*<br>*8.7: Graded tasks*<br>*14.6: Discrimination training*<br>*14.8: Differential reinforcement*	*5.1: Health consequences*

**Van Blarigan et al. 2019**	*1.1: Goal setting (behaviour)*<br>*1.4: Action planning*<br>*2.3: Self-monitoring of behaviour*<br>*4.1: Instruction on how to perform the behaviour*<br>*5.1: Health consequences*<br>*7.1: Prompts/cues*	*5.1: Health consequences*
**Van Vulpen et al. 2015**	*1.1: Goal setting (behaviour)*<br>*1.3: Goal setting (outcome)*<br>*1.4: Action planning*<br>*1.5: Review behaviour goal(s)*<br>*2.3: Self-monitoring of behaviour*<br>*4.1: Instruction on how to perform the behaviour*	*n/a*
**Van Waart et al. 2018**	*1.1: Goal setting (behaviour)*<br>*1.4: Action planning*<br>*1.2: Problem solving*<br>*1.3: Goal setting (outcome)*<br>*1.4: Action planning*<br>*2.3: Self-monitoring of behaviour*<br>*6.1: Modelling of the behaviour*<br>*8.1: Behavioural practice/rehearsal*	*5.1: Health consequences*
**Watson et al. 2015**	*1.1: Goal setting (behaviour)*<br>*1.3: Goal setting (outcome)*<br>*1.4: Action planning*<br>*4.1: Instruction on how to perform the behaviour*<br>*5.1: Health consequences*<br>*8.6: Generalization of a target behaviour*<br>*9.1: Persuasive argument/Credible source*<br>*12.1: Restructuring the physical environment*	
**Molenaar et al. 2023**	*1.1: Goal setting (behaviour)*<br>*1.3: Goal setting (outcome)*<br>*1.4: Action planning*<br>*1.5: Review behaviour goal(s)*<br>*4.1: Instruction on how to perform a behaviour*	

Notes: n/a: not available

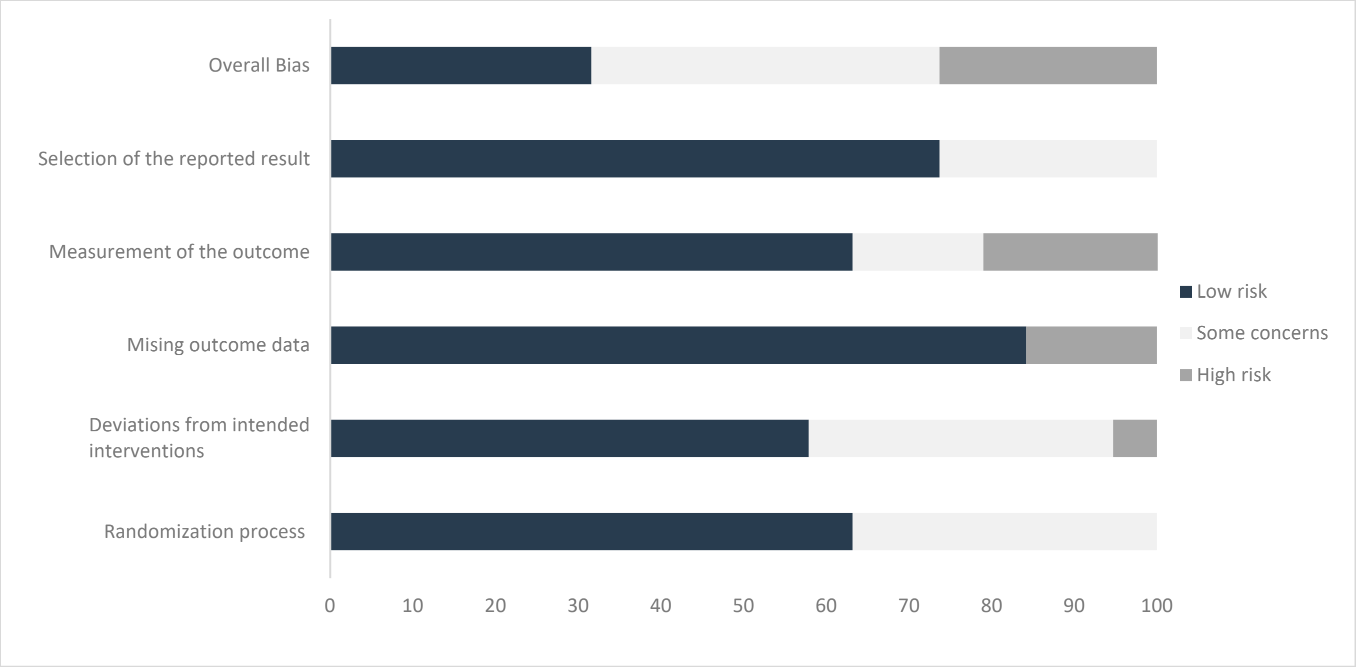

Figure S1: Pooled risk of bias results using the Cochrane Risk of Bias Assessment v2.0 Instrument.

Study	Randomization process	Deviations from the intended interventions	Missing outcome data	Measurement of the outcome	Selection of the reported result	Overall
Backman et al. 2014	!	+	+	!	!	!
Bourke et al. 2011	+	!	+	!	!	!
Brown et al. 2018	+	+	+	+	+	+
Carli et al. 2020	+	+	+	+	+	+
Christensen et al. 2019	+	!	+	+	+	!
Courneya et al. 2003	+	+	+	+	+	+
Demark-Wahnefried et al. 2012	+	+	+	+	+	+
Young Kim et al. 2019	!	+	-	-	!	-
Kyung Lee et al. 2017	!	!	+	-	!	-
Kyung Lee et al. 2018	!	!	-	-	+	-
Mayer et al. 2018	!	+	+	+	+	!
Pinto et al. 2013	!	!	+	+	+	!
Van Blaringan et al. 2019	+	-	+	+	+	-
Van Vulpen et al. 2016	+	+	-	-	+	-
Van Waart et al. 2018	+	!	+	+	+	!
Watson et al. 2015	+	+	+	+	+	+
Courneya et al. 2016	+	+	+	+	+	+
Hawkes et al.2013	+	+	+	!	+	!
Molenaar et al. 2023	+	+	+	+	+	+

+ Low risk
! Some concerns
- High risk

Figure S2: Study level risk of bias

Table S6: GRADE results

Certainty assessment							№ of patients		Effect		Certainty	Importance
№ of studies	Study design	Risk of bias	Inconsistency	Indirectness	Imprecision	Other considerations	Physical activity interventions	Usual care	Relative (95% CI)	Absolute (95% CI)		
**Standardized Mean Effect size of changes in moderate-and-vigorous PA (Atheoretical interventions) (follow-up: range 4 weeks to 48 weeks; assessed with: Change outcome differences in PA between the exercise and control groups)**												
10	randomized trials	serious[a]	very serious[b]	not serious	not serious	publication bias strongly suspected strong association dose response gradient[c]	468	427	-	SMD **0.59 SD higher** (0.07 higher to 1.11 higher)	⊕⊕◯◯ Low	CRITICAL
**Standardized Mean Effect size of changes in moderate-and-vigorous PA (Theory-based interventions) (follow-up: range 12 weeks to 48 weeks; assessed with: Change outcome differences in PA between the exercise and control groups)**												
6	randomized trials	serious[a]	serious[d]	not serious	serious[e]	all plausible residual confounding would reduce the demonstrated effect[b]	368	380	-	SMD **0.27 SD higher** (0.08 lower to 0.62 higher)	⊕⊕◯◯ Low	CRITICAL

CI: confidence interval; SMD: standardised mean difference

Explanations

a. Uncertainty regarding the measurement of outcome was very serious. This coupled with uncertainty with respect to unrecorded deviations from intended interventions

b. A large amount of inconsistency (83%) based on I-squared observed

c. Major asymmetry suggestive of small-study effects (publication bias, etc.) observed based on an LFK index of 2.17.

d. A large amount of inconsistency (63.9%) based on I-squared was observed

e. The 95% confidence interval of the effect size included zero

Table S7: The 12-item Template for Intervention Description and Replication

Authors	Condition	Item1	Item2	Item3	Item4	Item5	Item6	Item7	Item8	Item9	Item10	Item11	Item12
**Backman et al. 2014**	Intervention	Yes	Yes	No	Yes	Yes	Yes	Unclear	Yes	n/a	n/a	Yes	No
	Control	Yes	Yes	No	Yes	n/a	n/a	n/a	n/a	n/a	n/a	n/a	n/a
**Bourke et al. 2011**	Intervention	Yes	Yes	No	Yes	Yes	Yes	Yes	Yes	n/a	n/a	Yes	Yes
	Control	Yes	Yes	n/a	Yes	n/a	n/a	n/a	n/a	n/a	n/a	n/a	n/a
**Brown et al. 2018**	Intervention	Yes	Yes	Unclear	Yes	Yes	Yes	Yes	Yes	Yes	Yes	Yes	Yes
	Control	Yes	Yes	Yes	Yes	n/a	n/a	n/a	n/a	n/a	n/a	n/a	n/a
**Carli et al. 2020**	Intervention	Yes	Yes	Yes	Yes	Yes	Yes	Yes	Yes	Yes	No	Yes	Yes
	Control	Yes	Yes	Yes	Yes	Yes	Yes	Yes	Yes	Yes	No	Yes	No
**Christensen et al.2019**	Intervention	Yes	Yes	Unclear	Yes	No	Unclear	No	No	Yes	Yes	Yes	Yes
	Control	Yes	Yes	n/a	Yes	n/a	n/a	n/a	n/a	n/a	n/a	n/a	n/a
**Courneya et al. 2003**	Intervention	Yes	Yes	n/a	Yes	n/a	Unclear	Yes	Yes	Yes	n/a	Yes	No
	Control	Yes	Yes	n/a	Yes	n/a	n/a	n/a	n/a	n/a	n/a	n/a	n/a
**Courneya et al. 2016**	Intervention	Yes	Yes	Yes	Yes	Yes	Yes	Yes	Yes	Yes	Yes	Yes	Yes
	Control	Yes	Yes	No	Yes	n/a	Yes	n/a	No	n/a	n/a	No	No
**Demark-Wahnefried et al. 2012**	Intervention	Yes	Yes	Yes	Yes	Yes	Yes	Yes	Yes	Yes	No	No	No
	Control	Yes	Yes	Yes	Yes	Yes	Yes	Yes	Yes	Yes	No	No	No
**Hawkes et al. 2013**	Intervention	Yes	Yes	No	Yes	Yes	Yes	Unclear	Yes	Yes	n/a	No	Yes
	Control	Yes	Yes	Yes	Yes	n/a	n/a	n/a	n/a	n/a	n/a	n/a	n/a
**Young Kim et al. 2019**	Intervention	Yes	Yes	No	Yes	Yes	Yes	Yes	Yes	Yes	n/a	No	No

	Control	Yes	Yes	n/a	Yes	n/a	n/a	n/a	n/a	n/a	n/a	n/a	n/a
**Mi Kyung Lee et al.2017**	Intervention	Yes	Yes	No	Yes	Yes	Yes	Yes	Yes	Yes	n/a	No	No
	Control	Yes	Yes	n/a	Yes	n/a	n/a	n/a	n/a	n/a	n/a	n/a	n/a
**Mi Kyung Lee et al.2018**	Intervention	Yes	Yes	Yes	Yes	Unclear	Yes	Yes	Yes	No	n/a	Yes	No
	Control	Yes	Yes	No	Yes	Yes	Yes	Yes	Yes	Yes	n/a	No	No
**Mayer et al. 2018**	Intervention	Yes	Yes	Yes	Yes	Yes	Yes	Yes	Yes	Yes	n/a	Yes	No
	Control	Yes	Yes	No	No	n/a	n/a	n/a	n/a	n/a	n/a	n/a	n/a
**Pinto et al. 2013**	Intervention	Yes	Yes	No	Yes	Unclear	Yes	Yes	Yes	Yes	Yes	Yes	No
	Control	Yes	Yes	Unclear	Yes	Yes	Yes	Unclear	Yes	n/a	n/a	n/a	n/a
**Van Blarigan et al. 2019**	Intervention	Yes	Yes	Yes	Yes	n/a	Yes	Yes	Yes	n/a	n/a	Yes	Yes
	Control	Yes	Yes	No	Yes	n/a	n/a	n/a	n/a	n/a	n/a	n/a	n/a
**Van Vulpen et al. 2016**	Intervention	Yes	Yes	n/a	Yes	Yes	Yes	Yes	Yes	Yes	Yes	Yes	Yes
	Control	Yes	Yes	n/a	Yes	n/a	n/a	n/a	n/a	n/a	n/a	n/a	n/a
**Van Waart et al. 2018**	Intervention	Yes	Yes	No	Yes	Yes	Yes	Yes	Yes	Yes	n/a	Yes	Yes
	Control	Yes	Yes	No	No	n/a	n/a	n/a	n/a	n/a	n/a	n/a	n/a
**Watson et al. 2015**	Intervention	Yes	Yes	Yes	Yes	Yes	Yes	Yes	Yes	Yes	Yes	Yes	Yes
	Control	Yes	Yes	n/a	Yes	n/a	n/a	n/a	n/a	n/a	n/a	n/a	n/a
**Molenaar et al. 2023**	Intervention	Yes	Yes	Yes	Yes	Yes	Yes	Yes	Yes	Yes	No	Yes	Yes
	Control	Yes	Yes	Yes	Yes	Yes	Yes	Yes	Yes	Yes	No	Yes	No

Item1: Provide the name or a phrase that describes the intervention
Item2: Describe any rationale, theory, or goal of the elements essential to the intervention.
Item3: Materials - Describe any physical or informational materials used in the intervention
Item4: Procedures - Procedures: Describe each of the procedures, activities, and/or processes used in the intervention.

Item5: For each category of intervention provider (e.g. psychologist, nursing assistant), describe their expertise, background and any specific training given
Item6: Describe the modes of delivery
Item7: Describe the type(s) of location(s) where the intervention occurred
Item8: Describe the number of times the intervention was delivered and over what period of time
Item9: If the intervention was planned to be personalized, titrated or adapted, then describe what, why, when and how
Item10: If the intervention was modified during the course of the study, describe the changes (what, why, when and how)
Item11: Planned: If intervention adherence or fidelity was assessed, describe how and by whom.
Item12: Actual: If intervention adherence or fidelity was assessed, describe the extent to which the intervention was delivered as planned

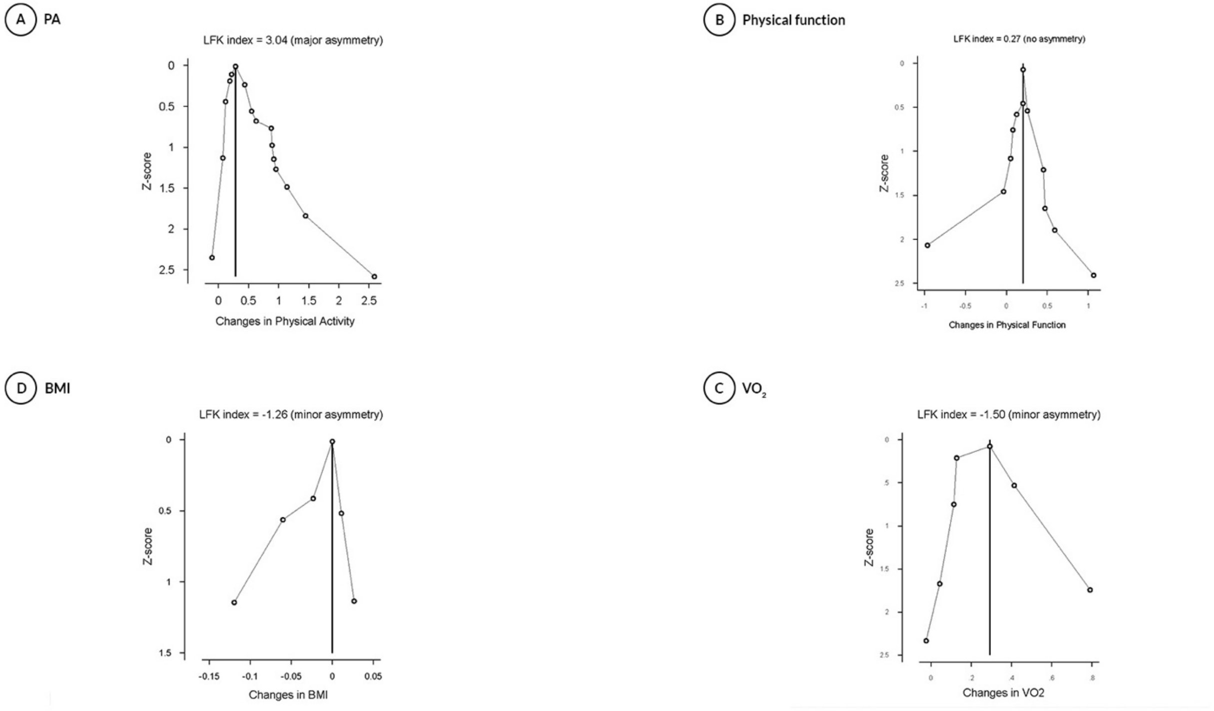

Figure S3: Doi plot for changes in A) PA, B) Physical function, C) VO_2, D) and BMI.

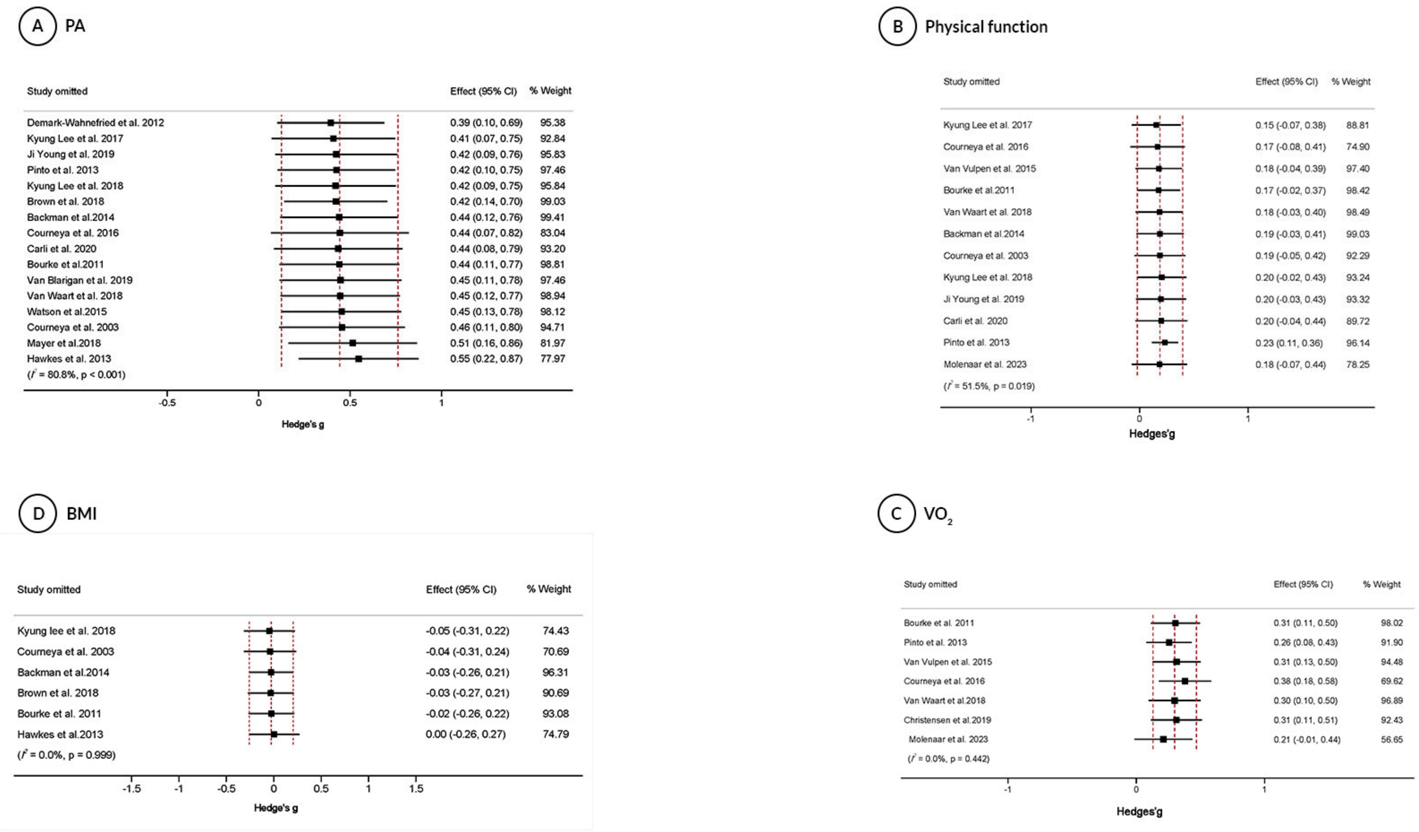

Figure S4: Influence meta-analysis for changes in A) PA, B) Physical function, C) VO_2, D) and BMI.

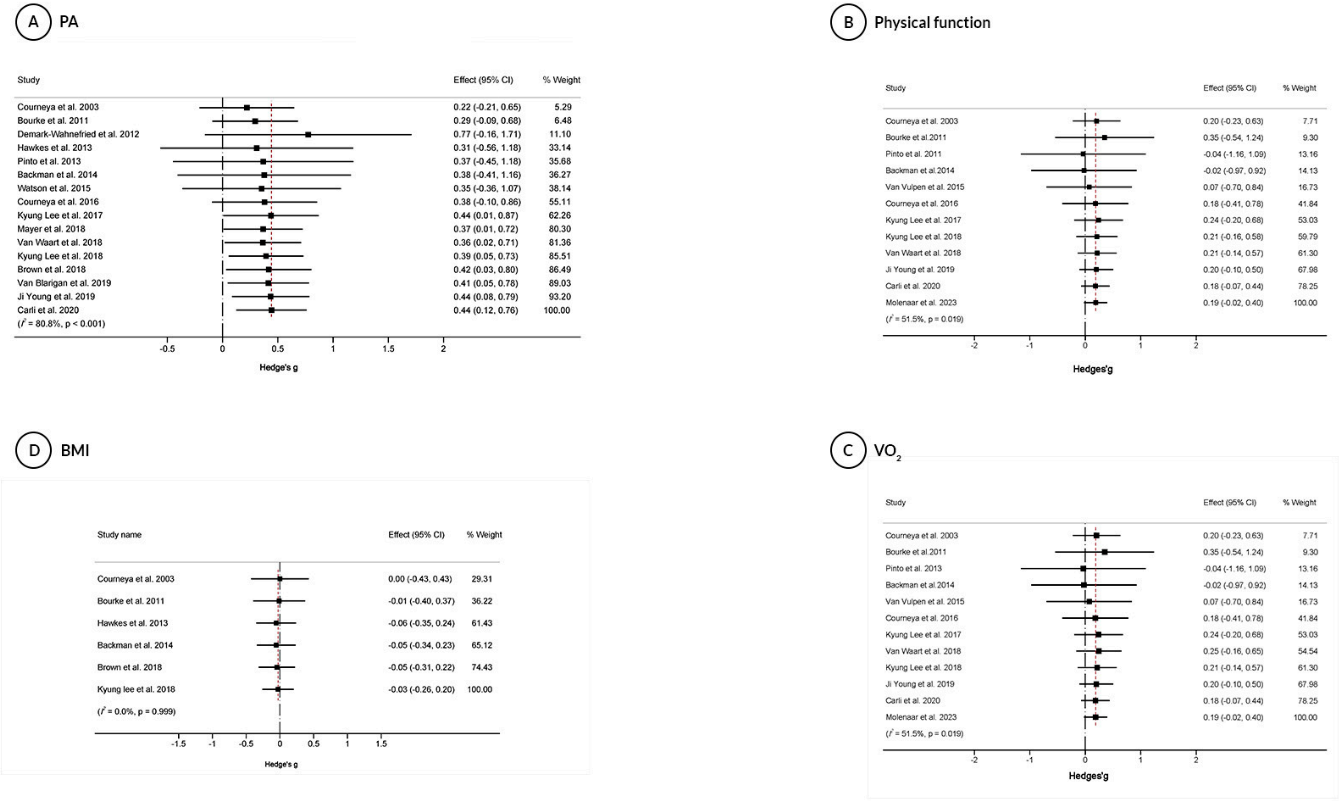

Figure S5: Cumulative meta-analysis for changes in A) PA, B) Physical function, C) VO_2, D) and BMI.

Table S8: Magnitude of difference between atheoretical and theoretical subgroups, PIs (changes in PA)									
**Subgroup**	**ES**	**Diff**	**SE (ES)**	**Variance (ES)**	**SEDiff**	**LLDiff**	**ULDiff**	**Zdiff**	**p (Zdiff)**
**Atheoretical**	0.60	0.33	0.23	0.053	0.23	-0.12	0.78	1.43	0.23
**Theoretical**	0.27		0.18	0.00019					

	**ES**	**95%CI**	**95%PI**
**Atheoretical**	0.60	0.15, 1.04	-0.68, 1.88
**Theoretical**	0.27	-0.08, 0.62	-0.62, 1.16

Notes: Diff: Magnitude of difference; SE: standard error; UL: Upper 95% limit; LL: lower 95% limit; PI: prediction intervals; CI: confidence intervals; ES: effect size

Table S9: Magnitude of difference between atheoretical and theoretical subgroups, PIs (changes in physical function)									
**Subgroup**	**ES**	**Diff**	**SE (ES)**	**Variance (ES)**	**SEDiff**	**LLDiff**	**ULDiff**	**Zdiff**	**p (Zdiff)**
**Atheoretical**	0.21	0.06	0.099	0.0098	0.18	-0.29	0.41	0.33	0.22
**Theory**	0.15		0.47	0.023					

	**ES**	**95%CI**	**95%PI**
**Atheoretical**	0.21	0.012, 0.40	-0.15, 0.57
**Theoretical**	0.15	-0.77, 1.07	-3.10, 3.40

Notes: Diff: Magnitude of difference; SE: standard error; UL: Upper 95% limit; LL: lower 95% limit; PI: prediction intervals; CI: confidence intervals; ES: effect size

Table S10: Simple meta-regression results for changes in PA.

Variable	$\beta_1 \pm$ SE	CI (95%)	p	Q	I^2 (%)
**Study characteristics**					
Year of publication	0.025±0.057	-0.096, 0.15	0.66	91.36***	84.68
Journal impact factor	0.038±0.049	-0.14, 0.067	0.45	92.51***	84.87
Country (Sweden)	0.407±0.409	-0.519, 1.334	0.346	65.48	86.26
Country (UK)	0.333±0.525	-1.52, 0.854	0.541	65.48	86.26
Country (Canada)	-0.0878±0.415	-1.026, 0.851	0.837	65.48	86.26
Country (Australia)	0.438±0.409	-1.36, 0.489	0.313	65.48	86.26
Country (South Korea)	-0.322±0.409	-1.25, 0.605	0.425	65.48	86.26
Country (The Netherlands)	0.392±0.410	-0.536, 1.32	0.364	65.48	86.26
Country (U.S. vs. Others)	0.101±0.354	-0.659, 0.862	0.7787		84.74
RoB (Some concerns)	0.398±0.191	-0.0143, 0.81	0.0011	67.58	80.76
RoB (High)	0.637±0.131	0.354, 0.921	0.0011	67.58	80.76
Use of theory	-0.611±0.184	-1.01, 0.217	0.005	59.36	76.42
TIDiER_int	-0.00687±0.586	-0.132, 0.12	0.9083	92.51	84.87
TIDiER_cont	0.0934±0.045	-0.0027, 0.189	0.0559	74.06	81.10
Sample size	-0.00218±0.00053	-0.00333, -0.00104	0.0011	60.18	76.74
**Participants characteristics**					
Age	-0.0022±0.188	-0.0425, 0.0381	0.9085	92.48	84.86
Male (%)	-3.05±1.37	-5.99, -0.116	0.0427	77.90	82.03
Education	0.281±0.450	-0.684, 125	0.5422	89.01	84.27
Cancer site (colon %)	0.467±0.197	0.0421, 0.893	0.034	92.53	84.87
Cancer Stage (Tumour II)	-0.0199±0.00575	-0.0322, -0.00754	0.0039	91.94	84.77
Cancer Stage (Tumour III/IV)	-0.225±0.163	-0.576, 0.125	0.1895	91.36	84.68
Risky behaviors	0.531±0.172	0.162, 0.900	0.0081	72.08	80.58
**Intervention characteristics**					
Type_aerobic	0.502±0.133	0.215, 0.788	0.0022	68.70	79.62
Type_strength	0.542±0.171	0.175, 0.909	0.0069	67.79	79.35
Delivery_supervised	-0.00262±0.189	-0.508, 0.403	0.9891	92.55	84.87
Delivery_unsupervised	0.324±0.25	-0.21, 0.86	0.214	82.24	82.98
Setting_home	-0.0106±0.185	-409, 0.388	0.9551	82.81	83.09
Setting_healthcare	0.389±0.171	0.0209, 0.757	0.0398	81.80	82.88
Intervention personnel	-0.0464±0.281	-0.650, 0.557	0.8712	89.41	84.34
Objective assessment	0.330±0.396	-0.518, 1.178	0.4176	92.24	84.82
Length of intervention	-0.000123±0.0050	-0.018, 0.0106	0.9806	43.01	81.40
Frequency	0.120±0.0371	0.0395, 0.199	0.0063	69.95	79.99
Duration	0.0113±0.0048	0.00101, 0.0216	0.0335	74.98	81.33
Mode_in person	-0.00262±0.189	-0.408, 0.403	0.9891	92.55	84.87

Mode_prescription	-0.138±0.273	-0.724, 0.448	0.6219	90.81	84.58
Mode_counselling	-0.198±0.0243	-0.0234, 0.0811	0.4044	88.99	84.27
Intervention personel trained	-0.046±0.28	-0.65, 0.56	0.871	92.35	84.84
Number of BCTs	0.0289±0.0243	-0.0233, 0.0811	0.256	86.62	83.84
**Outcomes**					
Physical function	0.0071±0.0073	-0.00852, 0.0227	0.3468	89.33	84.33
**BCTs**					
Goal setting _behavior	0.439±0.126	0.171, 0.709	0.003	92.55	83.79
Goal setting_outcome	-0.183±-0.299	-0.824, 0.459	0.551	89.93	84.41
Action planning	0.60±0.371	-0.195, 1.40	0.128	74.96	81.32
Instructions on how to perform behavior	-0.489±0.152	-0.815, -0.163	0.006	69.34	79.81
Self-monitoring of behavior	0.0495±0.236	-0.456, 0.555	0.836	92.43	84.85
Health consequences	0.286±0.276	-0.306, 0.880	0.318	87.46	83.99
Review behavior goal	-0.103±0.250	-0.638, 0.432	0.686	91.57	84.71
Problem solving	-0.166±0.240	-0.680, 0.350	0.501	90.09	84.46
Persuasive argument	0.133±0.277	-0.460, 0.728	0.637	90.86	84.59
Graded tasks	-0.321±0.212	-0.777, 0.135	0.154	83.17	83.17
Feedback on behavior	0.041±0.257	-0.510, 0.593	0.874	92.39	84.85
Prompts/cues	-0.656±0.169	-1.02, -0.293	0.002	62.81	77.71
Modeling of behavior	0.0322±0.280	-0.565, 0.630	0.910	92.47	84.86
Generalization of target behavior	-0.346±0.200	-0.776, 0.0840	0.106	86.73	83.86
Restructuring the physical environment	-0.297±0.291	-0.921, 0.327	0.325	86.25	83.77

Notes: RoB: risk of bias categories (some concerns and high with low as reference); TIDiER_int: TIDiER score for intervention groups; TIDiER_int: TIDiER_cont: TIDiER score for control groups;Type_aerobic: type of exercises applied during intervention (in this case aerobic); delivery_supervised: intervention delivery (in this case supervised); Setting_hom: settings of intervention (in this case at home); Mode_in person: intervention mode (in this case in person)
